# Possible involvement of three-stemmed pseudoknots in regulating translational initiation in human mRNAs

**DOI:** 10.1371/journal.pone.0307541

**Published:** 2024-07-22

**Authors:** Xiaolan Huang, Zhihua Du

**Affiliations:** 1 School of Computing, Southern Illinois University at Carbondale, IL, United States of America; 2 School of Chemical and Biomolecular Sciences, Southern Illinois University at Carbondale, IL, United States of America; New York University, UNITED STATES OF AMERICA

## Abstract

RNA pseudoknots play a crucial role in various cellular functions. Established pseudoknots show significant variation in both size and structural complexity. Specifically, three-stemmed pseudoknots are characterized by an additional stem-loop embedded in their structure. Recent findings highlight these pseudoknots as bacterial riboswitches and potent stimulators for programmed ribosomal frameshifting in RNA viruses like SARS-CoV2. To investigate the possible presence of functional three-stemmed pseudoknots in human mRNAs, we employed in-house developed computational methods to detect such structures within a dataset comprising 21,780 full-length human mRNA sequences. Numerous three-stemmed pseudoknots were identified. A selected set of 14 potential instances are presented, in which the start codon of the mRNA is found in close proximity either upstream, downstream, or within the identified three-stemmed pseudoknot. These pseudoknots likely play a role in translational initiation regulation. The probability of their existence gains support from their ranking as the most stable pseudoknot identified in the entire mRNA sequence, structural conservation across homologous mRNAs, stereochemical feasibility as demonstrated by structural modeling, and classification as members of the CPK-1 pseudoknot family, which includes many well-established pseudoknots. Furthermore, in four of the mRNAs, two or three closely spaced or tandem three-stemmed pseudoknots were identified. These findings suggest the frequent occurrence of three-stemmed pseudoknots in human mRNAs. A stepwise co-transcriptional folding mechanism is proposed for the formation of a three-stemmed pseudoknot structure. Our results not only provide fresh insights into the structures and functions of pseudoknots but also unveil the potential to target pseudoknots for treating human diseases.

## Introduction

Pseudoknots are formed when a sequence of nucleotides within a loop region binds to a complementary sequence outside that very loop [1, 2]. Essentially, by adopting this broad definition of pseudoknots, any tertiary canonical base-pairing interaction would be classified as a pseudoknot. In cases where the loop originates from a hairpin, the resulting pseudoknot is dubbed an H-type pseudoknot. In the PseudoBase and its extension PseudoBase++ [3, 4], pseudoknots of the H-type category account for 81% of the 398 pseudoknots in the database. Due to its common occurrence and significance in RNA functions, the H-type pseudoknot has become the archetype for pseudoknots. As a result, the term "pseudoknot" is often used interchangeably with "H-type pseudoknot". Herein, when we refer to pseudoknots, we’re talking about H-type pseudoknots unless otherwise specified.

A pseudoknot possesses essential structural components, including two helical stems (designated as S1 and S2) and two distinct loops (L1 and L2, with L1 traversing the major groove of S2 and L2 traversing the minor groove of S1). In certain instances, a pseudoknot may include a third loop known as L3, positioned between the two stems. In situations where L3 is absent, the two stems, S1 and S2, have the potential to stack together coaxially, forming a quasi-continuous helical structure. These common structural features of H-type pseudoknots are illustrated in various NMR and crystal structures, as discussed in reviews by Hill and Brierley, as well as Peselis and Serganov [5, 6].

Pseudoknots frequently reside in pivotal regions within RNA molecules, serving a wide array of functions in various biological processes [5–7]. Notably, pseudoknots are renowned for their participation in translational recoding mechanisms, including −1 programmed ribosomal frameshifting (PRF) and stop codon readthrough (SCR) [8, 9].

In −1 PRF, the translating ribosome shifts one nucleotide backward upon encountering a specific hepta-nucleotide sequence known as the "slippery sequence." Following this shift, the ribosome proceeds with the translation process within the −1 reading frame [9, 10]. In SCR, the translating ribosome treats the stop codon as if it were a sense codon and continues the translation process [11, 12]. In numerous documented occurrences of −1 PRF and SCR, efficient frameshifting or stop codon readthrough often requires the presence of a pseudoknot positioned several nucleotides downstream from the recoding site [1, 2, 13–23].

Stimulating pseudoknots associated with −1 PRF or SCR have exhibited significant variation in both size and structural complexity. The majority of these pseudoknots typically contain the two basic helical stems required for pseudoknot formation and 2–3 connecting loops. Notable examples include the −1 PRF stimulating pseudoknots found in plant Luteoviruses such as beet western yellows virus (BWYV) and related viruses [15, 24–26], and in Simian Retrovirus type 1 (SRV-1) and its close relatives [27–29], as well as the SCR-stimulating pseudoknots present in Moloney Murine Leukemia Virus (Mo-MuLV) and related viruses, [11, 12, 30, 31].

**Fig 1:**
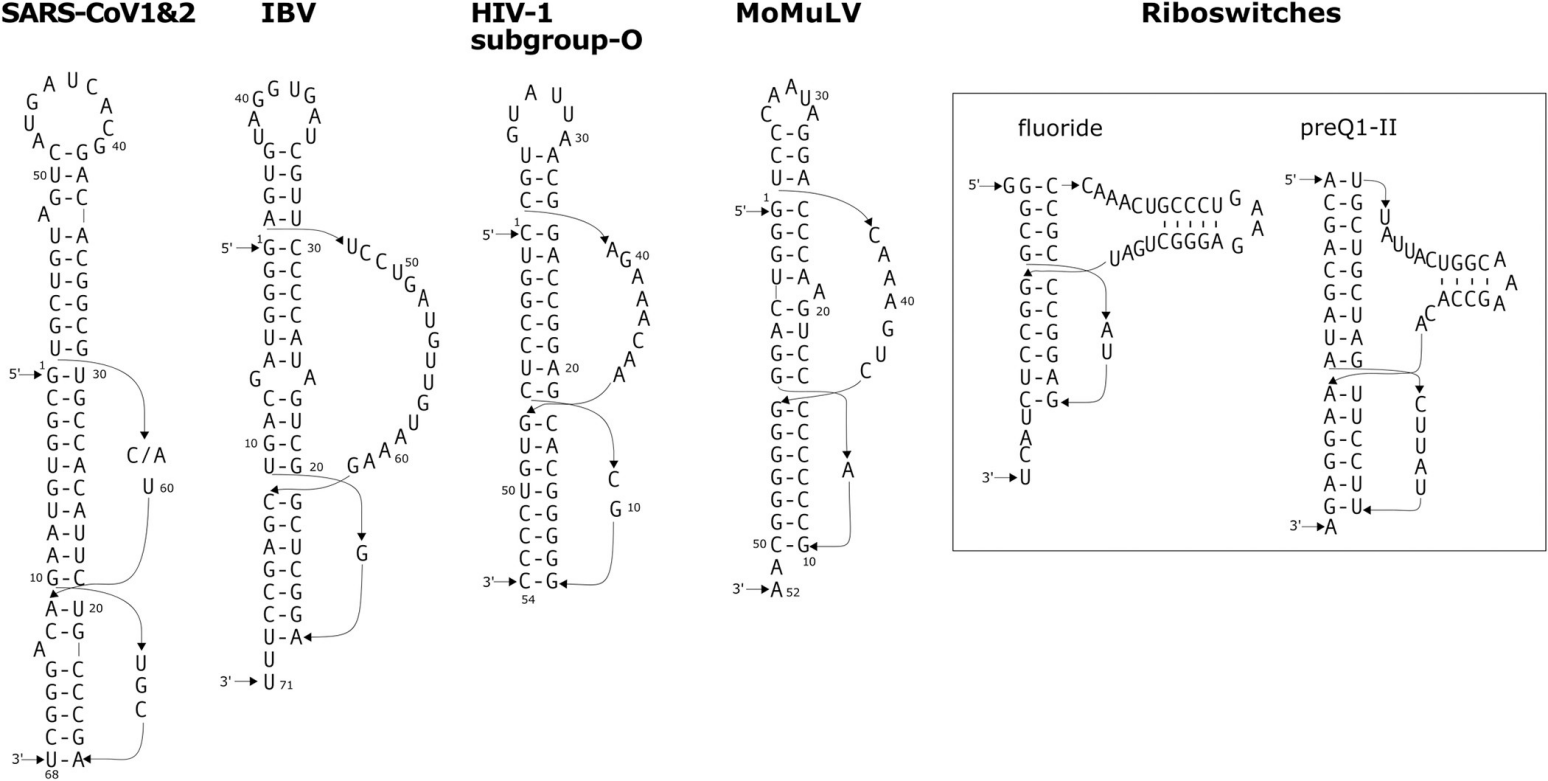
Previously reported three-stemmed pseudoknots. The three-stemmed pseudoknots were identified in different RNA molecules, including the frameshift stimulating pseudoknot at the ORF1a and ORF1b junction of the coronaviruses of SARS-CoV-1 and SARS-CoV-2 viruses (the two viral sequences have only one nucleotide difference C/A in the loop), as well as Infectious bronchitis virus (IBV), the frameshift stimulating pseudoknot at the *gag-pol* junction of HIV-1 subgroup-O viruses, the stop-codon readthrough stimulating pseudoknot in MoMuLV, the fluoride riboswitch, and the preQ1–II riboswitch. The numbering of the SARS-CoV-1/2, IBV, HIV-1, and MoMuLV pseudoknots is arbitrary, beginning with the first nucleotide of the displayed sequences. Every 10th nucleotide is indicated to assist in easily tracing the folding of the molecules.

More recently, there has been an increasing recognition of the presence of elaborate structural features in certain viral −1 PRF stimulating pseudoknots. One instance of this phenomenon was observed within HIV-1 subgroup-O viruses [32]. It was proposed that −1 PRF at the junction of the *gag* and *pol* genes was stimulated by an elaborated pseudoknot that contained an extra stem with three base-pairs within the loop2 sequence (Fig 1). Computational simulations conducted by the authors demonstrated that the additional three-base pair stem, as well as the two pseudoknot-forming stems (stem1 and stem2), possessed the potential for coaxial stacking.

Subsequently, similar pseudoknots of this nature were proposed to function as −1 PRF stimulating pseudoknots within the overlapping region of open reading frames ORF1a and ORF1b in the severe acute respiratory syndrome coronavirus 1 (SARS-CoV-1) and related coronaviruses (Fig 1) [22]. These pseudoknots were referred to as three-stemmed pseudoknots. According to the classification of pseudoknots per type (pseudoknot type) proposed by Han and Byun [33], which is adopted in PseudoBase++ [4], these pseudoknots belong to type HL_out. Pseudoknots with the most basic configuration that contains two stems (stem1 and stem2) and two or connecting loops (loop1, loop2, and optionally loop3) are classified as type H. Compared to type H pseudoknots, type HL_out pseudoknots have one or more additional stems within either the loop1 region, the loop2 region, or both. The pseudoknots classified as type H and type HL_out by Han and Byun [31] fall under the commonly recognized H-type pseudoknot category. There are 64 pseudoknots classified as type HL_out in PseudoBase++. Among these, 40 contain one or two additional stems in the loop2 region, with one additional stem being the most common. In this paper, the term "three-stemmed pseudoknot" refers to pseudoknots that belong to the common H-type pseudoknot category (or type HL_out if using the Han and Byun classification) and harbor an additional stem-loop structure within the loop2 region, unless stated otherwise.

The causative agent of the COVID-19 pandemic, severe acute respiratory syndrome coronavirus 2 (SARS-CoV-2), is closely related to SARS-CoV-1 [34]. Extensive research has been conducted on this virus in recent years. Notably, the −1 PRF signals in SARS-CoV-2 are very similar to those in SARS-CoV-1, with their frameshift-stimulating pseudoknots differing by only a single nucleotide in loop2 [34] (see Fig 1). Consequently, the pseudoknot structure of SARS-CoV-2 also possesses the capability to assume a three-stemmed configuration. Recent structural investigations employing X-ray crystallography and Cryo-EM techniques have substantiated the existence of the three-stemmed frameshift-stimulating pseudoknot in SARS-CoV-2 [35–38].

Three-stemmed pseudoknots have also been identified in various other RNAs, as evidenced by many examples in PseudoBase++ [4]. Among these examples are two found in riboswitches: the fluoride riboswitch and the preQ1–II riboswitch. Each of these riboswitch pseudoknots includes an additional hairpin crucial for ligand binding [39, 40] (Fig 1).

Pseudoknots featuring additional stems might be more widespread than presently understood. In our recent investigation [41], which involved a reevaluation of previously documented −1 PRF or SCR-inducing pseudoknots to assess their capacity to accommodate additional structural elements, we uncovered that many of these pseudoknots indeed possess the potential to contain various extra structural elements. These include stem-loop formations, nested pseudoknots, interlocking hairpins, and additional loop-loop interactions. Notably, these elaborated pseudoknots were identified across diverse virus families utilizing either the −1 PRF or SCR recoding mechanisms [41]. Two examples of the potential three-stemmed pseudoknots re-evaluated in the study are shown in Fig 1, including the frameshift stimulating pseudoknot in Infectious bronchitis virus (IBV) and the stop-codon readthrough stimulating pseudoknot in Moloney Murine Leukemia Virus (MoMuLV). These two pseudoknots are classified as simple H type pseudoknots in PseudoBase++. These pseudoknots were studied previously [42–44], but the potential presence of an extra stem in the pseudoknots was not recognized.

The majority of reported pseudoknots are primarily found within viral or bacterial RNAs. There is only a limited number of instances where pseudoknots have been identified in human mRNAs or more broadly, within eukaryotic cellular mRNAs. The PseudoBase and PseudoBase++ databases [3, 4] register 398 pseudoknots, but only 12 pseudoknots are found in eukaryotic mRNAs, including six homologous interferon gamma (IFNG) mRNAs from human and other species [45], two homologous human and cow prion protein mRNAs [46], one human CCR5 mRNA [47], one rat antizyme mRNA, one human *PEG10 (*paternally expressed gene 10) mRNA, and one human *Ma3* mRNA. The pseudoknot in IFNG mRNA is located in the 5’ untranslated region (UTR). It activates protein kinase RNA-activated (PKR), which in turn phosphorylates the alpha chain of the initiation factor eIF2. This phosphorylation enhances the translation efficiency of IFNG mRNA. In all other cases, the pseudoknot is located in the coding region. Apart from the pseudoknots found in prion protein mRNAs, the remaining pseudoknots are involved in regulating ribosomal frameshifting. PEG10 and Ma3 mRNAs are transcribed from retroviral-derived genes and they encode viral-like proteins. As a result, their −1 frameshifting signals, including the slippery sequences and the stimulating pseudoknots, bear a resemblance to those in viral mRNAs. In PseudoBase++, these mRNAs are actually classified as viral mRNAs. Overall, it is evident that the documented cases of pseudoknots in human mRNAs are quite limited. This is in stark contrast to the widespread occurrence of pseudoknots in viral and bacterial mRNAs.

Numerous molecular mechanisms and structures were originally elucidated within the context of viral biology before their counterparts were recognized in mammalian cellular systems [48]. While the number of reported pseudoknots in eukaryotic mRNAs is very limited, these cases nonetheless demonstrate that functional pseudoknots do exist and play significant roles in various cellular processes. Despite their rarity, the observed pseudoknots contribute to critical functions such as regulation of gene expression, ribosomal frameshifting, and the stabilization of RNA structures. These findings underscore the importance of pseudoknots in the intricate machinery of eukaryotic cells and suggest that further research may uncover additional examples.

In PseudoBase++, all documented pseudoknots in eukaryotic mRNAs are classified as type H, the simplest form with a basic pseudoknot configuration. To date, pseudoknots with elaborate structural features, such as three-stemmed pseudoknots, have not been identified in eukaryotic mRNAs. However, our recent research on viral pseudoknots with more complex structures has led us to realize that these types of pseudoknots may be more common than previously thought and may not be limited to viral RNAs [41]. We hypothesize that eukaryotic mRNAs could also contain complex pseudoknots with specific functional roles. To test this hypothesis, we have initiated research focused on identifying potential three-stemmed pseudoknots in human mRNAs.

To identify potential pseudoknots within full-length mRNAs without any assumptions about their specific locations, it is crucial to utilize computational tools capable of handling pseudoknot identification in long RNA sequences, regardless of their length. These tools must also be highly efficient due to the need to analyze a vast number of mRNAs. In this study, a dataset of 21,780 full-length human mRNA sequences was examined.

Many programs are available for identifying pseudoknots, including IPknot [49], Hotknot [50], Dotknot [51], Probknot [52], KineFold [53], vsfold5 [54], CyloFold [55], pKiss [56], Vfold2D [57], TrRosettaRNA [58], and MC-FOLD [59]. Each of these programs has an upper limit on the input RNA sequence, ranging from 100 to 1000 nucleotides. To evaluate their performance in identifying pseudoknots in longer RNA sequences, we tested them using a 740-nucleotide sequence from simian retrovirus type 1 (SRV-1). This sequence includes a well-established pseudoknot (with a basic configuration containing two stems and two loops) that stimulates efficient −1 programmed ribosomal frameshifting (−1 PRF) [27–29]. The only program capable of accurately identifying the established SRV-1 pseudoknot from the 740-nucleotide input sequence is pKiss, which required approximately 8 minutes to complete the analysis. Given that these programs have an upper limit of 1000 or fewer nucleotides, they are unsuitable for identifying potential pseudoknots in full-length viral or cellular mRNA sequences, which typically contain thousands or even tens of thousands of nucleotides. For example, the SRV-1 genomic mRNA (M11841) contains 8,173 nucleotides, while coronaviruses have nearly 30,000 nucleotides in their genomic mRNAs.

We have developed computational tools for identifying potential pseudoknots and related RNA structures, with the core program named PKscan. PKscan can detect all potential pseudoknots within any RNA sequence, regardless of its length. Using PKscan with the following default settings for the ranges of stems and loops (which users can modify): S1 = 5 to 20 bp, S2 = 5 to 10 bp, L1 = 1 to 10 nt, L2 = 5 to 35 nt, and L3 = 0 to 1 nt, it took less than 10 seconds to identify all potential pseudoknots from the full-length genomic mRNA of SRV-1. All potential pseudoknots that meet the search criteria are listed in order of the calculated free energy of their stems. The previously established SRV-1 pseudoknot is identified as the highest-ranking pseudoknot (S3 File).

The unparalleled effectiveness of PKscan in identifying potential pseudoknots in long RNA sequences makes our computational tools highly suitable for large-scale studies aimed at detecting pseudoknots in full-length mRNA datasets, as demonstrated in our previous works [60, 61]. In this study, we analyzed a dataset comprising 21,780 full-length human mRNA sequences to identify potential three-stemmed pseudoknots. To accomplish this, we enhanced the PKscan program with additional functionalities to detect the presence of an extra stem within loop2 of the identified pseudoknots.

Numerous potential cases of three-stemmed pseudoknots that meet the search criteria were identified. They are found throughout all regions of the mRNAs, including 5’UTR, the coding region, and 3’UTR. The search results have been uploaded to GitHub (https://github.com/xhuang123/Data/blob/main/Three-Stemmed-PKs). Herein, we selected 14 cases of potential three-stemmed pseudoknots identified in close proximity to the AUG start codon in the mRNA sequence for further detailed analysis and presentation. These potential pseudoknots are found in the mRNAs of Malassezia restricta dynactin 4 (MRET_4255), chromodomain helicase DNA binding protein 5 (CHD5), CTD small phosphatase like (CTDSPL), mab-21 like 4 (MAB21L4), sodium voltage-gated channel beta subunit 1 (SCN1B), protein phosphatase 2 catalytic subunit beta (PPP2CB), MYCL proto-oncogene bHLH transcription factor (MYCL), kelch like family member 2 (KLHL2), dynein axonemal intermediate chain 1 (DNAI1), slit guidance ligand 2 (SLIT2), biphenyl hydrolase like (BPHL), transmembrane protein 181 (TMEM181), small integral membrane protein 10 like 2B (SMIM10L2B), and small ArfGAP2 (SMAP2). The protein products derived from these mRNAs serve crucial functional roles, with many of them directly linked to various diseases [62–67].

Experimental studies are essential to validate the presence of these predicted pseudoknots and elucidate their biological roles. Nonetheless, several lines of evidence support their credibility. Firstly, these potential pseudoknots are among the most stable, as indicated by the calculated free energy of the two essential stems (excluding potential contribution from the additional stem) identified throughout the mRNA sequence. This suggests the potential importance of the predicted pseudoknot in the mRNA. Secondly, sequence comparisons indicate that homologous mRNA sequences can adopt similar 2D structures. Structural modeling further confirms the stereochemical feasibility of these predicted structures. Lastly, all these potential pseudoknots belong to the CPK-1 (common pseudoknot motif-1) family [68, 69], which is well-documented in both naturally occurring and SELEX-derived pseudoknots, underscoring their prevalence and evolutionary significance [70–72].

It is also interesting to note that 4 out of the 14 selected mRNAs have sequences with the potential to form not just one, but two or even three closely spaced or tandem three-stemmed pseudoknots. These findings suggest that three-stemmed pseudoknots may frequently occur in human mRNAs.

To date, only a small number of pseudoknots have been identified in human mRNAs, according to the PseudoBase and PseudoBase++ databases [3, 4]. Notably, no three-stemmed pseudoknots have been documented. Given this context, the findings presented here mark substantial progress that will likely drive further exploration into the potential role of pseudoknots in regulating the translation of human mRNAs. Identifying and validating pseudoknots as regulatory RNA structures within human mRNAs not only promises a deeper comprehension of the mechanisms governing translational processes but also unveils promising targets for innovative drug development strategies.

The discovery of potential three-stemmed pseudoknots within human mRNAs also holds significant implications for the development of antiviral drugs targeting the SARS-CoV-2 frameshift-stimulating three-stemmed pseudoknot. Recent studies have demonstrated the efficacy of small molecules and antisense oligonucleotides (ASOs) in targeting the frameshift-stimulating pseudoknot in SARS-CoV-2. These agents have demonstrated substantial inhibition of −1 PRF in frameshifting assays, resulting in a notable reduction in SARS-CoV-2 virus replication within cultured cells [34, 73–75]. As the possible presence of structurally similar three-stemmed pseudoknots in human RNAs is now recognized, it becomes crucial to assess whether potential drugs designed for the SARS-CoV-2 pseudoknot could inadvertently affect similar RNA structures within human RNAs. This preemptive action aims to mitigate off-target effects in the development of antiviral treatments—an aspect that was previously overlooked.

## Results

While many different possibilities exist for how an elaborated pseudoknot can manifest its additional structural features alongside the mandatory stems and loops, this study is primarily dedicated to the identification of potential pseudoknots featuring an additional stem within the loop2 sequence. Furthermore, the computational method is constrained to potential pseudoknots characterized by the absence of gaps between the three stems, allowing them to potentially stack co-axially, forming a quasi-continuous helical structure, which is expected to contribute to enhanced stability of the overall structure. In the PseudoBase and PseudoBase++ databases [3, 4], there are only a few three-stemmed pseudoknots characterized by three stems that lack an intervening sequence between them. These include the −1 PRF stimulating pseudoknot in SARS-CoV-2 [19, 34]. The structures of the SARS-CoV-2 pseudoknot have recently been elucidated using X-ray crystallography [35, 36] and Cryo-EM [37, 38]. These studies have confirmed the presence of the additional stem. Interestingly, the overall shape of the pseudoknot, and the specific base-pairing pattern at the junction between stem1 and stem2, vary across different investigations. In the study by Jones et al. [36], the crystal structure reveals a coaxial stacking of the three stems, forming a quasi-continuous helix. Notably, this structure accommodates a single-stranded nucleotide at the junction region between stem1 and stem2. This structure demonstrates that a three-stemmed pseudoknot can form coaxial stacking among its stems, even when the stems contain structural irregularities.

To uncover potential pseudoknots, we employed our in-house developed pseudoknot detection tool, PK-Scan. PK-Scan boasts the ability to identify all potential pseudoknots within any given RNA sequence, regardless of its length, as demonstrated in our previous works [60, 61]. This distinctive feature of PK-Scan, capable of handling lengthy RNA sequences, aligns perfectly with the objectives of this research. Specifically, our aim is to identify potential pseudoknots within full-length mRNA sequences without making any assumptions regarding their specific location within the mRNA sequence.

When utilizing PK-Scan, users have the flexibility to specify the permissible length ranges for the stems and loops within pseudoknot structures. In the specific search round discussed in this paper, we configured the ranges for stem1 (S1), stem2 (S2), loop1 (L1), loop2 (L2), and loop3 (L3) as follows: S1 ranged from 5 to 20 base pairs, S2 ranged from 6 to 7 base pairs, L1 ranged from 1 to 2 nucleotides, L2 ranged from 5 to 50 nucleotides, and L3 was set to 0 nucleotide. These specified S2, L1, and L3 ranges signify that the search is limited to pseudoknots within the previously proposed pseudoknot family CPK1 (short for common pseudoknot motif 1). Pseudoknots within CPK1 exhibit consistent structural characteristics, including the presence of a minimal 1–2 nucleotide in L1 that traverses the major groove of S2, comprising 6–7 base pairs, and the occurrence of coaxial stacking between the two stems without any intervening loop. Past research has demonstrated the prevalence of CPK1 pseudoknots in both naturally occurring systems and SELEX pseudoknots, underscoring the widespread occurrence and popularity of these particular pseudoknot structures [70, 76, 77].

While pseudoknots are not exclusive to the CPK1 type, focusing solely on CPK1 pseudoknots may result in the oversight of other pseudoknot variants. Nevertheless, in this initial endeavor, the objective is not to comprehensively cover all possibilities but rather to identify potential instances of pseudoknots within human mRNAs, establishing a proof of concept. By initially narrowing the search to CPK1 pseudoknots, we can achieve greater efficiency while still meeting the ultimate goal, particularly considering the extensive dataset comprising 21,780 full-length human mRNA sequences.

The initial version of the PK-Scan program lacked the capability to assess the presence of an additional stem, referred to as stem3 (abbreviated as S3 hereafter). In response to the requirement for detecting three-stemmed pseudoknots, the program underwent enhancements to enable the evaluation of the potential formation of S3 subsequent to the identification of a putative pseudoknot.

When using PK-Scan to identify potential pseudoknots within a given RNA sequence, it identifies all potential pseudoknots. To assess the relative stability of these detected pseudoknots, the program conducts a free energy calculation on the stem regions (stem1 and stem2) of each pseudoknot. Subsequently, the program ranks the detected pseudoknots based on the calculated free energy values, providing a valuable assessment of their relative stability. Given PK-Scan’s capacity to identify all potential pseudoknots within the full-length mRNA sequence, this ranking offers valuable insights into the significance of the identified pseudoknots. It is important to note that in this study, the free energy calculation includes only stem1 and stem2 while excluding the additional stem3. In other words, when comparing the detected three-stemmed pseudoknot with other detected pseudoknots within the mRNA in terms of free energy, the comparison is solely based on the two stems that are required for pseudoknot formation.

The rationale behind these procedures is to ensure that the detection of a potential extra stem is limited to pseudoknots with a reasonably stable base structure. Pseudoknots with weak primary stems (stem1 and stem2) but a strong extra stem (stem3) should be discarded. If the energy calculation included all three stems, these pseudoknots would be identified due to contribution from the strong stem3. However, the likelihood for these pseudoknots to actually form is relatively low because of the weak primary stems.

The enhanced PK-Scan program was employed to identify three-stemmed pseudoknots that met the previously stated criteria within a dataset comprising 21,780 full-length human mRNA sequences sourced from the NCBI RefSeq database. We have detected a significant number of potential instances of three-stemmed pseudoknots. Out of the 21,780 sequences, potential three-stemmed pseudoknots that meet the search criteria were identified in 4,251 sequences. Moreover, multiple potential three-stemmed pseudoknots were detected in some of the sequences. Notably, many of these putative three-stemmed pseudoknots can be found in the vicinity of the start codon region. In some cases, the AUG start codon is directly embedded within the pseudoknot-forming sequence, while in other cases the start codon situates in close proximity, either upstream or downstream from the pseudoknot. This paper focuses on presenting and discussing this specific subset of three-stemmed pseudoknots that we have identified within human mRNAs.

**Fig 2:**
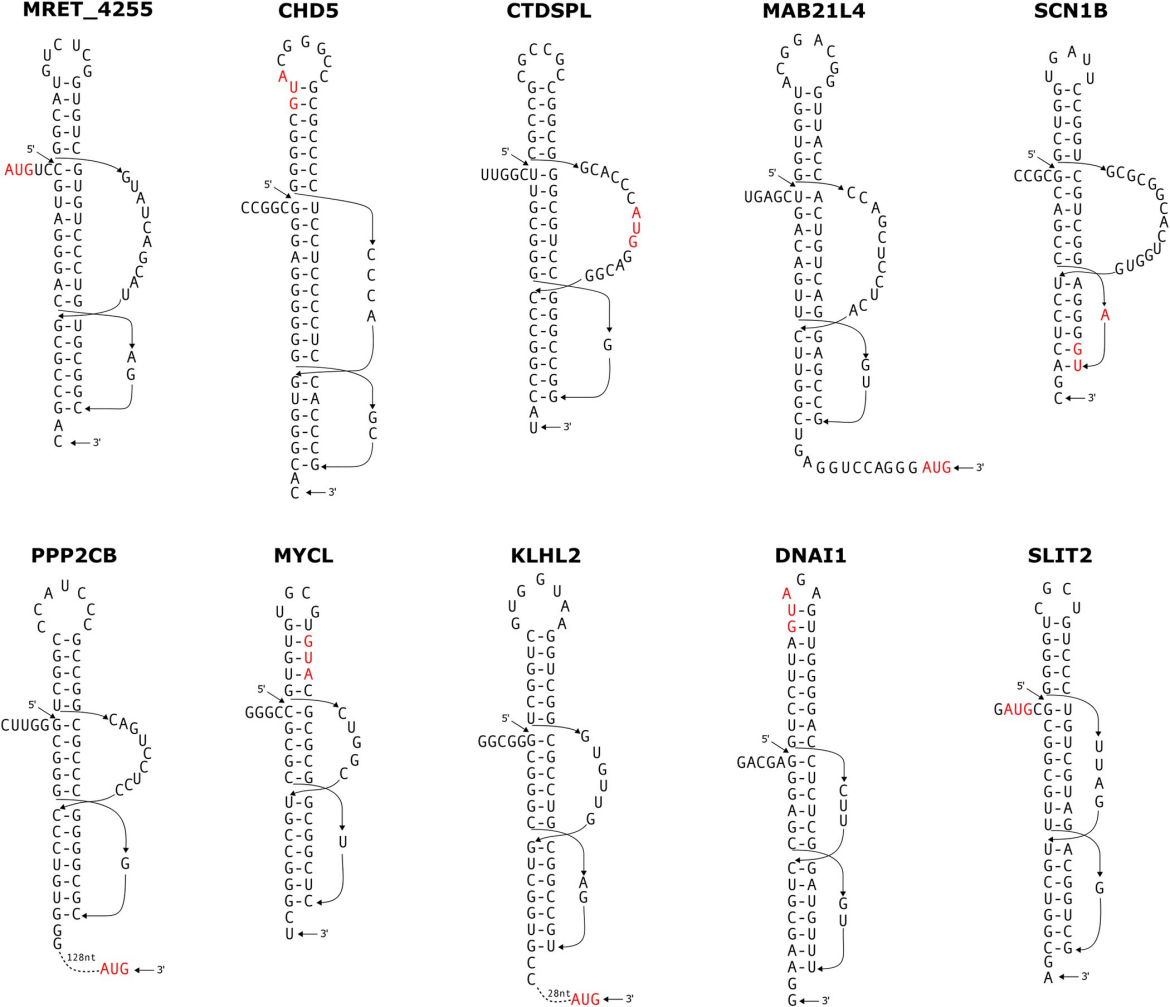
Computationally identified potential three-stemmed pseudoknots in human mRNAs, in close proximity to the AUG start codon. The AUG start codon is highlighted in red. There is no intervening sequence between the stems, therefore the three stems (S1, S2 and S3) can potentially stack to form a quasi-continuous helix.

**Fig 3:**
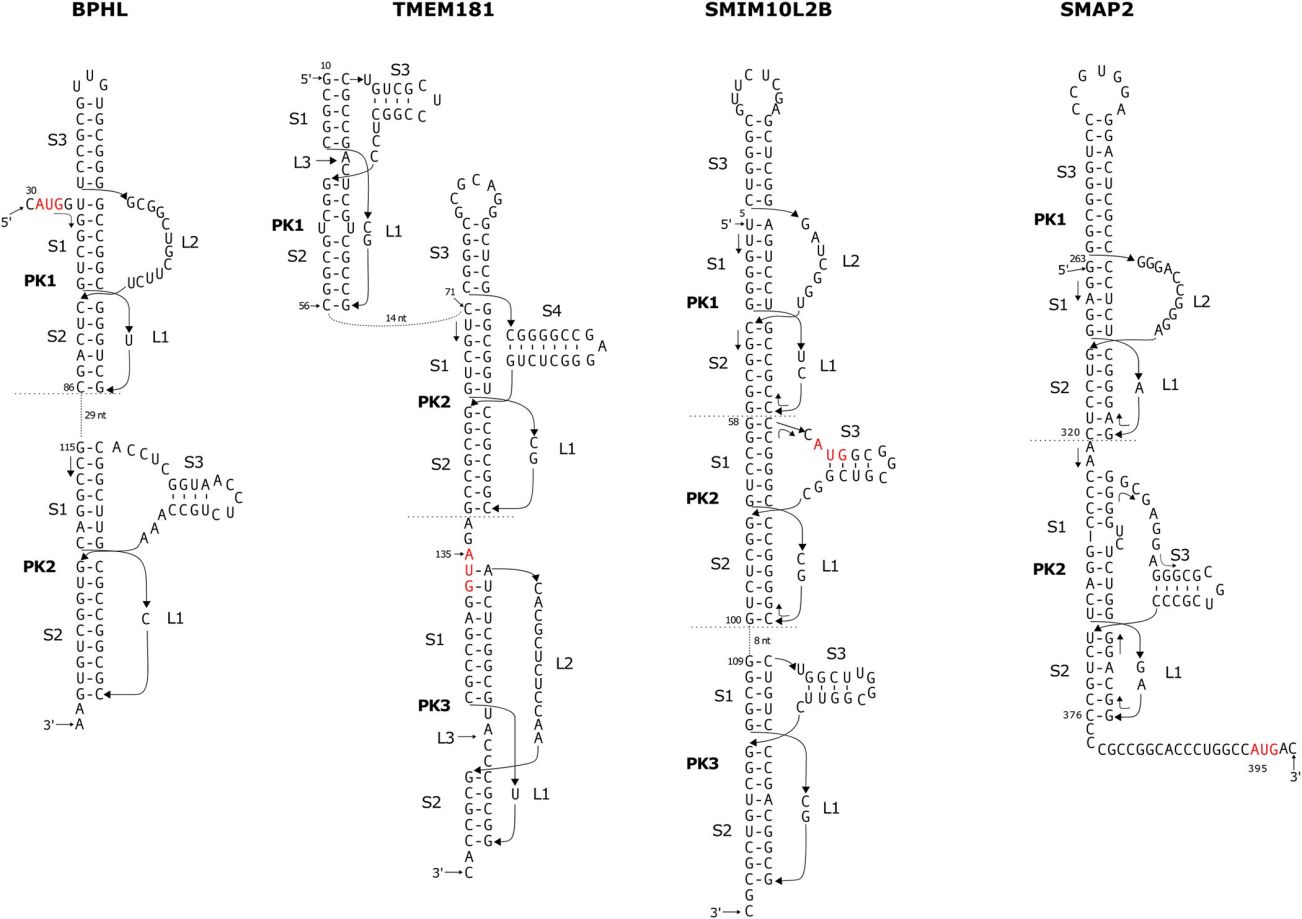
Computationally identified potential multiple pseudoknots, including at least one three-stemmed pseudoknots, in the start codon region within human mRNAs. The AUG start codon is highlighted in red. In the PK1 pseudoknots within the BPHL, SMIM10L2B and SMAP2 mRNAs, as well as the PK2 pseudoknot within the TMEM181 mRNA, there is no intervening sequence between the stems, therefore the three stems (S1, S2 and S3) can potentially stack to form a quasi-continuous helix.

Figs 2 and 3 show 14 putative cases of three-stemmed pseudoknots. These pseudoknots are found in the vicinity of the start codon region of the following human mRNAs: Malassezia restricta dynactin 4 (MRET_4255), chromodomain helicase DNA binding protein 5 (CHD5), CTD small phosphatase like (CTDSPL), mab-21 like 4 (MAB21L4), sodium voltage-gated channel beta subunit 1 (SCN1B), protein phosphatase 2 catalytic subunit beta (PPP2CB), MYCL proto-oncogene bHLH transcription factor (MYCL), kelch like family member 2 (KLHL2), dynein axonemal intermediate chain 1 (DNAI1), slit guidance ligand 2 (SLIT2), biphenyl hydrolase like (BPHL), transmembrane protein 181 (TMEM181), small integral membrane protein 10 like 2B (SMIM10L2B), and small ArfGAP2 (SMAP2). Each of these pseudoknots possesses an additional stem (stem3), which has the capacity to stack atop stem1. In the case of the TMEM181 pseudoknot, there is an additional stem, referred to as "stem4", that could potentially form within the loop2 sequence. In the instances of BPHL, TMEM181, SMIM10L2B, and SMAP2, the mRNA sequences contain a cluster of two or three potential three stemmed pseudoknots in the start codon region. Each of these potential cases of three-stemmed pseudoknots is discussed in greater detail as follows.

### MRET_4255

The 1116 nt mRNA sequence contains only the coding sequence without 5’ and 3’-UTRs. The detected three-stemmed pseudoknot locates immediately downstream from the AUG start codon (with a mere 2 nucleotide gap) (Fig 2).

The detected three-stemmed pseudoknot in MRET_4255 mRNA has a calculated free energy of -30.3 kJ/mol. It is ranked as the most stable pseudoknots within the mRNA sequence. The possible co-axial stacking of the additional stem3 onto the two mandatory stems is expected to further bolster the stability of this three-stemmed pseudoknot. There are actually only two detected potential pseudoknots within the mRNA sequence that fit the particular search criteria. The three-stemmed pseudoknot shown in Fig 2 spans nucleotides 6–63. The other potential pseudoknot, with a calculated free energy of -21.8 kJ/mol, spans nucleotides 26–84 (S1 File). Because the sequences forming the two potential pseudoknots are overlapping, the two pseudoknots cannot form simultaneously.

BLASTN analysis identified just two mRNA sequences in the databases that exhibit sequence similarity, and both of these sequences match precisely with the pseudoknot-forming region found in the MRET_4255 mRNA.

### CHD5

CHD5 serves as a chromatin remodeling protein that potentially plays a role in the formation of a nucleosome remodeling and deacetylation complex, contributing to the regulation of gene transcription. In various contexts, CHD5 is implicated as a potential tumor suppressor, in neuroblastomas, gliomas, and many common adult tumors [65–67].

The detected three-stemmed pseudoknot has a calculated free energy of -41.1 kJ/mol, and is ranked as the most stable pseudoknot among all 16 detected pseudoknots within the 9850 nt mRNA (S1 File). The stems, including the extra stem3, are rich in G-C basepairs (Fig 2). The AUG start codon is located inside the pseudoknot, within the extra stem-loop. Compared to other detected three-stemmed pseudoknots shown in Figs 2 and 3, the CHD5 three-stemmed pseudoknot features the shortest connecting loop between stem3 and stem2, which comprises a mere four nucleotides with the sequence 5’-CCCA-3’.

**Fig 4:**
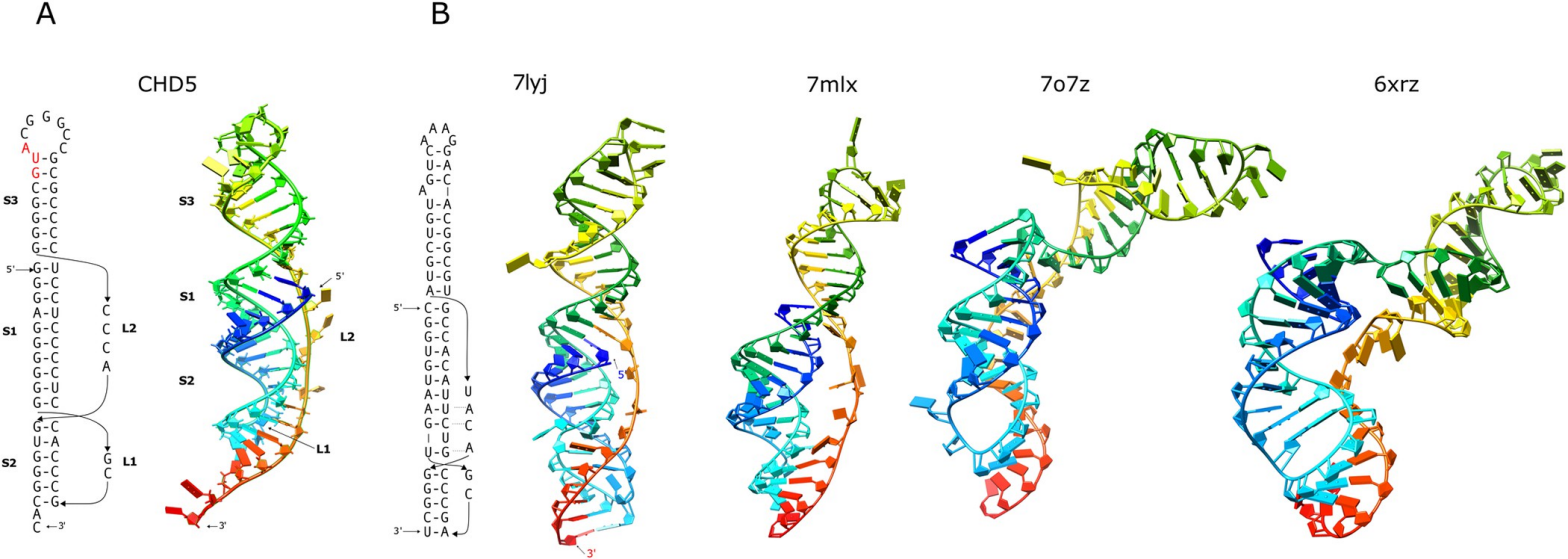
A) The three-stemmed pseudoknot in the CHD5 mRNA. The secondary structure and corresponding modeled three-dimensional structure are shown. The AUG start codon is heighted in red in the secondary structure. B) Structures of the −1 PRF stimulating three-stemmed pseudoknot in SARS-CoV-2 determined by X-ray crystallography (PDB code 7lyj and 7mlx) and cryoEM (PDB code 7o7z and 6xrz). The secondary structure revealed by 7lyj is also shown. Note that the apical loops in the two crystal structures are artificial. The structures show different base-paring schemes at the junction regions, which are also different from the originally proposed base-paring scheme as shown in Fig 1. The structures are color-ramped from 5’ in blue to 3’ in red.

In order to assess the feasibility of the base-pairing arrangement of the CHD5 three-stemmed pseudoknot illustrated in Fig 4A, assuming that the three stems are aligned coaxially to create a quasi-continuous A-form helix, a structural model for the three-stemmed pseudoknot was constructed. As shown in Fig 4A, coaxial stacking of three stems results in the formation of a quasi-continuous A-form helix totaling 23 base pairs. The 5’-CCCA-3’ connecting loop adopts a fully extended conformation, effectively spanning the ten base pairs of stem1.

The structures of the three-stemmed pseudoknot of SARS-CoV-2 were recently determined by X-ray crystallography [35, 36] and Cryo-EM [37, 38]. The structures confirm the overall three-stemmed structure of the pseudoknot. Interestingly, the base-paring schemes of the pseudoknot reported by the structural studies are slightly different from each other, and from the originally proposed scheme (comparing the scheme revealed by the crystal structure 7lyj shown in Fig 4B left panel to that shown in Fig 1). Among these structural studies, the cryoEM structure by Bhatt and coworkers (PDB code 7o7z) [37] was determined in the context of a viral RNA bound to a translating mammalian ribosome (only structure of the pseudoknot is shown in Fig 4B). The pseudoknot structure lodges at the entrance of the mRNA channel. Stem1 and Stem2 (S1 and S2) of the pseudoknot coaxially stack on top of each other to form a quasi-continuous helix, with Stem3 (S3) standing out almost perpendicular to the Stem1-Stem2 helix, forming an L-shape structure. The pseudoknot interacts directly with the proteins of the small subunit located at the mRNA entry channel, thereby creating a mechanical obstacle against unwinding by the ribosome’s intrinsic helicase activity. The other cryoEM structure, determined for a 28 kDa RNA construct, by Zhang el. al. (PDB code 6XRZ) also shows an L-shape structure (Fig 4B) [38]. The two crystal structures were determined for RNA constructs with different mutations on the apical loop, one construct replacing the natural loop sequence with a GAAA stable tetraloop (PDB code 7lyj) [36], the other construct with a artificial loop sequence serving as a binding site for an RNA chaperone protein to facilitate crystallization of the pseudoknot as a RNA-protein complex (PDB code 7mlx) [35]. The two structures reveal difference in the base-paring at the junction regions, the overall shapes of the structures also are different (Fig 4B). The structure by Jones and coworkers (PDB code 7lyj) shows coaxial stacking of the three stems to form a quasi-continuous helix even with a single-stranded nucleotide in the junction of stem1 and stem2 [36]. These structural results demonstrate the potential of the RNA pseudoknot to adopt different basepairing schemes and structures under different conditions, and undergo conformational changes upon encountering the translating ribosome.

Upon comparing the modeled structure of the CHD5 three-stemmed pseudoknot to the crystal structure of the SARS-CoV-2 three-stemmed pseudoknot with a quasi-continuous helix (7lyj), a clear overall structural similarity emerges. This observation raises legitimate concerns regarding the potential unintended consequences of anti-viral agents designed to target the viral pseudoknot. There is a possibility that such agents might inadvertently target similar structures in human mRNAs. This issue warrants careful consideration in light of the recent findings presented herein.

Performing a BLASTN search with the CHD5 three-stemmed pseudoknot sequence as the query revealed 59 mRNA sequences within the databases that share sequence similarities. All of these mRNA sequences correspond to primate CHD5 proteins. Although certain sequence differences are observed in some of the sequences, all of them retain the ability to form a three-stemmed pseudoknot, as indicated by S1 File.

### CTDSPL

The analysis identified a three-stemmed pseudoknot with a computed free energy of -38.9 kJ/mol, which stands out as the top-ranked among all 5 detected potential pseudoknots in the 4753 nucleotide mRNA sequence. The three stems predominantly consist of G-C base pairs, counting for 16 out of a total of 19 base pairs (as illustrated in Fig 2). Notably, the AUG start codon resides within the connecting loop that links stem3 and stem2. In comparison to other three-stemmed pseudoknots shown in Fig 2, the CTDSPL pseudoknot exhibits the second most lengthiest connecting loop between stem3 and stem2, comprising 14 nucleotides.

Interestingly, it is noted that the second-ranked pseudoknot (with a computed free energy of -30.2 kJ/mol) also has the potential to form an extra three-base pair stem (not shown). This pseudoknot-forming sequence is situated within the 5’-UTR of the mRNA, positioned upstream from the top-ranked pseudoknot. The top-ranked pseudoknot spans nucleotides 275 to 335, whereas the second-ranked pseudoknot encompasses nucleotides 74 to 118.

The potential CTDSPL three-stemmed pseudoknot comprises a 14-nucleotide loop linking stem3 and stem2, crossing over stem1, which contains 7 base pairs. While it is unquestionable that the loop should be long enough to facilitate coaxial stacking of the three stems, we believe it is worthwhile to undertake a model-building study on this pseudoknot. This study aims to explore how a three-stemmed pseudoknot with a relatively long connecting loop may manifest the loop within the three-dimensional structure.

**Fig 5:**
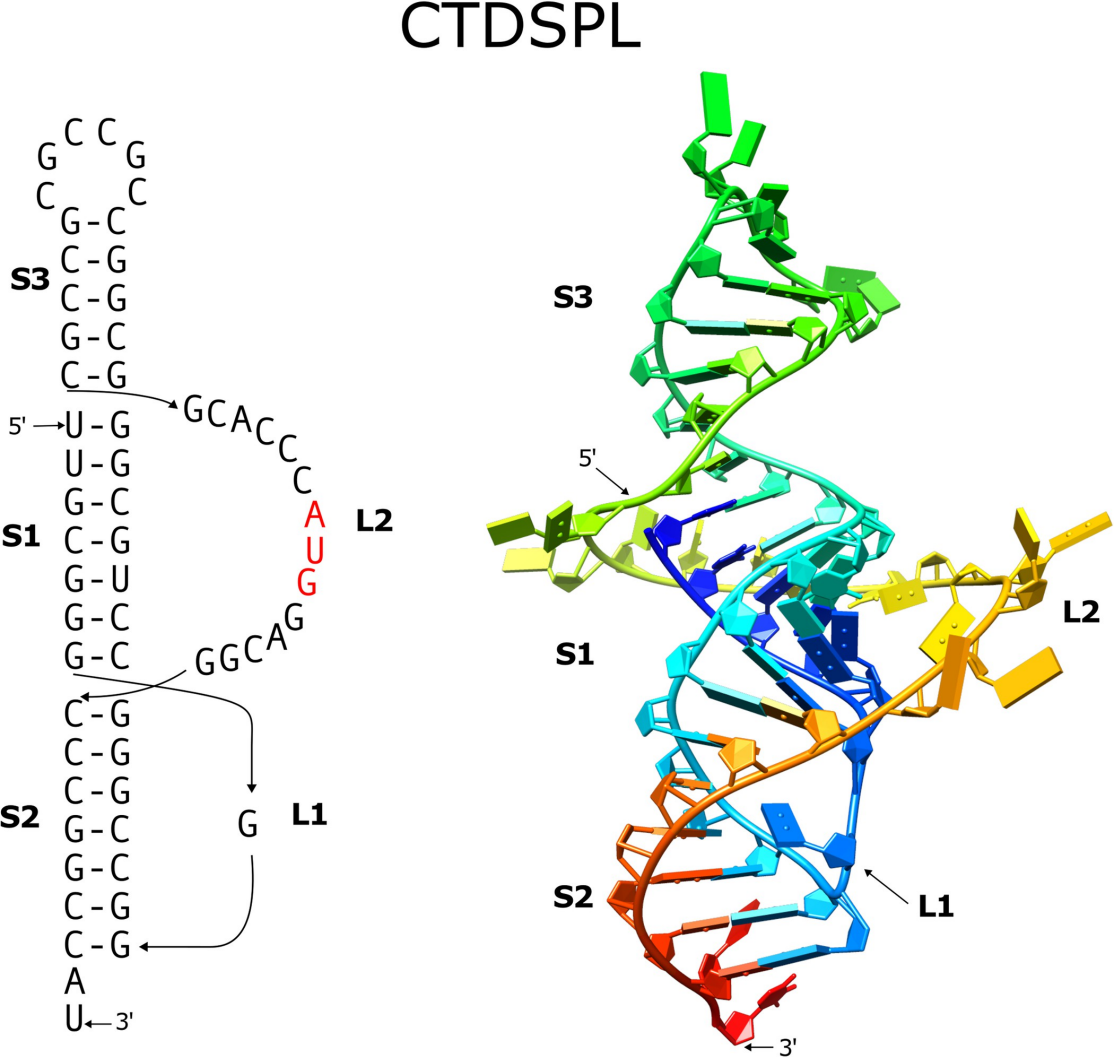
The three-stemmed pseudoknot in the CTDSPL mRNA. The secondary structure and corresponding modeled 3-dimensional structure are shown. The AUG start codon is heighted in red in the secondary structure. The modeled structure is rendered in cartoon mode with color-ramping from blue at the 5′-end to red at the 3′-end.

In the modeled structure, the three stems are stacked coaxially, forming a quasi-continuous A-form helix comprised of 19 base pairs (Fig 5). The 14-nucleotide connecting loop winds around this helix, following the minor groove’s general path, serving as a bridge between stem3 and stem2. This conformation showcases a seamless transition of the RNA backbone at the junctions between stem3 to the loop and the loop to stem2. It’s worth noting that the loop nucleotides, including those within the AUG start codon, have the potential to engage in minor groove interactions, although no specific constraints were imposed during the model construction process to enforce such interactions. It’s important to emphasize that the model structure presents one possible and reasonable configuration, recognizing that alternative structures are feasible. The objective of this modeling study is not to exhaustively explore the entire conformational space of the pseudoknot.

BLASTN analysis reveals 238 hits, all of which originate from CTDSPL mRNA sequences across various species. Notably, all these homologous sequences maintain the capacity to form a three-stemmed pseudoknot, as corroborated by support information (S2 File).

### MAB21L4

In the MAB21L4 mRNA, a three-stemmed pseudoknot with a calculated free energy of -27.9 kJ/mol is identified. This pseudoknot holds the second position in terms of lowest calculated free energy among all 7 pseudoknots found within the 2015 nucleotide mRNA sequence. The top-ranked pseudoknot, with a calculated free energy of -29.3 kJ/mol, is situated within the coding region. It’s worth considering that when accounting for the possible coaxial stacking with the additional stem, the three-stemmed pseudoknot may exhibit even greater stability. Notably, the AUG start codon (nucleotides 97–99) is positioned 12 nucleotides downstream from the predicted three-stemmed pseudoknot (spanning nucleotides 24–84), as depicted in Fig 2. Furthermore, it’s interesting to mention that an upstream in-frame stop codon, UGA, is immediately found downstream from the pseudoknot without any intervening gaps. The 23 nucleotides upstream of the pseudoknots have the potential to fold into a relatively weak stem-loop, as predicted by RNAfold (not shown). The predicted three-stemmed pseudoknots spans about 64% nucleotides in the 5’UTR, representing the dominant structure within the 5’UTR.

Performing a BLASTN search with the MAB21L4 three-stemmed pseudoknot sequence as the query returns 13 matches, all of which are associated with MAB21L4 mRNA sequences found in primates. Among these 13 sequences, 7 display identical matches, while the remaining 6 sequences maintain the capability to form the three-stemmed pseudoknot structure, albeit with some sequence variations (S2 File).

### SCN1B

The detected three-stemmed pseudoknot in SCN1B mRNA has a calculated free energy of -27.8 kJ/mol. This pseudoknot ranks as the most stable among all 5 potential pseudoknots identified within the 1666 nt mRNA sequence. The pseudoknot encompasses the AUG start codon, situated in the loop1 and stem2 regions, as depicted in Fig 2.

A BLASTN search was conducted using the identified three-stemmed pseudoknot sequence from SCN1B as the query. This search yielded 36 matches, all from primates, with 32 of these sequences being identical to the human SCN1B mRNA sequence. The remaining four sequences display slight variations, differing by 1 or 2 positions compared to the query. Notably, all of these other sequences have the potential to adopt the three-stemmed pseudoknot structure (S2 File).

### PPP2CB

The detected three-stemmed pseudoknot in PPP2CB mRNA has a calculated free energy of -29.3 kJ/mol. It is ranked as the most stable pseudoknots among all the 3 potential pseudoknots identified within the 1946 nt mRNA sequence. The AUG start codon is located 121 nt downstream from the pseudoknot.

Performing a BLASTN search using the identified three-stemmed pseudoknot sequence from PPP2CB as the query has resulted in 12 matches. Each of these sequences have the potential to form a three-stemmed pseudoknot structure (S1, S2 Files).

### MYCL

The detected three-stemmed pseudoknot in MYCL mRNA has a calculated free energy of -27.4 kJ/mol. This pseudoknot ranks as the most stable among all 4 potential pseudoknots identified within the 1942 nt mRNA sequence. The pseudoknot encompasses the AUG start codon, located within the additional stem3 (Fig 2). The 5’UTR is relatively short. There are only 10 nucleotides (purine-rich) upstream from the predicted pseudoknot, which does not have the potential to form a stable structure. Therefore The predicted three-stemmed pseudoknot may represent the sole significant structure in the 5’UTR and starting codon region of the mRNA.

A BLASTN search was conducted using the identified three-stemmed pseudoknot sequence from MYCL as the query. This search yielded 30 matches, all from primates. The sequence are highly conserved, with 8 of these sequences being identical to the human MYCL mRNA sequence. The remaining 22 sequences display only one or two nucleotides difference in loop 2 (S1, S2 Files).

### KLHL2

In the KLHL2 mRNA, the detected three stemmed pseudoknot, with a calculated free energy of -26.1 kJ/mol, is the first ranked potential pseudoknot identified within the 3185-nucleotide mRNA sequence. The AUG start codon is located 28 nucleotides downstream from this predicted three-stemmed pseudoknot (Fig 2). Additionally, it’s worth noting that an upstream in-frame stop codon, UGA, can be found just 7 nucleotides downstream from the pseudoknot. This configuration, where an upstream in-frame stop codon precedes the start codon in the sequence downstream from the pseudoknot, is a feature shared by both the MAB21L4 and KLHL2 mRNAs.

There are only two potential pseudoknots identified within the mRNA that meet the search criteria. The other identified pseudoknot (not shown), with a calculated free energy of -23.0 kJ/mol, is located within the coding region. It is also a potential three-stemmed pseudoknot with three basepairs in the extra stem (S1, S2 Files).

Conducting a BLASTN search with the KLHL2 three-stemmed pseudoknot sequence as the query has yielded 237 matches, encompassing KLHL2 mRNAs from a wide range of species. Importantly, all of these sequences exhibit the potential to form a three-stemmed pseudoknot structure (S2 File).

### DNAI1

The detected three-stemmed pseudoknot in DNAI1 mRNA has a calculated free energy of -23.3 kJ/mol. This pseudoknot is the first ranked pseudoknot identified within the 2529 nt mRNA sequence. The pseudoknot encompasses the AUG start codon, situated within the additional stem-loop, as depicted in Fig 2.

Similar to the KLHL2 mRNA, there are only two potential pseudoknots identified within the DNAI1 mRNA that meet the search criteria. The other identified pseudoknot (not shown), with a calculated free energy of -22.0 kJ/mol, is located within the coding region.

A BLASTN search was conducted using the identified three-stemmed pseudoknot sequence from DNAI1 as the query. This search yielded 58 matches, all from primates, with 16 of these sequences being identical to the human DNAI1 mRNA sequence. The remaining 42 sequences display slight variations, differing by 1 position compared to the query, which leads to a mismatch pair in the 9-basepair stem3. All of these other sequences have the potential to adopt the three-stemmed pseudoknot structure (S1, S2 Files).

### SLIT2

The detected three-stemmed pseudoknot in SLIT2 mRNA has a calculated free energy of -28.6 kJ/mol. This pseudoknot is the first ranked potential pseudoknot identified within the 8053 nt mRNA sequence. The pseudoknot situates just one nucleotide downstream from the AUG start codon (Fig 2). There are only two potential pseudoknots identified within the SLIT2 mRNA that meet the search criteria. The other identified pseudoknot (not shown), with a calculated free energy of -22.1 kJ/mol, is located within the coding region.

A BLASTN search was conducted using the identified three-stemmed pseudoknot sequence from SLIT2 as the query. This search yielded 257 matches, across a wide range of species, with 83 of these sequences being identical to the human SLIT2 mRNA sequence. The remaining sequences display slight variations, differing by 1 to 5 positions compared to the query. Importantly, most of the variations maintain basepairing interactions. Even when the variations disrupt basepairing interaction, only one mismatch is resulted in stem1 or stem2. In some sequences, the variations even lead to stronger stems due to G-U pair to G-C pair changes. All of these other sequences have the potential to adopt the three-stemmed pseudoknot structure (S1, S2 Files).

### BPHL

The detected three-stemmed pseudoknot in BPHL mRNA has a calculated free energy of -26.3 kJ/mol. This pseudoknot ranks as the most stable among all 3 potential pseudoknots identified within the 1909 nt mRNA sequence. The pseudoknot situates just one nucleotide downstream from the AUG start codon, as depicted in Fig 3. The 5’UTR of this mRNA contains only 30 nucleotides. Secondary structure prediction by RNAfold indicates that the 5’UTR does not have the potential to form significant helical stems. The predicted three-stemmed pseudoknot may therefore represent the first significant structure in the mRNA.

To test whether the sequences flanking the detected three-stemmed pseudoknot also have the potential to harbor a pseudoknot structure, we used PKscan with more generous settings for the stem and loop ranges to search the upstream and downstream sequences. A potential pseudoknot was identified shortly downstream (Fig 3, PK2). This pseudoknot, with a calculated free energy of -34.3 kJ/mol, has even stronger stem1 and stem2 compared to the three-stemmed pseudoknot PK1. The loop2 sequence of PK2 also has the potential to form an extra stem with four basepairs. To be noted, loop1 of PK2 has only one nucleotide, which might not be able to cross the major groove of stem2 with 9 basepairs. It is possible that the terminal basepair does not form and loop1 increases to two nucleotides.

A BLASTN search was conducted using the identified three-stemmed pseudoknot sequence from BPHL as the query. This search yielded 39 matches, all from primates. The sequence are extremely conserved, with 15 of these sequences being identical to the human BPHL mRNA sequence. The remaining 24 sequences display only one nucleotide difference in loop 2 (S1, S2 Files).

### TMEM181

The identified three-stemmed pseudoknot in the TMEM181 mRNA, with a calculated free energy of -29.2 kJ/mol, is the first ranked detected pseudoknots within the 5103 nucleotide mRNA sequence (Fig 3, PK2, starting at nucleotide 71). The three stems are also mainly comprised of G-C basepairs, with 14 out of 18 basepairs being G-C basepair. The AUG start codon is positioned immediately downstream from the predicted three-stemmed pseudoknot (Fig 3). The only other detected pseudoknot is located in the 3’UTR (not shown).

Compared to the other three-stemmed pseudoknots depicted in Figs 2 and 3, the TMEM181 three-stemmed pseudoknot stands out with the longest connecting loop between stem3 and stem2, spanning a total of 17 nucleotides. The loop sequence has the capability to form a helical stems (referred to as stem4) consisting of 7 base pairs, capped by a 3-nucleotide apical loop.

We attempted to construct a model structure adhering to the base-pairing scheme depicted in Fig 3, while also maintaining coaxial stacking of the three stems 1–3. However, it became apparent that under these constraints, constructing a physically plausible four-stemmed pseudoknot structure was impossible. This necessitated the disruption of some base pairs in stem4 and/or the non-coaxial stacking of stem3 on stem1 to allow for the creation of a four-stemmed pseudoknot. Alternatively, stem4 might not form at all. Regardless of these variations, the fundamental three-stemmed pseudoknot configuration remained feasible.

Using PKscan with more generous settings for the stem and loop ranges to search the upstream and downstream sequences, we identified a potential upstream pseudoknot (PK1) spanning nucleotides 10–56, and a downstream pseudoknot (PK3) with only three intervening nucleotides from PK2 (Fig 3). Both PK1 and PK3 have a loop3 (L3) between stems S1 and S2. The loop2 of PK1 has the potential to harbor an extra stem with four basepairs. PK1 is also a three-stemmed pseudoknot. This three-stemmed pseudoknot is topologically similar to the three-stemmed riboswitch pseudoknots shown in Fig 1, having an intervening sequence connecting the extra stem to stem1 or stem2.

The 5’UTR of the TMEM181 mRNA has 134 nucleotides. PK1 and PK2 cover more that 82% of the 5’UTR, leaving only 10 nucleotides at the 5’-end and 14 nucleotides between the two pseudoknots. PK3 covers the start codon and more than 40 nucleotides downstream. The three potential pseudoknots therefore represent the dominant structures within the 5’UTR and the start codon regions of the TMEM181 mRNA.

Conducting a BLASTN search using the TMEM181 three-stemmed pseudoknot sequence as the query yields 25 matches, all of which correspond to TMEM181 mRNA sequences from primates. Among these 25 sequences, 14 exhibit complete identity, while the remaining 11 sequences have the capacity to adopt the anticipated structural arrangement. The majority of variations observed in these 11 sequences involve substitutions such as U-G to C-G and G-U to A-U in stem1, as well as C-G to U-G in stem2, as detailed in the support information (S1, S2 Files).

### SMIM10L2B

The top three ranked pseudoknots among the nine potential pseudoknots detected in the 2817 nucleotide SMIM10L2B mRNA have a calculated free energy of -32.8, -32.1 and -28.5 kJ/mol respectively. The 3^rd^ ranked pseudoknot has the potential to contain an extra stem with 6 basepairs (4 G-C and 2 G-U basepairs) (Fig 3, designated as PK1). Accounting for the possible coaxial stacking with this additional stem, the three-stemmed pseudoknot may have greater stability.

Interestingly, the first-ranked pseudoknot (Fig 3, designated as PK2) resides immediately downstream of the three-stemmed pseudoknot PK1. There is no intervening gap sequence between these two pseudoknots, allowing them to arrange in tandem. Consequently, the five stem regions derived from these two pseudoknots have the potential to stack coaxially, forming a quasi-continuous helix encompassing a total of 31 base pairs, with 22 of them being G-C base pairs. Additionally, the loop2 sequence of the downstream pseudoknot has the potential to contain an extra stem, making it also a three-stemmed pseudoknot. The AUG start codon locates within this loop2 sequence (Fig 3).

The tandemly arranged pseudoknots PK1 and PK2 starts at the 5^th^ nucleotide and ends 18 nt after the AUG start codon, covering the entire 5’UTR and start codon regions of the mRNA.

**Fig 6:**
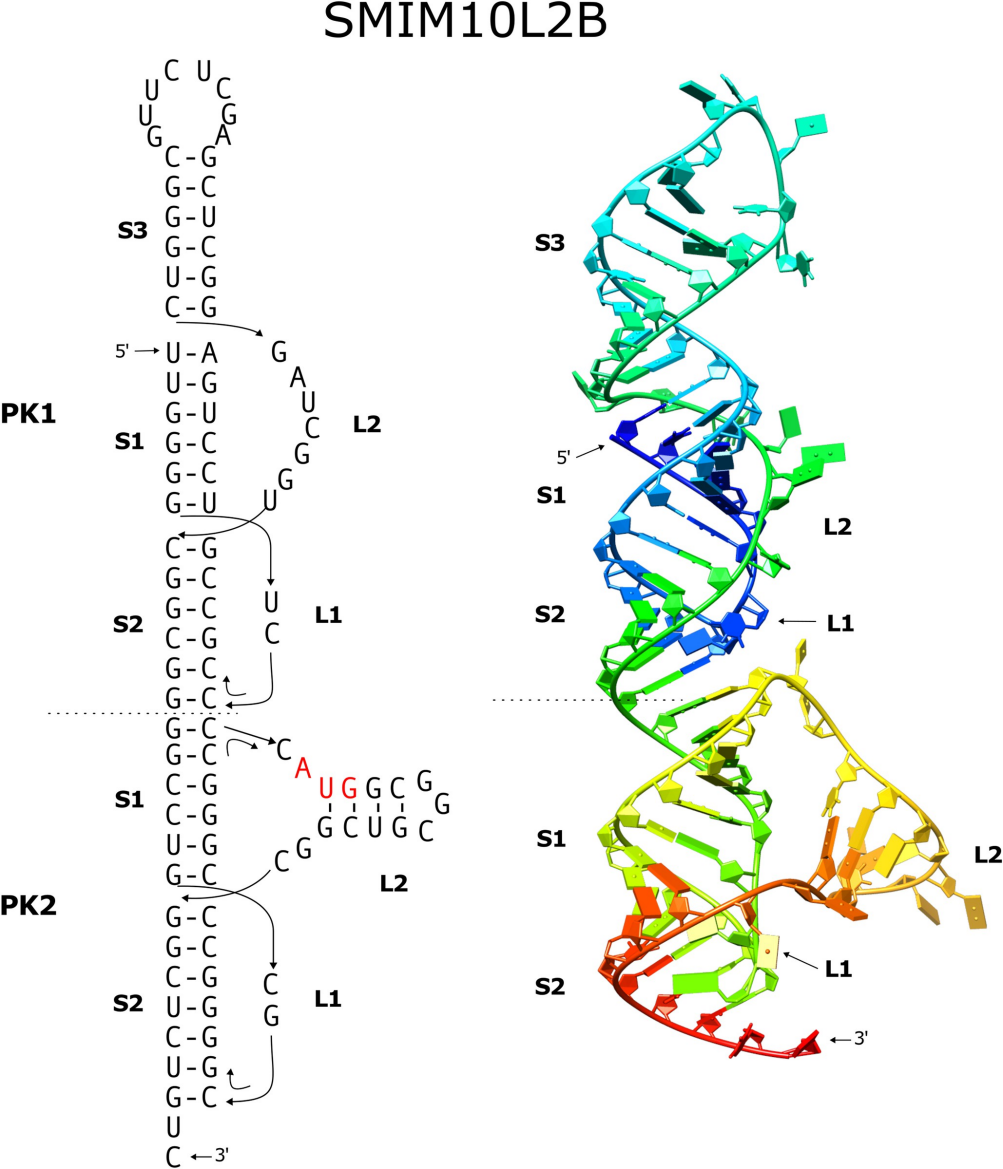
The tandem three-stemmed pseudoknots in the SMIM10L2B mRNA. The secondary structure and corresponding modeled three-dimensional structure are shown. The AUG start codon is heighted in red in the secondary structure. The modeled structure is rendered in cartoon mode with color-ramping from blue at the 5′-end to red at the 3′-end.

We constructed a structural model to elucidate the tandem pseudoknots PK1 and PK2 identified within the SMIM10L2B mRNA, as depicted in Fig 6. Our modeling results confirm the RNA’s ability to adopt the predicted base-pairing configuration while maintaining the coaxial alignment of its five stems. The resulting structure exhibits an elongated yet relatively compact form.

Given that both pseudoknots belong to the CPK1 family, it’s noteworthy that the two nucleotides in loop1 of each pseudoknot position their bases within the major groove of stem1. Additionally, loop2 of the upstream three-stemmed pseudoknot PK1, which links stem3 and stem2, wraps around the helix of stem1, tracing the minor groove. It’s worth mentioning that this loop can also adopt alternative conformations, including a more extended one that roughly parallels the helix axis.

In the downstream pseudoknot PK2, loop2 harbors an additional stem-loop with four base pairs within the stem. This stem-loop arrangement is approximately perpendicular to the helix of stem2. As a result, this pseudoknot can also be classified as a three-stemmed pseudoknot. The primary distinction between the two three-stemmed pseudoknots lies in their structural arrangement. In the upstream three-stemmed pseudoknot PK1, stem3 directly stacks onto stem1. Conversely, in the downstream three-stemmed pseudoknot PK2, an intervening sequence separates stem1 from the additional stem3. This leads to stem3 being positioned perpendicular to the main helix.

It was also found that the downstream sequence from PK2 also has the potential to form a pseudoknot, identified by using PKscan with more generous settings for the stem and loop ranges. This pseudoknot (Fig 3, PK3) is located 8 nucleotides downstream from PK2. The loop2 sequence of PK3 also has the potential to harbor an extra stem with four basepairs, leaving one single-stranded nucleotide connected to stem1 and stem2.

For the sequence forming the three-stemmed pseudoknot PK1, BLASTN analysis unveiled a limited pool of 9 homologous mRNA sequences, exclusively originating from primates. Among these, 5 sequences exhibit a 100% identity, while the remaining 4 sequences display variations of up to 4 nucleotides. Importantly, all of these sequences possess the capacity to adopt similar structural configurations (as detailed in the S1, S2 Files).

### SMAP2

The detected three-stemmed pseudoknot in SMAP2 mRNA (Fig 3, the upstream pseudoknot PK1) has a calculated free energy of -24.0 kJ/mol. It is ranked as the 2^nd^ most stable pseudoknots among the four potential pseudoknots identified within the 2905-nt mRNA sequence. This pseudoknot resides within the 5’-UTR of the mRNAs, with the AUG start codon locating 72 nt downstream. The top ranked pseudoknot, with a calculated free energy of -25.6 kJ/mol, also have the potential to assume a three-stemmed configuration (not shown). It is located within the coding region. The extra stem in the three-stemmed pseudoknot PK1 shown in Fig 3 has 9 basepairs (mostly G-C), making it the longest coaxially stacked extra stem among all the three-stemmed pseudoknots shown in Figs 2 and 3.

Interestingly, the 3^rd^ ranked identified pseudoknot (Fig 3, PK2), with a calculated free energy of -22.1 kJ/mol, is located between the three-stemmed pseudoknot PK1 and the AUG start codon. The originally identified pseudoknot by PKscan has five basepairs in stem1. This stem can be extended to include a two-nucleotide bulge and four more G-C basepairs. The loop2 sequence of PK2 also has the potential to form an extra stem3, with intervening sequences connected to the other two stems (Fig 3).

As depicted in Fig 3, there are two unpaired adenine nucleotides separating the two three-stemmed pseudoknots PK1 and PK2. Given the importance of base-stacking interactions in stabilizing RNA folding, it is entirely conceivable that these two nucleotides could form two mismatched A-G and A-C pairs atop the predicted stem 1 of the downstream pseudoknot PK2, which would facilitate continuous stacking of the stems in both pseudoknots. It is not uncommon for mismatched pair to form at helical junctions. For example, structure of the preQ1–II riboswitch pseudoknot shows that an A-G mismatched pair is formed at the end of stem1 at the stem1 and stem2 junction, enabling coaxial stacking of the two stems (Fig 1) [40].

Performing a BLASTN search with the identified three-stemmed pseudoknot PK1 sequence from SMAP2 as the query has resulted in 121 matches, encompassing SMAP2 mRNA sequences across a diverse array of species. Each of these sequences possesses the capacity to form a three-stemmed pseudoknot structure (S1, S2 Files).

## Discussion

In this study, we aim to identify occurrences of a particular type of three-stemmed pseudoknots in human mRNA. These pseudoknots contain three stems without any sequence interruptions between them. Similar features have been reported for several viral pseudoknots known to induce −1 programmed ribosomal frameshifting (−1 PRF) (Fig 1) [22, 32, 34, 41]. Our search covered the full-length mRNA sequences, with no assumptions made regarding the specific location and biological function of the pseudoknots. Potential three-stemmed pseudoknots were identified in various regions of the mRNAs, including the 5’-UTR, coding region, and 3’-UTR.

This paper presents fourteen instances of the identified potential three-stemmed pseudoknots located in close proximity to the AUG start codon. The start codon is immediately upstream of the pseudoknot, within the pseudoknot, or closely downstream from the pseudoknot. Among these identified pseudoknots, ten rank as the most stable pseudoknot identified within the full-length mRNA sequences, three rank second, and the remaining one ranks third. The mRNA sequences vary in length, ranging from 1116 to 9850 nucleotides (nt). In this study, we employ an energy threshold of -18 kJ/mol to eliminate potential pseudoknots with relatively high free energy (lower stability). Although this threshold is generous enough to include potential pseudoknots with relatively weak stems, only several potential pseudoknots were identified in each of the full-length mRNAs, with the sole exception being the 9450-nt long CHD mRNA, in which 16 potential pseudoknots were detected.

To evaluate how the potential pseudoknots discussed in this study compare to well-documented pseudoknots in existing literature, we applied the same method for calculating free energy to a selection of previously reported and extensively studied pseudoknots. The resulting free energy values are as follows: −1 PRF stimulating pseudoknot in IBV: -35.6 kJ/mol [78]; −1 PRF stimulating pseudoknot in SRV-1: -33.7 kJ/mol [28]; −1 PRF stimulating pseudoknot in HIV-1 sub-group O: -37.9 kJ/mol [32]; −1 PRF stimulating pseudoknot in SARS-CoV: -28.7 kJ/mol [19, 22, 34]; Stop codon read-through stimulating pseudoknot in Mo-MuLV: -40.9 kJ/mol [44]; Bacteriophage T2/T6 autoregulatory pseudoknot in gene 32 mRNA: -25.2 kJ/mol [69, 79]. Notably, the stability of the detected pseudoknot presented in this paper, excluding the contribution from the extra stem, is comparable to that of established functional pseudoknots. Additionally, it is anticipated that possible coaxial stacking of the extra stem will further enhance the structural stability of the three-stemmed pseudoknots.

The fact that most of the discussed pseudoknots rank at the top, even without considering the extra stem’s contribution, lends substantial credibility to their potential significance. According to the specified search criteria, every one of these identified three-stemmed pseudoknots falls within the CPK-1 pseudoknot family. Considering the prevalence of CPK-1 pseudoknots [68–70, 72], the likelihood of forming a CPK-1 pseudoknot structure is higher compared to other alternative structures. This forms the primary rationale for focusing our search exclusively on CPK-1 pseudoknots in this proof-of-concept study. CPK-1 pseudoknots, particularly those rooted in a stable stem-loop structure with 8–9 nucleotides in the loop, carry greater credibility than other structures. Additionally, the possibility of homologous sequences forming three-stemmed pseudoknots lends further support to the presence of the identified pseudoknots. It’s important to note, however, that definitively confirming the existence of these pseudoknots and understanding their biological functions will necessitate experimental investigation.

It’s also important to acknowledge the limited scope of the search carried out in this study. Firstly, the search parameters for stems and loop ranges were deliberately configured to narrow the focus solely on CPK-1 type pseudoknots. Broadening these parameters for stem and loop ranges could potentially lead to the identification of more potential pseudoknots, as illustrated by the examples of identifying some of the upstream and downstream pseudoknots shown in Fig 3. PK2 in the BPHL mRNA, PK1 and PK3 in TMEM181 mRNA, and PK3 in SMIM10L2B mRNA were all identified by PKscan with more generous ranges for the stems and loops. Secondly, the current program is tailored exclusively to identify pseudoknots characterized by perfectly matched stems, and it lacks the capability to detect pseudoknots that contain mismatched pairs or bulges within their stem regions. It’s worth noting that many well-documented pseudoknots exhibit such irregularities [22, 34, 42, 44, 78], including mismatches and bulges, as exemplified in Fig 1 with the SARS-CoV pseudoknot, which showcases bulges in both stem2 and stem3. The current version of the PKscan program recognizes these pseudoknots as if the base pairs outside the mismatch or bulge regions do not form, resulting in a reduced count of base pairs within the stems (see Fig 3 the downstream pseudoknot PK2 in SMAP2 mRNA for example). Consequently, this leads to a higher calculated free energy. If the calculated free energy surpasses the energy threshold (default -18 kJ/mol) used to filter out pseudoknots, the potential pseudoknot under consideration would be omitted from the list of detected pseudoknot candidates.

We are currently working on improving the program’s functionality by integrating the detection of pseudoknots that include mismatches and bulges. This enhancement is expected to lead to the identification of a considerably higher number of potential pseudoknot cases when accounting for these structural irregularities. A more thorough investigation will be conducted once the upgraded, more potent version of the computational method becomes available.

Previously, there have been documented instances of pseudoknot structures located in the 5’-UTR in the vicinity of the start codon. For instance, in the case of Influenza A, it was observed that a magnesium-dependent pseudoknot structure encompasses the PB1-F2 and N40 start codons [80]. In certain viruses like the Hepatitis C virus (HCV) and Bovine viral diarrhea virus (BVDV), a pseudoknot structure within the internal ribosome entry site (IRES) in the 5’-UTR near the AUG start codon plays a crucial role in IRES mediated translational initiation of the viral mRNAs [81]. To the best of our knowledge, there have been no reported instances of pseudoknots located in the vicinity of the AUG start codon in cellular mRNAs. In fact, the occurrence of pseudoknots in the 5’-UTR of eukaryotic mRNAs is exceedingly rare in scientific literature.

Only two documented cases of pseudoknots within the 5’-UTR of human mRNAs exist. The first case was identified in the L-*myc* mRNA, which features an internal ribosome entry site (IRES) in its 5’-UTR. Within the IRES, a pseudoknot was both predicted and demonstrated to play a crucial role in IRES-mediated translational initiation [82]. It’s worth noting, however, that the pseudoknots found in L-myc mRNA does not conform to the H-type pseudoknot category. Instead, it represents a long-range base-pairing interaction within larger RNA structures, falling under the broader definition of pseudoknots. In the second case, a conserved pseudoknot shared among mammals was predicted in the 5′-UTR of human interferon gamma (IFNG) mRNA [45]. This pseudoknot is positioned far upstream from the initiation codon and incorporated into a larger RNA structure, which interacts with a PKR dimer, triggering kinase activation. Once activated, PKR inhibits the translation of IFNG mRNA by phosphorylating the initiation factor eIF2alpha chain.

The three-stemmed pseudoknots presented in this study represent the first instances of their kind identified through computational analysis within human mRNAs. These pseudoknots are anticipated to be associated with the regulation of translational initiation, given their specific positioning in the vicinity of the start codon. In the cases of MYCL, BPHL, TMEM181, and SMIM10L2B mRNAs, the predicted pseudoknot or a cluster of 2–3 pseudoknots dominates the 5’UTR and start codon regions of the mRNA. In terms of their size and stability, these pseudoknots are comparable to established or hypothesized three-stemmed pseudoknots that stimulate −1 PRF or stop codon readthrough in viral mRNAs. Pseudoknots involved in frameshifting or stop codon readthrough in mRNAs are recognized for their capacity to temporarily halt ribosome progression [7, 16, 83]. It is reasonable to assume that the pseudoknots reported in this study might also possess the capability to impede the preinitiation complex (PIC). Furthermore, it is conceivable that these pseudoknots could function as binding sites for as-yet-unidentified regulatory factors. The recruitment of these factors to the region around the start codon in mRNAs might either inhibit or facilitate the initiation of translation. The presence of these pseudoknots could potentially furnish mechanisms for modulating translation efficiency, responding to various cellular conditions and the presence of specific factors. Further studies are required to elucidate the functional roles of these newly identified potential pseudoknots in human mRNAs.

**Fig 7:**
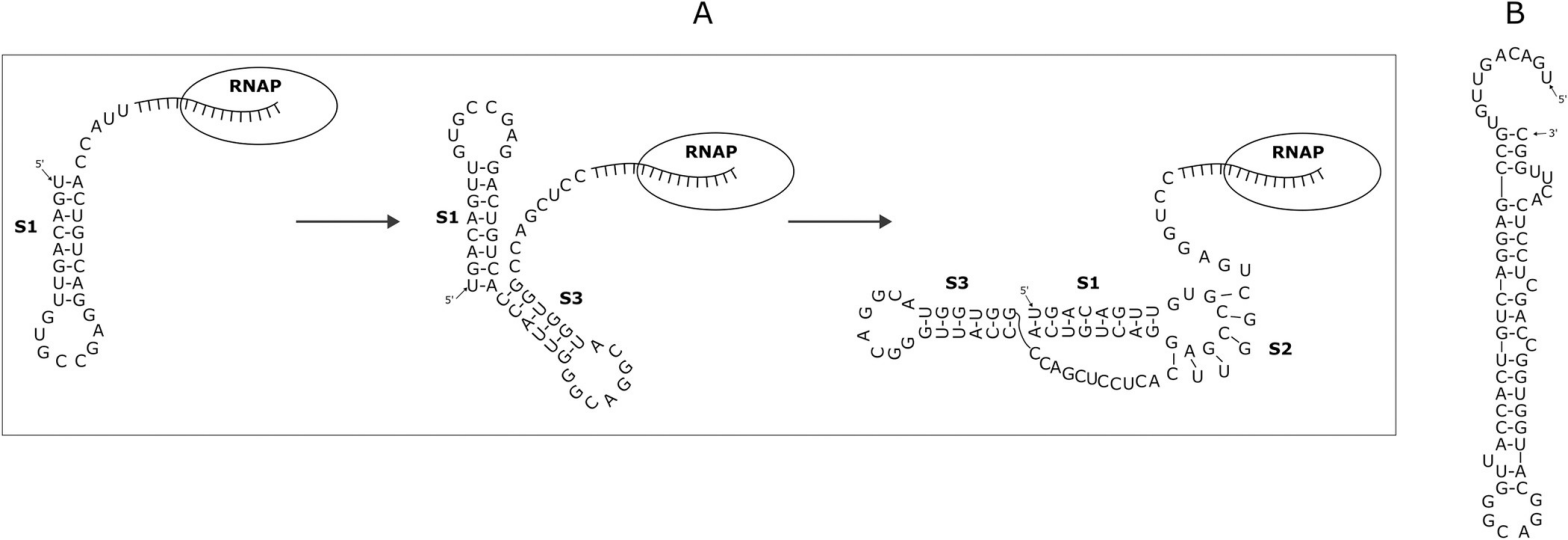
A proposed mechanism for the formation of the three-stemmed pseudoknot in the MAB21L4 mRNA. Panel A outlines the sequential formation of this pseudoknot in three distinct steps, with further details provided in the main text. In Panel B, an alternative secondary structure for the same RNA sequence is presented, as predicted by RNAfold.

During our research, we observed that some of the mRNAs containing the identified three-stemmed pseudoknots also have the potential to adopt alternative secondary structures. We made this observation by employing the RNAfold server to predict possible secondary structures for the RNA sequences [84]. It’s important to note that RNAfold does ot account for pseudoknot formation in its predictions. Nevertheless, some of the alternative secondary structures appeared quite plausible, particularly those resembling an overall stem-loop structure with an elongated stem featuring occasional mismatches and bulges (Fig 7).

Even in cases where such alternative stem-loop structures could form, we maintain our belief that the predicted three-stemmed pseudoknots are more likely to occur. Our rationale for this belief stems from the following considerations. The newly synthesized RNA strand is likely to fold shortly after transcription. The RNA chaperone activity of the transcription machinery may only assist in folding a limited segment of the newly formed RNA. A stepwise mechanism may be utilized for pseudoknot formation. As the pseudoknot-forming sequence is transcribed, the initial event is the formation of a stem-loop structure with the stem corresponding to stem1 of the pseudoknot. Subsequently, as the next RNA segment is transcribed and folds, another stem-loop structure with the stem corresponding to the additional stem3 is formed. Finally, a following RNA segment containing nucleotides that are complementary to the loop region of the initially formed stem-loop becomes available; formation of stem2 leads to formation of the pseudoknot (Fig 7). This stepwise process inherently precludes the formation of the alternative overall stem-loop structure.

This mechanism implies that stem1 of the pseudoknot can form at the first place, if the 5’-sequence of stem1 interacts with the upstream sequence to form a stable structure, the interaction would prevent formation of stem1. Conversely, if the immediate upstream sequence of the pseudoknot folds into a stable structure, it would support the formation of stem1 of the pseudoknot. We evaluated these possibilities in the upstream sequences of the 14 selected mRNAs. In most cases, the upstream sequence of 20–30 nucleotides can form a relatively stable stem-loop structure. For example, in the MAB21L4 mRNA sequence shown in Fig 7, the identified three-stemmed pseudoknot spans nucleotides 24 to 84. The upstream sequence from this predicted pseudoknot can potentially form a stem-loop structure encompassing nucleotides 2 to 22. Similar pattern is also observed in the mRNAs of CHD5, CTDSPL, TMEM181, PPP2CB, KLHL2, SMAP2, SCN1B, BPHL, DNAI1, and SLIT2. In the mRNAs of SMIM10L2B, MRET1, and MYCL, the pseudoknot begins at the very 5’ end of the mRNA sequence.

This mechanism also sheds light on why naturally occurring pseudoknots rarely exhibit stem regions containing more than 15 base pairs, as revealed by the pseudoknots in the PseudoBase++ database [3, 4]. This phenomenon is exemplified in our previous study on −1 PRF and stop-codon readthrough stimulating pseudoknots in RNA viruses [70]. Furthermore, this stepwise mechanism has implications for *in vitro* studies involving these pseudoknots, such as structural determination. During sample preparation, which involves denaturation and renaturation of RNA molecules, the presence of alternative structures becomes more likely. This could result in structural heterogeneity within the sample, potentially complicating the research process.

It’s important to highlight that while this manuscript primarily focuses on a set of three-stemmed pseudoknots located in the start codon region of human mRNAs, these pseudoknots are just a subset of a larger pool of potential three-stemmed pseudoknots that have been identified across various regions of the full-length mRNA molecules. Ongoing investigations are currently exploring these other detected pseudoknots. Nevertheless, the results presented in this manuscript suggest that three-stemmed pseudoknots might possess a broader spectrum of functional roles beyond their previously established functions in −1 PRF or stop-codon readthrough regulation in viral mRNAs.

Interestingly, this study serendipitously revealed the presence of a tandem arrangement of three-stemmed pseudoknots in some of the mRNA sequences (Fig 3). This discovery suggests that three-stemmed pseudoknots (regardless of whether they have the potential for the extra stem to stack on the two essential stems) and their tandem arrangement may be more common than currently recognized.

Previously, we reported the presence of potential tandem pseudoknots at the −1 PRF junctions of various viruses, including HIV-1, transmissible gastroenteritis virus (TGEV), Barmah Forest virus (BFV), and Fort Morgan virus (FMV) [70, 71, 85]. In these previously reported instances, the slippery sequence is enclosed within the upstream pseudoknot. Additionally, in Equine arteritis virus (EAV), we identified a potential compact pseudoknot nestled within the loop2 sequence of an otherwise conventional frameshift-stimulating pseudoknot. The four stem regions of these two pseudoknots exhibit the potential to stack coaxially [85]. All of these previously reported tandem pseudoknots are found in viral RNAs. The potential presence of tandem pseudoknots in human SMIM10L2B and SMAP2 mRNAs could signify a significant discovery within the realm of cellular mRNAs. Notably, these two instances distinguish themselves from others because both pseudoknots in the tandem configuration possess the capacity to accommodate an additional stem. Thus, these two cases may possibly represent the initial documented occurrences of tandemly arranged three-stemmed pseudoknots.

Most of the mRNAs wherein potential three-stemmed pseudoknots have been identified (Figs 2 and 3) encode proteins that play important functional roles. Many of these proteins are directly implicated in various diseases. For example, MYCL, a transcription factor, is one of the three members within the Myc proto-oncogene family. Myc proteins are master regulators that modulate approximately 15% of the entire transcriptome. Their expression is tightly regulated in normal cells. But in over 70% of human cancers, overexpression of Myc proteins is observed and related to poor prognosis. Consequently, the Myc oncoproteins have been considered as drug targets. Despite this, there is currently no specific drug available to directly target Myc oncoproteins [62]. BPHL has been linked to the development of lung cancer. In lung adenocarcinoma cell lines, knockdown of *BPHL* gene expression through RNA interference resulted in a decrease in tumor growth, colony formation, and metastasis, while promoting an increase in apoptosis [63]. CHD5 and CTDSPL possess tumor suppressor activities in many cancers, including neuroblastomas, gliomas, and Non-Small Cell Lung Cancer (NSCLC) [65–67, 86]. SLIT2 plays crucial protective roles in various processes, including cell migration, immune response, vascular permeability, and angiogenesis during the development of the central nervous system (CNS). Consequently, SLIT2 could potentially be targeted for therapeutic interventions in central nervous system disorders [64].

Should the putative pseudoknot structures identified in this study prove to be real and play a crucial role on the translational regulation of the mRNAs, there is a possibility that these structures could be strategically targeted for therapeutic benefits. It is well established that pseudoknots, including three-stemmed pseudoknots such as those in the fluoride riboswitch and the preQ1–II riboswitch (Fig 1), are versatile RNA structures capable of binding various proteins, small organic molecules, and even ions as minuscule as the fluoride ion with high affinity and specificity [39, 40, 69, 79, 87–92]. The identification and validation of pseudoknots as crucial regulatory RNA structures in human mRNAs would not only lead to a better understanding of the regulatory mechanisms governing translational processes but also provide valuable targets for innovative drug development strategies.

The identification of potential three-stemmed pseudoknots within human mRNAs carries important implications for the development of anti-viral drugs targeting the SARS-CoV-2 frameshift-stimulating three-stemmed pseudoknot. As the possible presence of these three-stemmed pseudoknots in human RNAs is now recognized, it becomes crucial to assess whether medications designed for the SARS-CoV-2 pseudoknot could inadvertently affect similar RNA structures within human RNAs. This preemptive action aims to mitigate off-target effects in the development of antiviral treatments—an aspect that was previously overlooked.

## Materials and methods

### Data

The Data for this study consist of 21,780 human mRNA sequences obtained from the NCBI RefSeq database (www.ncbi.nlm.nih.gov/RefSeq/). This manuscript discusses 14 cases of potential three-stemmed pseudoknots identified in close proximity to the AUG start codon in the mRNA sequence. The accession ID and encoded protein of these 14 mRNAs are: NM_015557, chromodomain helicase DNA binding protein 5 (CHD5); NM_001008392, CTD small phosphatase like (CTDSPL), transcript variant 1; NM_007246, kelch like family member 2 (KLHL2), transcript variant 1; NM_022733, small ArfGAP2 (SMAP2), transcript variant 1; NM_001009552, protein phosphatase 2 catalytic subunit beta (PPP2CB); NM_001282921, mab-21 like 4 (MAB21L4), transcript variant 3; NM_001348255, small integral membrane protein 10 like 2B (SMIM10L2B); NM_001376852, transmembrane protein 181 (TMEM181), transcript variant 4; XM_027630882 Malassezia restricta dynactin 4 (MRET_4255), partial mRNA; NM_001037, sodium voltage-gated channel beta subunit 1 (SCN1B); NM_004332, biphenyl hydrolase like (BPHL), transcript variant 1; NM_005376, MYCL proto-oncogene, bHLH transcription factor (MYCL), transcript variant 3; NM_012144, dynein axonemal intermediate chain 1 (DNAI1), transcript variant 1; NM_004787, slit guidance ligand 2 (SLIT2), transcript variant 1.

### Detection of potential three-stemmed pseudoknots

Previously, we had developed a program called PKscan for the identification of potential RNA pseudoknots. Details of the program and the application of the program in a few large scale studies on full-length viral or cellular mRNAs have been described elsewhere [61, 71].

In brief, the formation of a pseudoknot in an RNA sequence relies on the presence of two distinct sets of complementary segments, giving rise to two separate stems, referred to as stem1 (S1) and stem2 (S2). These stems must be interspersed with either two or three unpaired loop regions, which are designated as loop 1 (L1), loop 2 (L2), and optionally, loop 3 (L3). The permissible lengths for S1, S2, L1, L2, and L3 can be customized by the user. The program systematically examines a broad spectrum of stem and loop lengths to determine if the prerequisites for pseudoknot formation are met. PKscan employs a dynamic sliding window that scan through the RNA sequence, allowing for the analysis of RNA sequences of any length without restriction. In each sliding window, iterative cycles of pairwise base matching are executed to identify complementary basepairing between two stretches of nucleotides. All conceivable combinations of stem and loop lengths within predefined ranges are investigated for potential pseudoknot formation.

PKscan possesses the ability to effectively detect RNA pseudoknots within RNA sequences of limitless length. This distinctive feature makes the PKscan program ideally suited for the specific requirements of this study.

In order to assess the comparative stability of the identified potential pseudoknots within a long RNA sequence, the program calculates the free energy of the helical stem regions of the detected pseudoknots based on the widely used Turner’s nearest neighbor parameters [93]. The computed free energy values also play a pivotal role in filtering out less stable pseudoknots. In the context of this current investigation, pseudoknots with a calculated free energy value higher than -18 kJ/mol are omitted from inclusion in the identified pseudoknot database. It’s important to emphasize that this free energy calculation solely takes into account the two pseudoknot-forming stems, namely stem1 and stem2. The presence of an additional stem (stem3) is not factored into this computation. The reasonales for these procedures is to ensure that the detection of potential existence of an extra stem will only be performed on those pseudoknots with a reasonably stable base structure. Those potential three-stemmed pseudoknots with weak basic stems (stem1 and stem2) but strong stem3 should not be detected (if the energy calculation includes all three stems, these pseudoknots would be detected due to contribution from the strong stem3, but the likelihood for pseudoknot formation is actually relatively low because of the weak basic stems). It’s worth noting that obtaining an accurate free energy calculation for RNA pseudoknots is currently unfeasible due to the absence of parameters capable of quantifying the influence of pseudoknot-specific structural characteristics. Moreover, case-specific interactions, such as those involving the minor groove and loop nucleotides, as well as base-triples, introduce further complexity and hinder the accurate prediction of the free energy associated with a projected pseudoknot based solely on the predicted secondary structures. That being said, the overall stability of an RNA pseudoknot is largely determined by interactions involving base pairing and base stacking in the stem regions. Consequently, the free energy required to form these stems can serve as a reasonable parameter for assessing the relative stability of the pseudoknots that have been detected. Utilizing these calculated free energy values, potential pseudoknots within a given mRNA sequence are ranked accordingly.

The initial iteration of the PKscan program did not assess the potential for an extra stem formation. In order to fulfill the particular objectives of this research, which involve identifying the potential existence of three-stem pseudoknots, we improved the program. This enhancement involved integrating the capability to detect the likelihood of forming a stem-loop structure immediately within the 5’-end sequence of loop2 (L2) within the detected pseudoknots. In this context, "immediate 5’-end" indicates that there is no intervening sequence between stem1 and stem3, allowing for the possibility of these two stems to align coaxially.

In this study, we restricted our search for pseudoknots belonging to the previously proposed CPK1 (short for common pseudoknot motif 1) family. Pseudoknots within the CPK1 category exhibit consistent structural characteristics, including the presence of a minimal 1–2 nucleotide in L1 that traverses the major groove of S2, comprising 6–7 base pairs, and the occurrence of coaxial stacking between the two stems without any intervening loop. Past research has demonstrated the prevalence of CPK1 pseudoknots in both naturally occurring systems and SELEX pseudoknots, underscoring the widespread occurrence and popularity of these particular pseudoknot structures [70, 76, 77]. To restrict the search for relatively compact CPK-1 pseudoknots, the ranges for stem1 (S1), stem2 (S2), loop1 (L1), loop2 (L2), and loop3 (L3) were set as follows: S1 ranged from 5 to 20 base pairs, S2 ranged from 6 to 7 base pairs, L1 ranged from 1 to 2 nucleotides, L2 ranged from 5 to 50 nucleotides, and L3 was set to 0 nucleotide.

Using the specified parameters, we conducted a comprehensive search for potential pseudoknots within each mRNA sequence in the dataset. Only pseudoknot candidates with a computed free energy lower than -18 kJ/mol were retained and cataloged in the pseudoknot database. These identified pseudoknots were subsequently ranked based on their calculated free energy values. Each detected pseudoknot was then subjected to further scrutiny by the program, with a focus on the possibility of containing a stem-loop structure in the 5’-end sequence of loop2. We defined the potential additional stem within a range of 3 to 15 base pairs, with no allowance for gap sequences between this additional stem and stem1.

In the search results output, we listed all detected pseudoknots in ascending order of their calculated free energy values, irrespective of whether a potential additional stem was found. For pseudoknots exhibiting the potential for an additional stem, we included pertinent information to signify the potential identification of a three-stemmed pseudoknot. Support information (S1 File), encompassing the output files for the nine potential cases discussed in this paper, are also provided for reference.

### Structural modelling of the three-stemmed pseudoknots

To investigate the structural feasibility of three-stemmed pseudoknots, we conducted modeling studies on specific pseudoknots. We built modeled structures for pseudoknots identified in the mRNAs of CHD5, CTDSPL, and SMIM10L2B. Notably, the mRNA sequence of SMIM10L2B exhibits the potential to harbor tandem pseudoknots, and our modeled structure incorporates both of these pseudoknots.

The modeling procedures employed in this study closely followed those in our previous publications [41, 94]. To determine the structures of RNA molecules, we utilized a combination of simulated annealing and restrained molecular dynamics, as incorporated within the NMR protocols of CNS (Crystallography and NMR System) version 1.3 [95].

For actual structure determination by NMR, various types of restraints derived from NMR data are employed, including NOE-based distance restraints, hydrogen bond restraints and dihedral angle restraints. In our investigations related to model building, we introduced artificial distance and torsion angle constraints for the helical stem regions of the molecules (including junctions between coaxially stacked stems). We did not apply any restraints to the single-stranded connecting loop regions.

In this study, various types of constraints were employed. In the helical stem regions, we utilized inter-residue proton-proton distance restraints reminiscent of NOE measurements observed in A-form RNA helices through NMR analysis. Additionally, we introduced distance restraints to regulate the distances between phosphorus atoms spanning the two strands. Distance restraints were used to represent the hydrogen bonds found in Watson-Crick G-C and A-U base pairs, as well as the G-U wobble base pair. Each hydrogen bond was subjected to two distance restraints, derived from the standard base pair geometry characteristic of A-form RNAs. Besides distance restraints, torsion angle restraints were also implemented to enforce specific conformations in the nucleotides. Nucleotide sugar rings in the stem regions were constrained to adopt a C3′-endo conformation. Nucleotide sugar rings in the loop regions were constrained to assume a C2′-endo conformation. Backbone torsion angles for residues in the stem regions were restricted to follow the values typical of the A-form helix; No restrains were used for the backbone torsion angles for nucleotides in the connecting loop regions. The modeled structures show no significant violations of the standard bond lengths and angles of A-form helical structures, indicating the stereochemical feasibility of the molecules.

We employed the Chimera program, developed by the computer graphics laboratory at UCSF [96], to manually create the initial input structures for the structure calculation process. The initial structures were subjected to simulated annealing in the torsion angle space, followed by variable target function minimization. The selection of the most suitable model structures was based on their low target function values. All 3D RNA structure figures were generated exclusively using Chimera.

### BLASTN analysis

We conducted a conservation analysis using the Basic Local Alignment Search Tool for Nucleotide sequences (BLASTN). The BLASTN algorithm, accessible via the NCBI (National Center for Biotechnology Information) website at https://blast.ncbi.nlm.nih.gov/Blast.cgi, was employed, using the sequence forming the detected potential three-stemmed pseudoknot as the query. The results of the sequence analysis have been included as support information (S2 File).

## Supporting information

S1 File Program search output. (PDF)

S2 File Sequence analysis. (PDF)

S3 File Using PKscan to identify pseudoknots within the SRV-1 genome. (PDF)

## References

[1] Pleij CW, Rietveld K, Bosch L. A new principle of RNA folding based on pseudoknotting. Nucleic Acids Res. 1985;13(5):1717–31. doi: 10.1093/nar/13.5.1717 4000943 PMC341107

[2] Pleij CW. Pseudoknots: a new motif in the RNA game. Trends in Biochemical Science. 1990;15:143–7. doi: 10.1016/0968-0004(90)90214-v 1692647

[3] van Batenburg FHD, Gultyaev AP, Pleij CWA. PseudoBase: structural information on RNA pseudoknots. Nucl Acids Res. 2001;29(1):194–5. doi: 10.1093/nar/29.1.194 11125088 PMC29770

[4] Taufer M, Licon A, Araiza R, Mireles D, van Batenburg FH, Gultyaev AP, et al. PseudoBase++: an extension of PseudoBase for easy searching, formatting and visualization of pseudoknots. Nucleic Acids Res. 2009;37(Database issue):D127–35. doi: 10.1093/nar/gkn806 18988624 PMC2686561

[5] Peselis A, Serganov A. Structure and function of pseudoknots involved in gene expression control. Wiley Interdiscip Rev RNA. 2014;5(6):803–22. doi: 10.1002/wrna.1247 25044223 PMC4664075

[6] Hill CH, Brierley I. Structural and Functional Insights into Viral Programmed Ribosomal Frameshifting. Annu Rev Virol. 2023;10(1):217–42. doi: 10.1146/annurev-virology-111821-120646 37339768 PMC7618472

[7] Giedroc DP, Cornish PV. Frameshifting RNA pseudoknots: structure and mechanism. Virus Res. 2009;139(2):193–208. doi: 10.1016/j.virusres.2008.06.008 18621088 PMC2670756

[8] Gesteland RF, Atkins JF. Recoding: dynamic reprogramming of translation. Annu Rev Biochem. 1996;65:741–68. doi: 10.1146/annurev.bi.65.070196.003521 8811194

[9] Baranov pv, Gesteland RF, Atkins JF. Recoding: translational bifurcations in gene expression. Gene. 2002;286(2):187–201. doi: 10.1016/s0378-1119(02)00423-7 11943474

[10] Gesteland RF, Weiss RB, Atkins JF. Recoding: reprogrammed genetic decoding. Science. 1992;257:1640–1. doi: 10.1126/science.1529352 1529352

[11] Wills N, Gesteland R, Atkins J. Evidence that a Downstream Pseudoknot is Required for Translational Read- Through of the Moloney Murine Leukemia Virus Gag Stop Codon. PNAS. 1991;88(16):6991–5. doi: 10.1073/pnas.88.16.6991 1871115 PMC52219

[12] Feng YX, Yuan H, Rein A, Levin JG. Bipartite signal for read-through suppression in murine leukemia virus mRNA: an eight-nucleotide purine-rich sequence immediately downstream of the gag termination codon followed by an RNA pseudoknot. J Virol. 1992;66(8):5127–32. doi: 10.1128/JVI.66.8.5127-5132.1992 1629968 PMC241386

[13] Brierley I. Ribosomal frameshifting viral RNAs. J Gen Virol. 1995;76 ((Pt 8)):1885–92. doi: 10.1099/0022-1317-76-8-1885 7636469

[14] Farabaugh PJ. Programmed translational frameshifting. Microbiol Rev. 1996;60(1):103–34. doi: 10.1128/mr.60.1.103-134.1996 8852897 PMC239420

[15] Giedroc DP, Theimer CA, Nixon PL. Structure, stability and function of RNA pseudoknots involved in stimulating ribosomal frameshifting. J Mol Biol. 2000;298(2):167–85. doi: 10.1006/jmbi.2000.3668 10764589 PMC7126452

[16] Brierley I, Pennell S, Gilbert RJ. Viral RNA pseudoknots: versatile motifs in gene expression and replication. Nature reviews Microbiology. 2007;5(8):598–610. doi: 10.1038/nrmicro1704 17632571 PMC7096944

[17] Bekaert M, Firth AE, Zhang Y, Gladyshev VN, Atkins JF, Baranov PV. Recode-2: new design, new search tools, and many more genes. Nucleic Acids Research. 2010;38(suppl 1):D69–D74. doi: 10.1093/nar/gkp788 19783826 PMC2808893

[18] Dam Et, Pleij K, Draper D. Structural and functional aspects of RNA pseudoknots. Biochemistry. 1992;31:11665–76. doi: 10.1021/bi00162a001 1280160

[19] Baranov PV, Henderson CM, Anderson CB, Gesteland RF, Atkins JF, Howard MT. Programmed ribosomal frameshifting in decoding the SARS-CoV genome. Virology. 2005;332(2):498–510. doi: 10.1016/j.virol.2004.11.038 15680415 PMC7111862

[20] Cho CP, Lin SC, Chou MY, Hsu HT, Chang KY. Regulation of programmed ribosomal frameshifting by co-translational refolding RNA hairpins. PLoS One. 2013;8(4):e62283. doi: 10.1371/journal.pone.0062283 23638024 PMC3639245

[21] Dos Ramos F, Carrasco M, Doyle T, Brierley I. Programmed -1 ribosomal frameshifting in the SARS coronavirus. Biochem Soc Trans. 2004;32(Pt 6):1081–3. doi: 10.1042/BST0321081 15506971

[22] Plant EP, Perez-Alvarado GC, Jacobs JL, Mukhopadhyay B, Hennig M, Dinman JD. A three-stemmed mRNA pseudoknot in the SARS coronavirus frameshift signal. PLoS Biol. 2005;3(6):e172. doi: 10.1371/journal.pbio.0030172 15884978 PMC1110908

[23] Su MC, Chang CT, Chu CH, Tsai CH, Chang KY. An atypical RNA pseudoknot stimulator and an upstream attenuation signal for -1 ribosomal frameshifting of SARS coronavirus. Nucleic Acids Res. 2005;33(13):4265–75. doi: 10.1093/nar/gki731 16055920 PMC1182165

[24] Miller WA, Waterhouse PM, Gerlach WL. Sequence and organization of barley yellow dwarf virus genomic RNA. Nucleic Acids Res. 1988;16(13):6097–111. doi: 10.1093/nar/16.13.6097 3399386 PMC336850

[25] Kujawa AB, Drugeon G, Hulanicka D, Haenni AL. Structural requirements for efficient translational frameshifting in the synthesis of the putative viral RNA-dependent RNA polymerase of potato leafroll virus. Nucleic Acids Res. 1993;21(9):2165–71. doi: 10.1093/nar/21.9.2165 8502558 PMC309480

[26] Cornish PV, Hennig M, Giedroc DP. A loop 2 cytidine-stem 1 minor groove interaction as a positive determinant for pseudoknot-stimulated -1 ribosomal frameshifting. Proc Natl Acad Sci U S A. 2005;102(36):12694–9. doi: 10.1073/pnas.0506166102 16123125 PMC1200304

[27] Michiels PJ, Versleijen AA, Verlaan PW, Pleij CW, Hilbers CW, Heus HA. Solution structure of the pseudoknot of SRV-1 RNA, involved in ribosomal frameshifting. J Mol Biol. 2001;310(5):1109–23. doi: 10.1006/jmbi.2001.4823 11501999 PMC7172549

[28] Dam Et, Brierley I, Inglis S, Pleij CW. Identification and analysis of the pseudoknot-containing gag-pro ribosomal frameshift signal of simian retrovirus-1. Nucleic Acids Res. 1994;22(12.):2304–10. doi: 10.1093/nar/22.12.2304 8036158 PMC523688

[29] Dam EBt, Verlaan PW, Pleij CW. Analysis of the role of the pseudoknot component in the SRV-1 gag-pro ribosomal frameshift signal: loop lengths and stability of the stem regions. RNA. 1995;1(2):146–54. 7585244 PMC1369068

[30] Wills N, Gesteland R, Atkins J. Pseudoknot-dependent read-through of retroviral gag termination codons: importance of sequences in the spacer and loop 2. EMBO J. 1994;13(17):4137–44. doi: 10.1002/j.1460-2075.1994.tb06731.x 8076609 PMC395336

[31] Houck-Loomis B, Durney MA, Salguero C, Shankar N, Nagle JM, Goff SP, et al. An equilibrium-dependent retroviral mRNA switch regulates translational recoding. Nature. 2011;480(7378):561–4. doi: 10.1038/nature10657 22121021 PMC3582340

[32] Baril M, Dulude D, Steinberg SV, Brakier-Gingras L. The Frameshift Stimulatory Signal of Human Immunodeficiency Virus Type 1 Group O is a Pseudoknot. J Mol Biol. 2003;331(3):571–83. doi: 10.1016/s0022-2836(03)00784-8 12899829 PMC7127721

[33] Han K, Byun Y. PSEUDOVIEWER2: Visualization of RNA pseudoknots of any type. Nucleic Acids Res. 2003;31(13):3432–40. doi: 10.1093/nar/gkg539 12824341 PMC168946

[34] Kelly JA, Olson AN, Neupane K, Munshi S, San Emeterio J, Pollack L, et al. Structural and functional conservation of the programmed -1 ribosomal frameshift signal of SARS coronavirus 2 (SARS-CoV-2). J Biol Chem. 2020;295(31):10741–8. doi: 10.1074/jbc.AC120.013449 32571880 PMC7397099

[35] Roman C, Lewicka A, Koirala D, Li NS, Piccirilli JA. The SARS-CoV-2 Programmed -1 Ribosomal Frameshifting Element Crystal Structure Solved to 2.09 Å Using Chaperone-Assisted RNA Crystallography. ACS Chem Biol. 2021;16(8):1469–81. 34328734 10.1021/acschembio.1c00324 PMC8353986

[36] Jones CP, Ferré-D’Amaré AR. Crystal structure of the severe acute respiratory syndrome coronavirus 2 (SARS-CoV-2) frameshifting pseudoknot. Rna. 2022;28(2):239–49. doi: 10.1261/rna.078825.121 34845084 PMC8906546

[37] Bhatt PR, Scaiola A, Loughran G, Leibundgut M, Kratzel A, Meurs R, et al. Structural basis of ribosomal frameshifting during translation of the SARS-CoV-2 RNA genome. Science. 2021;372(6548):1306–13. doi: 10.1126/science.abf3546 34029205 PMC8168617

[38] Zhang K, Zheludev IN, Hagey RJ, Haslecker R, Hou YJ, Kretsch R, et al. Cryo-EM and antisense targeting of the 28-kDa frameshift stimulation element from the SARS-CoV-2 RNA genome. Nat Struct Mol Biol. 2021;28(9):747–54. doi: 10.1038/s41594-021-00653-y 34426697 PMC8848339

[39] Ren A, Rajashankar KR, Patel DJ. Fluoride ion encapsulation by Mg2+ ions and phosphates in a fluoride riboswitch. Nature. 2012;486(7401):85–9. doi: 10.1038/nature11152 22678284 PMC3744881

[40] Liberman JA, Salim M, Krucinska J, Wedekind JE. Structure of a class II preQ1 riboswitch reveals ligand recognition by a new fold. Nature chemical biology. 2013;9(6):353–5. doi: 10.1038/nchembio.1231 23584677 PMC3661761

[41] Huang X, Du Z. Elaborated pseudoknots that stimulate -1 programmed ribosomal frameshifting or stop codon readthrough in RNA viruses. Journal of biomolecular structure & dynamics. 2023:1–13. doi: 10.1080/07391102.2023.2292296 38095458 PMC11176267

[42] Liphardt J, Napthine s, Kontos h, Brierley i. Evidence for an RNA pseudoknot loop-helix interaction essential for efficient -1 ribosomal frameshifting. J Mol Biol. 1999;288(3):321–35. doi: 10.1006/jmbi.1999.2689 10329145 PMC7141562

[43] Napthine S, Liphardt J, Bloys A, Routledge S, Brierley I. The role of RNA pseudoknot stem 1 length in the promotion of efficient -1 ribosomal frameshifting. J Mol Biol. 1999;288(3):305–20. doi: 10.1006/jmbi.1999.2688 10329144 PMC7126229

[44] Alam SL, Wills NM, Ingram JA, Atkins JF, Gesteland RF. Structural studies of the RNA pseudoknot required for readthrough of the gag-termination codon of murine leukemia virus. J Mol Biol. 1999;288(5):837–52. doi: 10.1006/jmbi.1999.2713 10329183

[45] Ben-Asouli Y, Banai Y, Pel-Or Y, Shir A, Kaempfer R. Human interferon-gamma mRNA autoregulates its translation through a pseudoknot that activates the interferon-inducible protein kinase PKR. Cell. 2002;108(2):221–32. doi: 10.1016/s0092-8674(02)00616-5 11832212

[46] Barrette I, Poisson G, Gendron P, Major F. Pseudoknots in prion protein mRNAs confirmed by comparative sequence analysis and pattern searching. Nucleic Acids Res. 2001;29(3):753–8. doi: 10.1093/nar/29.3.753 11160898 PMC30388

[47] Belew AT, Meskauskas A, Musalgaonkar S, Advani VM, Sulima SO, Kasprzak WK, et al. Ribosomal frameshifting in the CCR5 mRNA is regulated by miRNAs and the NMD pathway. Nature. 2014;512(7514):265–9. doi: 10.1038/nature13429 25043019 PMC4369343

[48] Koonin EV, Dolja VV. A virocentric perspective on the evolution of life. Curr Opin Virol. 2013;3(5):546–57. doi: 10.1016/j.coviro.2013.06.008 23850169 PMC4326007

[49] Sato K, Kato Y. Prediction of RNA secondary structure including pseudoknots for long sequences. Brief Bioinform. 2022;23(1). doi: 10.1093/bib/bbab395 34601552 PMC8769711

[50] Andronescu MS, Pop C, Condon AE. Improved free energy parameters for RNA pseudoknotted secondary structure prediction. Rna. 2010;16(1):26–42. doi: 10.1261/rna.1689910 19933322 PMC2802035

[51] Sperschneider J, Datta A. DotKnot: pseudoknot prediction using the probability dot plot under a refined energy model. Nucleic Acids Res. 2010;38(7):e103. doi: 10.1093/nar/gkq021 20123730 PMC2853144

[52] Bellaousov S, Mathews DH. ProbKnot: fast prediction of RNA secondary structure including pseudoknots. Rna. 2010;16(10):1870–80. doi: 10.1261/rna.2125310 20699301 PMC2941096

[53] Xayaphoummine A, Bucher T, Isambert H. Kinefold web server for RNA/DNA folding path and structure prediction including pseudoknots and knots. Nucleic Acids Res. 2005;33(Web Server issue):W605–10. doi: 10.1093/nar/gki447 15980546 PMC1160208

[54] Dawson WK, Fujiwara K, Kawai G. Prediction of RNA pseudoknots using heuristic modeling with mapping and sequential folding. PLoS One. 2007;2(9):e905. doi: 10.1371/journal.pone.0000905 17878940 PMC1975678

[55] Bindewald E, Kluth T, Shapiro BA. CyloFold: secondary structure prediction including pseudoknots. Nucleic Acids Res. 2010;38(Web Server issue):W368–72. doi: 10.1093/nar/gkq432 20501603 PMC2896150

[56] Janssen S, Giegerich R. The RNA shapes studio. Bioinformatics (Oxford, England). 2015;31(3):423–5. doi: 10.1093/bioinformatics/btu649 25273103 PMC4308662

[57] Cheng Y, Zhang S, Xu X, Chen SJ. Vfold2D-MC: A Physics-Based Hybrid Model for Predicting RNA Secondary Structure Folding. J Phys Chem B. 2021;125(36):10108–18. doi: 10.1021/acs.jpcb.1c04731 34473508 PMC8903033

[58] Wang W, Feng C, Han R, Wang Z, Ye L, Du Z, et al. trRosettaRNA: automated prediction of RNA 3D structure with transformer network. Nature communications. 2023;14(1):7266. doi: 10.1038/s41467-023-42528-4 37945552 PMC10636060

[59] Parisien M, Major F. The MC-Fold and MC-Sym pipeline infers RNA structure from sequence data. Nature. 2008;452(7183):51–5. doi: 10.1038/nature06684 18322526

[60] Huang X, Cheng Q, Du Z. Possible utilization of -1 Ribosomal frame shifting in the expression of a human SEMA6C isoform. Bioinformation. 2013;9(14):736–8. doi: 10.6026/97320630009736 23976831 PMC3746098

[61] Huang X, Du Z, Cheng J, Cheng Q. PKscan: a program to identify H-type RNA pseudoknots in any RNA sequence with unlimited length. Bioinformation. 2013;9(9):440–2. doi: 10.6026/97320630009440 23847396 PMC3705612

[62] Wang C, Zhang J, Yin J, Gan Y, Xu S, Gu Y, et al. Alternative approaches to target Myc for cancer treatment. Signal Transduct Target Ther. 2021;6(1):117. doi: 10.1038/s41392-021-00500-y 33692331 PMC7946937

[63] Ren P, Zhai J, Wang X, Yin Y, Lin Z, Cai K, et al. Inhibition of BPHL inhibits proliferation in lung carcinoma cell lines. Transl Lung Cancer Res. 2023;12(5):1051–61. doi: 10.21037/tlcr-23-225 37323178 PMC10261862

[64] Sherchan P, Travis ZD, Tang J, Zhang JH. The potential of Slit2 as a therapeutic target for central nervous system disorders. Expert Opin Ther Targets. 2020;24(8):805–18. doi: 10.1080/14728222.2020.1766445 32378435 PMC7529836

[65] Otani R, Sadato D, Yamada R, Yajima H, Kawamura S, Shimizu S, et al. CHD5 gene variant predicts leptomeningeal metastasis after surgical resection of brain metastases of breast cancer. J Neurooncol. 2023;163(3):657–62. doi: 10.1007/s11060-023-04381-9 37440096

[66] Xu L, Shao F, Luo T, Li Q, Tan D, Tan Y. Pan-Cancer Analysis Identifies CHD5 as a Potential Biomarker for Glioma. Int J Mol Sci. 2022;23(15). doi: 10.3390/ijms23158489 35955624 PMC9369136

[67] Moudi B, Asemi-Rad A, Sheibak N, Heidari Z, Mahmoudzadeh-Sagheb H. The possible impact of DNA-binding chromodomain-helicase 5 polymorphisms on male infertility: A case-control study. J Obstet Gynaecol Res. 2023;49(4):1214–21. doi: 10.1111/jog.15560 36695418

[68] Du Z, Holland JA, Hansen MR, Giedroc DP, Hoffman DW. Base-pairings within the RNA pseudoknot associated with the simian retrovirus-1 gag-pro frameshift site. J Mol Biol. 1997;270(3):464–70. doi: 10.1006/jmbi.1997.1127 9237911

[69] Du Z, Giedroc DP, Hoffman DW. Structure of the Autoregulatory Pseudoknot within the Gene 32 Messenger RNA of Bacteriophages T2 and T6: A Model for a Possible Family of Structurally Related RNA Pseudoknots. Biochemistry. 1996;35:4187–98. doi: 10.1021/bi9527350 8672455

[70] Huang X, Cheng Q, Du Z. A genome-wide analysis of RNA pseudoknots that stimulate efficient -1 ribosomal frameshifting or readthrough in animal viruses. Biomed Res Int. 2013;2013:984028. doi: 10.1155/2013/984028 24298557 PMC3835772

[71] Huang X, Yang Y, Wang G, Cheng Q, Du Z. Highly conserved RNA pseudoknots at the gag-pol junction of HIV-1 suggest a novel mechanism of -1 ribosomal frameshifting. RNA. 2014;20(5):587–93. doi: 10.1261/rna.042457.113 24671765 PMC3988561

[72] Zhu J HX, Du Z. Widespread Occurrence of CPK-1 Pseudoknots and Related RNA Structures. Research Journal of Life Sciences, Bioinformatics, Pharmaceutical and Chemical Sciences. 2017;3(1):1–16.

[73] Neupane K, Munshi S, Zhao M, Ritchie DB, Ileperuma SM, Woodside MT. Anti-Frameshifting Ligand Active against SARS Coronavirus-2 Is Resistant to Natural Mutations of the Frameshift-Stimulatory Pseudoknot. J Mol Biol. 2020;432(21):5843–7. doi: 10.1016/j.jmb.2020.09.006 32920049 PMC7483078

[74] Sun Y, Abriola L, Surovtseva YV, Lindenbach BD, Guo JU. Restriction of SARS-CoV-2 Replication by Targeting Programmed -1 Ribosomal Frameshifting In Vitro. bioRxiv. 2020. doi: 10.1101/2020.10.21.349225 34185680 PMC8256030

[75] Zhang K, Zheludev IN, Hagey RJ, Wu MT, Haslecker R, Hou YJ, et al. Cryo-electron Microscopy and Exploratory Antisense Targeting of the 28-kDa Frameshift Stimulation Element from the SARS-CoV-2 RNA Genome. bioRxiv. 2020. doi: 10.1101/2020.07.18.209270 34426697 PMC8848339

[76] Du Z, Giedroc DP, Hoffman DW. Structure of the autoregulatory pseudoknot within the gene 32 messenger RNA of bacteriophages T2 and T6: a model for a possible family of structurally related RNA pseudoknots. Biochemistry. 1996;35(13):4187–98. doi: 10.1021/bi9527350 8672455

[77] Du Z, Hoffman D. An NMR and mutational study of the pseudoknot within the gene 32 mRNA of bacteriophage T2: insights into a family of structurally related RNA pseudoknots. Nucl Acids Res. 1997;25(6):1130–5. doi: 10.1093/nar/25.6.1130 9092620 PMC146565

[78] Brierley I, Digard P, Inglis SC. Characterization of an efficient coronavirus ribosomal frameshifting signal: requirement for an RNA pseudoknot. Cell. 1989;57.(4):537–47. doi: 10.1016/0092-8674(89)90124-4 2720781 PMC7133225

[79] McPheeters DS, Stormo GD, Gold L. Autogenous regulatory site on the bacteriophage T4 gene 32 messenger RNA. J Mol Biol. 1988;201(3):517–35. doi: 10.1016/0022-2836(88)90634-1 3262167

[80] Priore SF, Kauffmann AD, Baman JR, Turner DH. The Influenza A PB1-F2 and N40 Start Codons Are Contained within an RNA Pseudoknot. Biochemistry. 2015;54(22):3413–5. doi: 10.1021/bi501564d 25996464 PMC4597466

[81] Tanaka T, Otoguro T, Yamashita A, Kasai H, Fukuhara T, Matsuura Y, et al. Roles of the 5’ Untranslated Region of Nonprimate Hepacivirus in Translation Initiation and Viral Replication. J Virol. 2018;92(7). doi: 10.1128/JVI.01997-17 29343570 PMC5972865

[82] Jopling CL, Spriggs KA, Mitchell SA, Stoneley M, Willis AE. L-Myc protein synthesis is initiated by internal ribosome entry. Rna. 2004;10(2):287–98. doi: 10.1261/rna.5138804 14730027 PMC1370540

[83] Advani VM, Dinman JD. Reprogramming the genetic code: The emerging role of ribosomal frameshifting in regulating cellular gene expression. Bioessays. 2016;38(1):21–6. doi: 10.1002/bies.201500131 26661048 PMC4749135

[84] Zuker M. Mfold web server for nucleic acid folding and hybridization prediction. Nucleic Acids Research. 2003;31:3406–15. doi: 10.1093/nar/gkg595 12824337 PMC169194

[85] Wang G, Yang Y, Huang X, Du Z. Possible involvement of coaxially stacked double pseudoknots in the regulation of -1 programmed ribosomal frameshifting in RNA viruses. Journal of biomolecular structure & dynamics. 2014:1–11. doi: 10.1080/07391102.2014.956149 25204560

[86] Krasnov GS, Puzanov GA, Afanasyeva MA, Dashinimaev EB, Vishnyakova KS, Beniaminov AD, et al. Tumor suppressor properties of the small C-terminal domain phosphatases in non-small cell lung cancer. Biosci Rep. 2019;39(12). doi: 10.1042/BSR20193094 31774910 PMC6911153

[87] Qiu H, Kaluarachchi K, Du Z, Hoffman DW, Giedroc DP. Thermodynamics of folding of the RNA pseudoknot of the T4 gene 32 autoregulatory messenger RNA. Biochemistry. 1996;35(13):4176–86. doi: 10.1021/bi9527348 8672454

[88] Philippe C, Benard L, Portier C, Westhof E, Ehresmann B, Ehresmann C. Molecular dissection of the pseudoknot governing the translational regulation of Escherichia coli ribosomal protein S15. Nucleic Acids Res. 1995;23(1):18–28. doi: 10.1093/nar/23.1.18 7532857 PMC306625

[89] Brown M, Wilson C. RNA pseudoknot modeling using intersections of stochastic context free grammars with applications to database search. Pacific Symposium on Biocomputing Pacific Symposium on Biocomputing. 1996:109–25. 9390227

[90] Nix J, Sussman D, Wilson C. The 1.3 angstrom crystal structure of a biotin-binding pseudoknot and the basis for RNA molecular recognition. J Mol Biol. 2000;296(5):1235–44. 10698630 10.1006/jmbi.2000.3539

[91] Hofstadler SA, Sannes-Lowery KA, Crooke ST, Ecker DJ, Sasmor H, Manalili S, et al. Multiplexed screening of neutral mass-tagged RNA targets against ligand libraries with electrospray ionization FTICR MS: a paradigm for high-throughput affinity screening. Anal Chem. 1999;71(16):3436–40. doi: 10.1021/ac990262n 10464476

[92] Jaeger J, Restle T, Steitz TA. The structure of HIV-1 reverse transcriptase complexed with an RNA pseudoknot inhibitor. EMBO J. 1998;17(15):4535–42. doi: 10.1093/emboj/17.15.4535 9687519 PMC1170784

[93] Mathews DH, Disney MD, Childs JL, Schroeder SJ, Zuker M, Turner DH. Incorporating chemical modification constraints into a dynamic programming algorithm for prediction of RNA secondary structure. Proc Natl Acad Sci U S A. 2004;101(19):7287–92. doi: 10.1073/pnas.0401799101 15123812 PMC409911

[94] Zhou X, Du Z, Huang X. A potential long-range RNA-RNA interaction in the HIV-1 RNA. Journal of biomolecular structure & dynamics. 2023:1–9. doi: 10.1080/07391102.2023.2184639 36863767

[95] Brünger AT, Adams PD, Clore GM, DeLano WL, Gros P, GrosseKunstleve RW, et al. Crystallography + NMR System: A new software suite for macromolecular structure determination. Acta Crystallogr. 1998;D54(PT5):905–21. 10.1107/s0907444998003254 9757107

[96] Pettersen EF, Goddard TD, Huang CC, Couch GS, Greenblatt DM, Meng EC, et al. UCSF Chimera—a visualization system for exploratory research and analysis. J Comput Chem. 2004;25(13):1605–12. doi: 10.1002/jcc.20084 15264254

