# Supplementary material for: Possible involvement of three-stemmed pseudoknots in regulating translational initiation in human mRNAs
Source: PLoS One. 2024 Jul 22;19(7):e0307541. doi: 10.1371/journal.pone.0307541 (PMC11262651; doi:10.1371/journal.pone.0307541)
Supplement: S1 File — (PDF) [file pone.0307541.s001.pdf]

This document includes the search results for the nine mRNAs where three-stemmed pseudoknots were detected near the AUG start codon and subsequently discussed in the manuscript

The accession ID and encoded protein of these nine mRNAs are:

NM_015557, Homo sapiens chromodomain helicase DNA binding protein 5 (CHD5); NM_001008392, Homo sapiens CTD small phosphatase like (CTDSPL), transcript variant 1; NM_007246, Homo sapiens kelch like family member 2 (KLHL2), transcript variant 1; NM_022733, Homo sapiens small ArfGAP2 (SMAP2), transcript variant 1;
NM_001009552 Homo sapiens protein phosphatase 2 catalytic subunit beta (PPP2CB); NM_001282921 mab-21 like 4 (MAB21L4), transcript variant 3;
NM_001348255 small integral membrane protein 10 like 2B (SMIM10L2B);
NM_001376852 Homo sapiens transmembrane protein 181 (TMEM181), transcript variant 4; XM_027630882 Malassezia restricta dynactin 4 (MRET_4255), partial mRNA;
NM_001037 sodium voltage-gated channel beta subunit 1 (SCN1B);
NM_004332, biphenyl hydrolase like (BPHL), transcript variant 1;
NM_005376, MYCL proto-oncogene, bHLH transcription factor (MYCL), transcript variant 3;
NM_012144, dynein axonemal intermediate chain 1 (DNAI1), transcript variant 1;
NM_004787, slit guidance ligand 2 (SLIT2), transcript variant 1.

In the output file, you'll find a list of all detected pseudoknots, regardless of whether an additional potential stem3 is detected, provided their computed free energy values are below -18 kJ/mol. These pseudoknots are arranged in ascending order of energy values. For pseudoknots with an additional potential stem3, the file also contains information about this additional stem.

How to read the output file.
The NM_015557 CHD5 mRNA output file serves as an illustrative example. The content enclosed between the two sets of asterisk lines represents the actual output for the highest-ranked pseudoknot; lower-ranked pseudoknots are omitted.

```
***************************************************************
Sequence #: NM_015557
Start=299 End=6163

loaded 9850 nucleotides, cds_start 299 to cds_end 6163

Energy 1: -41.140000: Start=258 S1=10 S2=6 L1=2 L2=25 L3=0 End=316

                    CCCCGCGCCGGGCATGCGGGGCCCA       GC

                    TCCTCCCCTC    CACCCG

AGGCTGGCACCGCGCCCGGCGGGAGGGGGG    GTGGGCAC

Found #1 stem loop within loop2: S3=7 AL=7 L2_tail=4 loop2(286-309)
cds_start=299
(CCCCGCG)CCGGGCA(TGCGGGG)CCCA
```

****

Any non-obvious fields are explained as follows:
Energy 1: -41.140000, computed free energy, with the number 1 denoting its rank

Start=258	the detected pseudoknot starts at nucleotide 258
S1=10	stem1 has 10 basepairs
S2=6	stem2 has 6 basepairs
L1=2	loop1 has 2 nucleotides
L2=25	loop2 has 25 nucleotides
L3=0	loop3 has 0 nucleotide (absent)
End=316	the detected pseudoknot ends at nucleotide 316

The actual sequence of the pseudoknot (plus 20 nt at the 5'-end and 2 nt at the 3'-end) is printed out in the output file. To aid in explanation, color backgrounds are employed to highlight different elements within the pseudoknot:

Cyan: stem1
Green: stem2
Magenta: loop1
Yellow: loop2
Deep grey: 5'-sequence
Pale grey: 3'-sequence

The corresponding linear sequence is (note that the top strand of stem1 and stem2 in the output orients from 3' to 5', thus need to be reversed in the linear sequence):

```
5'-
AGGCTGGCACCGCGCCCGGCGGGAGGGGGGGCGCCCACCTCCCCTCCTCCCCGCGCCGGGCATGCGGGGC
CCAGTGGGCAC-3'
```

In this pseudoknot, a potential stem-loop is detected within loop2, indicated by the following information in the output file:

```
Found #1 stem loop within loop2: S3=7 AL=7 L2_tail=4 loop2(286-309)
cds_start=299
(CCCCGCG)CCGGGCA(TGCGGGG)CCCA
```

S3=7	the additional stem3 has 7 basepairs
AL=7	the apical loop of the additional stem-loop structure has 7 nucleotides
L2_tail=4	after forming the additional stem-loop, 4 nucleotides are left in loop2

The loop2 sequence is reiterated, with parentheses denoting the stem regions:

```
(CCCCGCG)CCGGGCA(TGCGGGG)CCCA
```

The output files for the 14 mRNAs are copied as follows.

1) Output file for NM_015557, CHD5 mRNA

```
Sequence #: NM_015557
Start=299 End=6163
loaded 9850 nucleotides, cds_start 299 to cds_end 6163

Energy 1: -41.140000: Start=258 S1=10 S2=6 L1=2 L2=25 L3=0 End=316

                    CCCCGCGCCGGGCATGCGGGGCCCA       GC

                    TCCTCCCCTC    CACCCG

AGGCTGGCACCGCGCCCGGCGGGAGGGGGG    GTGGGCAC

Found #1 stem loop within loop2: S3=7 AL=7 L2_tail=4 loop2(286-309)
cds_start=299
(CCCCGCG)CCGGGCA(TGCGGGG)CCCA


Energy 2: -34.695000: Start=7194 S1=7 S2=7 L1=2 L2=34 L3=0 End=7257

                    ACTTGTCCCATTCCCCTGTCCCTGGAAAGGCCTT       GC

                    GGTCCGG    GGGTCCC

GCCCACCTCCGGAGGTGGAACCGGGCT    CCCGGGGGC


Energy 3: -29.890000: Start=6080 S1=6 S2=6 L1=2 L2=5 L3=0 End=6110

                    TGGAC       AA

                    CCCGGG    GCCTTC

AGATGTACAGCAACAACTTTGGGCCC    CGGGAGGG


Energy 4: -26.190000: Start=286 S1=7 S2=6 L1=1 L2=32 L3=0 End=344

                    CCCAGTGGGCACCGAGGAGGAGCTGCCGCGGC       C

                    GGGGCGT    ACGGGC

GGGCGCCCACCTCCCCTCCTCCCCGCG    TGTTCGCC


Energy 5: -25.290000: Start=44 S1=6 S2=6 L1=2 L2=18 L3=0 End=87

                    GGCGCGGTGGGAGGAGCG       GA

                    GGCGTC    GGGCTC
```

```
CCGCCGCCACCCTCGGCCGGCTGCGG    CCCGGGCT

Energy 6: -24.750000: Start=6754 S1=6 S2=6 L1=1 L2=7 L3=0 End=6785

                    GGAGCCG       A

                    GGTTTG    GACGGG

CCGCTTGGAGCCTCCCAGCACCAGAC    CTGTCCAG

Energy 7: -24.730000: Start=7920 S1=6 S2=6 L1=2 L2=34 L3=0 End=7979

                    GGGAGCACCCTGGAGTGGAGATGATTGTCAGCGG       AC

                    GTGATG    CGGGTG

CAAGGCCAGCCCGACCCTGACACTGC    GCTCACAT

Energy 8: -24.445000: Start=9146 S1=5 S2=7 L1=2 L2=29 L3=0 End=9200

                    GCGTGGGTGGGGGCCGAGGCTGGCACGCC       GG

                    GGACA    GGGGACG

GCCTCCAGTGTGCGTCAGGGTCTGT    CCTCTGCCC

Energy 9: -24.130000: Start=16 S1=5 S2=6 L1=1 L2=34 L3=0 End=72

                    CCCTCGGCCGGCTGCGGGACTCGGGCTGCGGGGC       C

                    ACCGC    CGCCGT

     GTGCCGCCGTGCCTCTGGCG    GCGGTGGG

Energy 10: -23.855000: Start=1738 S1=7 S2=6 L1=2 L2=15 L3=0 End=1780

                    CTGCCGGGGCCTGAC       CC

                    GGGGTGG    TACTTC

CACTGGAGGTGGACGGAGCCCCCTGCC    GTGGAGCC

Energy 11: -23.625000: Start=7677 S1=5 S2=6 L1=1 L2=8 L3=0 End=7707

                    CTTGGAGA       G
```

```
                    CTCGA    CTCGGG
CCTGGGGTGGAGGTGCACACGGGCT    GGGCCTGC

Energy 12: -23.345000: Start=8164 S1=5 S2=6 L1=2 L2=23 L3=0 End=8210
                    GGCGGTCAGGGCAGGGGTCTGTG       AC
                    TGTCC    GGAGGT
CAGCAGATAGGTCCCGAGCAGCAGG    TCTCCACC

Energy 13: -23.085000: Start=7580 S1=5 S2=6 L1=1 L2=16 L3=0 End=7618
                    GCCTTGGGGTGCAGAA       C
                    TGGAA    GGGACC
TTTCTGAGTCTTGTACTGGGACCTT    TCCTGGGA

Energy 14: -22.695000: Start=5245 S1=6 S2=6 L1=1 L2=17 L3=0 End=5286
                    ATCCACAGCAGAGGGGA       G
                    GTTCGA    GTCGAG
GAGAAGGAGAAGATCCTGGACAAGCT    CAGTTCCG

Energy 15: -22.385000: Start=3597 S1=5 S2=7 L1=2 L2=28 L3=0 End=3650
                    CATCAACCTGGCCACGGCGGACACTGTC       GC
                    GGGTC    TGGTGGA
GTTCTGCTTCCTCCTCTCAACCCGG    ATCATCTAC

Energy 16: -21.595000: Start=7015 S1=5 S2=6 L1=1 L2=35 L3=0 End=7072
                    GGAGCCACTGCTGCGTTGGGTTTGCCTTCTGCCAG       A
                    CTCCG    TTCACG
AGGGAGGGTGGGCTGGGGGGGGGGC    AGGTGTGG

Time taken to run: 0 seconds 0 milliseconds
-------------------------------------------------
```

2) Output file for NM_001008392, CTDSPL mRNA

```
Sequence #: NM_001008392
Start=321 End=1151
loaded 4753 nucleotides, cds_start 321 to cds_end 1151

Energy 1: -38.900000: Start=275 S1=9 S2=7 L1=1 L2=28 L3=0 End=335

                    GGCCGCCGCGCCGCGCACCCATGGACGG       G

                    CGGGCGTCC    GGGCCGG

GCCGCGCCCCCGCGCGCTTGGCTTGCGGG    CCCGGCCAT

Found #1 stem loop within loop2: S3=3 AL=6 L2_tail=16 loop2(301-328)
cds_start=321

(GGC)CGCCGC(GCC)GCGCACCCATGGACGG


Energy 2: -30.205000: Start=74 S1=6 S2=6 L1=2 L2=19 L3=0 End=118

                    TTCATGGTGACGAGGCGGC       GC

                    GGGGCC    TCGGCG

CGCCTCCCCGTGCGCGGCTCTCCCGG    GGCCGCTC


Energy 3: -25.415000: Start=1219 S1=6 S2=6 L1=1 L2=19 L3=0 End=1262

                    AAGTGAGGATACTCCGTGC       C

                    AGTCGA    GGGTCC

CTCAGGGGACCTGCCTGTCCTCAGCT    TCCAGGCC


Energy 4: -22.270000: Start=2572 S1=5 S2=6 L1=2 L2=18 L3=0 End=2613

                    ACAGGGAGAGCCCAGGCT       AG

                    CCCTC    GAGAAG

GGACTCATTGCCCCAAACCAGGGAG    CTCTTTGC
```

```
Energy 5: -21.180000: Start=2591 S1=8 S2=6 L1=2 L2=12 L3=0 End=2632

                    CAGCCTTTCCCA       GC

                    GTTTCTCT    CGGACC

AGGGAGAGGAAGAGCTCCCACAGGGAGA    GTTTGGTG

Time taken to run: 0 seconds 0 milliseconds
-------------------------------------------------
```

3) Output file for NM_007246, KLHL2 mRNA

```
Sequence #: NM_007246
Start=317 End=2098
loaded 3185 nucleotides, cds_start 317 to cds_end 2098

Energy 1: -26.130000: Start=234 S1=6 S2=7 L1=2 L2=25 L3=0 End=286

                    GGCTGGAATGGTGCTGGCTGTGTTG       AG

                    CGCCTG    CGGCCGT

GATGGAACGCGGCTCGGCGGGCGGGC    GTCGGTGCC

Found #1 stem loop within loop2: S3=6 AL=7 L2_tail=6 loop2(255-278)
cds_start=317
(GGCTGG)AATGGTG(CTGGCT)GTGTTG


Energy 2: -23.000000: Start=499 S1=8 S2=7 L1=2 L2=32 L3=0 End=562

                    CATAGAGTGGTGCTGGCCGCCTGTAGTCCTTA       AC

                    TCGTCTTT    AAAGGTA

CTGTGCGATGTCACAATTGTGGCAGAAG    TTTTCATGC

Time taken to run: 0 seconds 0 milliseconds
-------------------------------------------------
```

4) Output file for NM_022733, SMAP2 mRNA

```
Sequence #: NM_022733
Start=395 End=1684
loaded 2905 nucleotides, cds_start 395 to cds_end 1684

Energy 1: -25.565000: Start=1487 S1=5 S2=6 L1=1 L2=32 L3=0 End=1541
```

```
                    CCAGACTGTGTATGGGGTCCAGCCAGCTCAGC       G

                    CCGTA    TCGACG

AGCAGGCTGGCTACATGGCAGGCAT    AGCTGCAA


Energy 2: -24.005000: Start=263 S1=5 S2=6 L1=1 L2=35 L3=0 End=320

                    CCGCTCAGGAGGTGCCCCTGGGCGGGGGACCGGGA       A

                    CCTCT    CGGGAG

AAGGGGCGTCCGGCGGGGCCGGAGG    GTCCTCAA

Found #1 stem loop within loop2: S3=9 AL=7 L2_tail=10 loop2(280-313)
cds_start=395
(CCGCTCAGG)AGGTGCC(CCTGGGCGG)GGGACCGGGA


Energy 3: -22.075000: Start=327 S1=5 S2=6 L1=2 L2=26 L3=0 End=376

                    CTGGGGGCGAGGAGGGCGCGTCGCCC       GA

                    TCTGG    GGACGG

GACCGGGAGTCCTCAACCCCGGACT    TCTGCCCC


Energy 4: -18.725000: Start=112 S1=7 S2=6 L1=2 L2=30 L3=0 End=169

                    AGGGTCTCTGGCCCCGCAGCCTCTCTTGGA       CC

                    GTCGGGT    CCGTTT

TCCGGCACCCAGGAGGCTCGTGGTCCG    GGCGGGCC

Time taken to run: 0 seconds 0 milliseconds
-------------------------------------------------
```

5) Output file for NM_001009552, PPP2CB mRNA

```
Sequence #: NM_001009552
Start=398 End=1327
loaded 1946 nucleotides, cds_start 398 to cds_end 1327

Energy 1: -29.270000: Start=224 S1=5 S2=7 L1=1 L2=26 L3=0 End=274

                    GGCCGCCCTACCCGGCTCAGTCCTCC       G
```

```
                    CGCCC    GGGGCGC

GAGCCGCCTGCTGGGCTTGGGCGGG    CCCTGTGGG

Found #1 stem loop within loop2: S3=5 AL=7 L2_tail=9 loop2(242-266)
cds_start=398
(GGCCG)CCCTACC(CGGCT)CAGTCCTCC

Energy 2: -26.155000: Start=207 S1=7 S2=6 L1=1 L2=27 L3=0 End=260

                    GGCGCGGGGCCCGCGGCCGCCCTACCC       C

                    GGCGGGT    TCGGGT

TCCAGGCTGCGCGCTCGGAGCCGCCTG    GGCTCAGT


Energy 3: -25.610000: Start=987 S1=14 S2=6 L1=2 L2=32 L3=0 End=1060

                    GGGGTATTTCACCACGTGGTGCTGGCTACACA       GA

                    TAGGTGGTGCTAGT    AGACCT

ACATGAGGGCCCAATGTGTGATCTGTTATGGTCA    TTTGGACA

Time taken to run: 0 seconds 0 milliseconds
-------------------------------------------------
```

6) Output file for NM_001282921, MAB21L4 mRNA

```
Sequence #: NM_001282921
Start=97 End=993
loaded 2015 nucleotides, cds_start 97 to cds_end 993

Energy 1: -29.320000: Start=569 S1=5 S2=7 L1=2 L2=15 L3=0 End=609

                    CTGGGCAGAACTGCA       CT

                    AGGAG    CCCGCGG

GCTGCTGTGGGCCTCTGTGCTCTTC    GGGCGCCGT


Energy 2: -27.905000: Start=24 S1=8 S2=6 L1=2 L2=31 L3=0 End=84

                    CCATTGGGCAGGCATGGTGGCCAGCTCCTCA       GT

                    ACTGTCAG    GAGCCG
```

```
GGTGCTGCAAGTCGCTGAGCTGACAGTT    CTTGGCTG

Found #1 stem loop within loop2: S3=6 AL=8 L2_tail=11 loop2(48-77)
cds_start=97
(CCATTG)GGCAGGCA(TGGTGG)CCAGCTCCTCA


Energy 3: -26.760000: Start=246 S1=5 S2=6 L1=1 L2=16 L3=0 End=284

                    TGAGAAGGAAGCTTGG       T

                    GGTGC    CCGTGG

GGCTGGAGAACAATCAGCTTCCACG    GGCGCCTG


Energy 4: -24.120000: Start=424 S1=5 S2=6 L1=1 L2=11 L3=0 End=457

                    GCAGGGTCATC       G

                    TCCCT    CGGGTC

ACCTGCTCACGAGGCTGCTGGGGGA    GCCTGGAC


Energy 5: -23.850000: Start=1793 S1=7 S2=7 L1=2 L2=7 L3=0 End=1829

                    TGAGTTC       TT

                    GAGTGAT    AGTACTC

TCATGGGGGTGGATTTCTCCCTTACTG    TCATGAGAT


Energy 6: -22.615000: Start=63 S1=5 S2=7 L1=1 L2=32 L3=0 End=119

                    TGGCTGAGGTCCAGGGATGGACACAGGTGTCT       C

                    TCACT    CCTCGAC

TGTCACCATTGGGCAGGCATGGTGG    GGGGTTGGG


Energy 7: -20.610000: Start=1377 S1=5 S2=7 L1=1 L2=21 L3=0 End=1422

                    GAAAGGGTACTGTCCACTTTT       G

                    ACGAC    ACATATG

TGGTTTTGAAAAAATGAATGTGCTG    TGTATATCA

Time taken to run: 0 seconds 0 milliseconds
```

----

7) Output file for NM_001348255, SMIM10L2B mRNA

```
Sequence #: NM_001348255
Start=80 End=316
loaded 2817 nucleotides, cds_start 80 to cds_end 316

Energy 1: -32.790000: Start=57 S1=7 S2=7 L1=2 L2=14 L3=0 End=100

                    ATGGCGGCGTCGGC       CG

                    CCCGGGC    CCGGGGC

TGCGGGTCGATCGGTCGGCGGGGCCTG    GGCTCTGTC


Energy 2: -32.090000: Start=433 S1=5 S2=6 L1=1 L2=21 L3=0 End=476

                    TGGACTCTAGACCTACGCCGC       G

                    CCCCG    GGCCCG

TCGCGGCGGGGGGCGGAGGTGGGGC    CCGGGCAC


Energy 3: -28.545000: Start=5 S1=6 S2=7 L1=1 L2=27 L3=0 End=58

                    GGCTCGAGCTCTTGCGGGTCGATCGGT       T

                    AGTCCT    GCCGCCC

                AGTGTTGGGG    CGGCGGGGC

Found #1 stem loop within loop2: S3=6 AL=8 L2_tail=7 loop2(25-50)
cds_start=80
(GGCTCG)AGCTCTTG(CGGGTC)GATCGGT


Energy 4: -26.940000: Start=2082 S1=6 S2=7 L1=1 L2=20 L3=0 End=2128

                    GTGTCTGATGCACAGATGTG       T

                    TTCCTC    CGGGGAG

GATACAGAGCTGGAGGAGAAGAGGAG    GCCCTTTCA


Energy 5: -26.100000: Start=112 S1=5 S2=7 L1=1 L2=14 L3=0 End=150
```

```
                    TGGCTTGGCGGTTC       C

                    CTGTC    CCGACGG
GCGGCTCTGTCTGCGGCGGCGGCGG    GGCTGTCGC


Energy 6: -24.825000: Start=1583 S1=6 S2=6 L1=2 L2=15 L3=0 End=1623

                    TGTACAATGCTCAAG       GT

                    GGTCAA    TCGTCG

TGGATTGGTCCTGGGGGCCCCCGGTT    AGCAGCCC


Energy 7: -21.545000: Start=1328 S1=5 S2=7 L1=1 L2=30 L3=0 End=1382

                    TTCAGCAGTGAAGTGGCACAAACCTCCCAG       T

                    TTCGG    GAACCAC

TAGCCTCCCCACCCCACCTGGAGCT    TTTGGTGGG


Energy 8: -20.645000: Start=1711 S1=5 S2=6 L1=1 L2=22 L3=0 End=1755

                    AAACTGCAGCAGTCTGGCCAGC       T

                    AGTTA    GGGTCC

GTACCCTGAGGGCCACATTCTCAGT    TCCAGGGA


Energy 9: -20.210000: Start=2169 S1=5 S2=6 L1=1 L2=8 L3=0 End=2199

                    CCAGCCAG       C

                    GGGTC    CTTCTC

TGGGTGACTGGATCCCTAGCTCCAG    GAAGGGTG

Time taken to run: 0 seconds 0 milliseconds
-------------------------------------------------
```

8) Output file for NM_001376852, TMEM181 mRNA

```
Sequence #: NM_001376852
Start=135 End=1562
```

```
loaded 5103 nucleotides, cds_start 135 to cds_end 1562

Energy 1: -29.185000: Start=71 S1=6 S2=7 L1=2 L2=34 L3=0 End=132

                    GCTCGGGACGCGCGGGCCGGGGCCGAGGGCTCTG       CG

                    GGCGGT    CCGCGGC

TGCGGCTGCCGCTGCCGAGGCTGCTG    GGCGCCGAG

Found #1 stem loop within loop2: S3=5 AL=7 L2_tail=17 loop2(92-124)
cds_start=135
(GCTCG)GGACGCG(CGGGC)CGGGGCCGAGGGCTCTG


Energy 2: -22.180000: Start=2020 S1=5 S2=6 L1=2 L2=22 L3=0 End=2065

                    CTTCCTCGTTGAGTGGCCAGTG       CT

                    CTGAC    GGGACT

CAAAGGGAAGCCTTATCTGTGGCTG    CCCTGGGT

Time taken to run: 0 seconds 0 milliseconds
```

9) Output file for XM_027630882, MRET_4255 mRNA

```
Sequence #: XM_027630882
Start=1 End=1116
loaded 1116 nucleotides, cds_start 1 to cds_end 1116

Energy 1: -30.325000: Start=6 S1=9 S2=6 L1=2 L2=26 L3=0 End=63

                    CTGTGGCTCTGTACGGGTATCAGCAT       AG

                    GTGTCCCTG    TGCGGC

               ATGTCCGTAGGGAC    GCGCCGAC

Found #1 stem loop within loop2: S3=5 AL=6 L2_tail=10 loop2(32-56)
cds_start=1
(CTGTG)GCTCTG(TACGG)GTATCAGCAT


Energy 2: -21.815000: Start=26 S1=7 S2=7 L1=2 L2=29 L3=0 End=84

                    TATCAGCATGCGCCGACCGAGGCACTGAC       TG

                    GGGCATG    TCTCGGT
```

```
CGTAGGGACAGCGGCGTGTCCCTGTGC    GGAGTTGTA

Time taken to run: 0 seconds 0 milliseconds
-------------------------------------------------
```

10) Output file for NM_001037, SCN1B mRNA

```
Sequence #: NM_001037
Start=352 End=1008
loaded 1666 nucleotides, cds_start 352 to cds_end 1008

Energy 1: -27.790000: Start=346 S1=6 S2=6 L1=1 L2=29 L3=0 End=399

                    TGGCCTTAGTGGTCGGCGCGGCACTGGTG       A

                    CGTCGG    AGGGGT

GGGGGCGCAGCACGCGCCGCGCAGCC    TCCTCAGC

Found #1 stem loop within loop2: S3=5 AL=5 L2_tail=14 loop2(365-392)
cds_start=352
(TGGCC)TTAGT(GGTCG)GCGCGGCACTGGTG


Energy 2: -26.310000: Start=76 S1=5 S2=7 L1=1 L2=20 L3=0 End=120

                    CCAGCGCAGCAGCCCGAGCA       C

                    GCGCG    CGTCGGC

GGGTCGGTGCACCTAGCGGATGTGC    GCGGCCGCC


Energy 3: -25.020000: Start=76 S1=5 S2=6 L1=2 L2=8 L3=0 End=107

                    CCAGCGCA       CC

                    GCGCG    CGTCGG

GGGTCGGTGCACCTAGCGGATGTGC    GCAGCCCG


Energy 4: -24.605000: Start=1297 S1=6 S2=6 L1=1 L2=11 L3=0 End=1332

                    ACCCCTCCTCC       G

                    CGCCCC    GGCGAC

TTGGGGAGGGGGGCGGTGAGGTGGGG    TTGCTGAT
```

```
Energy 5: -22.575000: Start=1273 S1=6 S2=7 L1=1 L2=25 L3=0 End=1324

                    TGAGGTGGGGGCAGCGGCCCCGCAC       G

                    GGCGGG    GGGAGGG

CCCCACCCTGCTTCTGCTGCCTGTTT    CCCTCCTCC

Time taken to run: 0 seconds 0 milliseconds
-------------------------------------------------
```

11) Output file for NM_004332, BPHL mRNA

```
Sequence #: NM_004332
Start=31 End=906
loaded 1909 nucleotides, cds_start 31 to cds_end 906

Energy 1: -26.270000: Start=35 S1=6 S2=6 L1=1 L2=27 L3=0 End=86

                    GGGCGTGTTGCGCCTGCGGCTGCTTCT       T

                    GCCGGC    GGGTCG

TACGCGACCTGTGACCATGGTGGCTG    CTCAGCGC

Found #1 stem loop within loop2: S3=5 AL=5 L2_tail=12 loop2(54-79)
cds_start=31
(GGGCG)TGTTG(CGCCT)GCGGCTGCTTCT


Energy 2: -20.535000: Start=218 S1=5 S2=7 L1=1 L2=5 L3=0 End=247

                    TGTTA       C

                    AGGGT    CCTTCAT

TGGAGAGGGAGATCACGCAGTCCTG    GGAAGTGGA


Energy 3: -20.265000: Start=929 S1=6 S2=6 L1=1 L2=21 L3=0 End=974

                    TGATCGTGTTGCTGCCTGTTA       T

                    TCGGGG    TGTGCT

ATGCACACTCCAGTCTTGGTGGTTCC    ACATGATG

Time taken to run: 0 seconds 0 milliseconds
-------------------------------------------------
```

12) Output file for NM_005376, MYCL mRNA

```
Sequence #: NM_005376
Start=30 End=740
loaded 1942 nucleotides, cds_start 30 to cds_end 740

Energy 1: -27.405000: Start=11 S1=5 S2=7 L1=1 L2=19 L3=0 End=54

                    CATGTGCGTGTGTGCTGGC       T

                    GCGCG    GCGGCTC

          GAGTGCGGGCCGCGC    TGCCGGGCT

Found #1 stem loop within loop2: S3=5 AL=4 L2_tail=5 loop2(29-46)
cds_start=30
(CATGT)GCGT(GTGTG)CTGGC


Energy 2: -22.795000: Start=11 S1=5 S2=6 L1=2 L2=12 L3=0 End=46

                    CATGTGCGTGTG       TC

                    GCGCG    GCGGCT

          GAGTGCGGGCCGCGC    TGCTGGCT


Energy 3: -22.500000: Start=1481 S1=5 S2=7 L1=2 L2=24 L3=0 End=1530

                    GAACTCCAGGCCCTCCTCTCCCAT       GG

                    ATGAC    GGAACTG

GTGGCAATCTGTGGGCTACATGCTG    CCTTGATGC


Energy 4: -21.215000: Start=251 S1=5 S2=6 L1=2 L2=8 L3=0 End=282

                    CGCAGGGG       GC

                    GGCCC    TGGGTT

TCGCCCCCCACGTCGCCGCCCTGGG    ACCCGGCC

Time taken to run: 0 seconds 0 milliseconds
-------------------------------------------------
```

13) Output file for NM_012144, DNAI1 mRNA

```
Sequence #: NM_012144
Start=202 End=2301
loaded 2529 nucleotides, cds_start 202 to cds_end 2301

Energy 1: -23.320000: Start=170 S1=6 S2=7 L1=2 L2=24 L3=0 End=221

                    CAGGGGTTGAGATGATTCCTGCTT       GT

                    CTCTCG    GATGTTT

AACTTTTTGTCTTCAGACGAGGGAGC    CTGCGAAGG

Found #1 stem loop within loop2: S3=8 AL=5 L2_tail=3 loop2(191-213)
cds_start=202
(CAGGGGTT)GAGAT(GATTCCTG)CTT


Energy 2: -22.000000: Start=1685 S1=5 S2=6 L1=2 L2=10 L3=0 End=1718

                    CCTTTGACTT       GG

                    GTCAC    GGTGTT

TGAGGGGTTGCAGCTGCACCCAGTG    CCACAAAG

Time taken to run: 0 seconds 0 milliseconds
```

14) Output file for NM_004787, SLIT2 mRNA

```
Sequence #: NM_004787
Start=1912 End=6501
loaded 8053 nucleotides, cds_start 1912 to cds_end 6501

Energy 1: -28.635000: Start=1916 S1=8 S2=7 L1=1 L2=18 L3=0 End=1964

                    CCCTGTCGCTGGGGTTAG       G

                    TGTCGTAG    ACGGTCG

GAGGCGGCGGGGAAAGATGCGCGGCGTT    TGCTGGCGA

Found #1 stem loop within loop2: S3=5 AL=4 L2_tail=4 loop2(1940-1956)
cds_start=1912
(CCCTG)TCGC(TGGGG)TTAG
```

Energy 2: -22.050000: Start=2416 S1=5 S2=6 L1=1 L2=6 L3=0 End=2444

```
                    CCGGGA       A

                    TCTCG    GGACTT

AGATCAGCTGTATTGAAGATGGGGC    CCTGGAAG
```

Time taken to run: 0 seconds 0 milliseconds
