# Supplementary material for: Possible involvement of three-stemmed pseudoknots in regulating translational initiation in human mRNAs
Source: PLoS One. 2024 Jul 22;19(7):e0307541. doi: 10.1371/journal.pone.0307541 (PMC11262651; doi:10.1371/journal.pone.0307541)
Supplement: S2 File — (PDF) [file pone.0307541.s002.pdf]

This document includes the sequence analysis (BLASTN) results for the 14 mRNAs where three-stemmed pseudoknots were detected near the AUG start codon and subsequently discussed in the manuscript

The accession ID and encoded protein of these nine mRNAs are:

NM_015557, chromodomain helicase DNA binding protein 5 (CHD5);
NM_001008392, CTD small phosphatase like (CTDSPL), transcript variant 1;
NM_007246, kelch like family member 2 (KLHL2), transcript variant 1;
NM_022733, small ArfGAP2 (SMAP2), transcript variant 1;
NM_001009552, protein phosphatase 2 catalytic subunit beta (PPP2CB);
NM_001282921, mab-21 like 4 (MAB21L4), transcript variant 3;
NM_001348255, small integral membrane protein 10 like 2B (SMIM10L2B);
NM_001376852, transmembrane protein 181 (TMEM181), transcript variant 4;
XM_027630882, dynactin 4 (MRET_4255), partial mRNA;
NM_001037, sodium voltage-gated channel beta subunit 1 (SCN1B);
NM_004332, biphenyl hydrolase like (BPHL), transcript variant 1
NM_005376, MYCL proto-oncogene, bHLH transcription factor (MYCL), transcript variant 3;
NM_012144, dynein axonemal intermediate chain 1 (DNAI1), transcript variant 1;
NM_004787, slit guidance ligand 2 (SLIT2), transcript variant 1.

In the BLASTN search, the pseudoknot forming sequence is used as the query. In the linear sequence, the three stems are highlighted by different background colors:

Cyan: stem1
Green: stem2
Grey: stem3

Letter in red indicates a position that has different nucleotides.

For example, in the pseudoknot forming sequence within the CHD5 mRNA:

```
QUERY  1    GGGAGGGGGGGCGCCCACCTCCCCTCCTCCCCGCGCCGGGCATGCGGGGCCCAGTGGGC  59
            ||||||||||||||||||||||||||||||||||||||||||||||||||||| |||||
SBJCT  114  GGGAGGGGGGGCGCCCACCTCCCCTCCTCCCCGCGCCGGGCATGCGGGGCCCACTGGGC  172
```

The following presents the sequence analysis results for the 9 mRNAs.

1) NM_015557, CHD5 mRNA

60 BLASTN hits, all from primates. 10 Homo sapiens and Pan paniscus sequences are 100% identical.

Sequences that are not 100% identical are examined in the following manner:

Trachypithecus francoisi chromodomain helicase DNA binding protein 5 (CHD5), mRNA
Sequence ID: XM_033228610.1

```
QUERY   1    GGGAGGGGGGGCGCCCACCTCCCCTCCTCCCCGCGCCGGGCATGCGGGGCCCAGTGGGC   59
             ||||||||||||||||||||||||||||||||||||||||||||||||||||| |||||
SBJCT   114  GGGAGGGGGGGCGCCCACCTCCCCTCCTCCCCGCGCCGGGCATGCGGGGCCCACTGGGC   172
```

Note: the **C** may go to loop2, and the loop1 G may extend stem1 by one basepair, stem2 has five basepairs. There are 27 identical sequences as the SBJCT sequence.

Symphalangus syndactylus chromodomain helicase DNA binding protein 5 (CHD5), transcript variant X1, mRNA
Sequence ID: XM_055262358.1

```
QUERY   1    GGGAGGGGGGGCGCCCACCTCCCCTCCTCCCCGCGCCGGGCATGCGGGGCCCAGTGGGC   59
             ||||||||||||||||||||||||||||||||||||||||||||||||||||| |||||
SBJCT   189  GGGAGGGGGGGCGCCCACCTCCCCTCCTCCCCGCGCCGGGCATGCGGGGCCCATTGGGC   247
```

Note: the **T** may go to loop2, and the loop1 G may extend stem1 by one basepair, stem2 has five basepairs. There are 4 identical sequences as the SBJCT sequence.

Pongo abelii chromodomain helicase DNA binding protein 5 (CHD5), transcript variant X5, mRNA
Sequence ID: XM_054542136.1

```
QUERY   1    GGGA-GGGGGGGCGCCCACCTCCCCTCCTCCCCGCGCCGGGCATGCGGGGCCCAGTGGGC   59
             |||| |||||||||||||||||||||||||||||||||||||||||||||||||| |||||
SBJCT   85   GGGAGGGGGGGGCGCCCACCTCCCCTCCTCCCCGCGCCGGGCATGCGGGGCCCACTGGGC   144
```

Note: There are 11 identical sequences as the SBJCT sequence.

Microcebus murinus chromodomain helicase DNA binding protein 5 (CHD5), transcript variant X1, mRNA
Sequence ID: XM_012791554.2

```
QUERY   1    GGGAGGGGGGGCGCCCACCTCCCCTCCTCCCCGCGCCGGGCATGCGGGGCCCAGTGGGC   59
             ||||||||||||||||||||||||||||||||||||||||||||||||||||   ||||
SBJCT   95   GGGAGGGGGGGCGCCCACCTCCCCTCCTCCCCGCGCCGGGCATGCGGGGCCCGCCGGGC   153
```

Note: There are 2 identical sequences as the SBJCT sequence.

Nycticebus coucang chromodomain helicase DNA binding protein 5 (CHD5), transcript variant X6, mRNA
Sequence ID: XM_053576559.1

```
QUERY   1     GGGAGGGGGGGCGCCCACCTCCCCTCCTCCCCGCGCCGGGCATGCGGGGCCCAGTGGGC   59
              |||| ||||| |||||||||||||||||||||||||||||||||||||||||  |||||
SBJCT   83    GGGA-GGGGGCCGCCCACCTCCCCTCCTCCCCGCGCCGGGCATGCGGGGCCCGCTGGGC   140
```

Nycticebus coucang chromodomain helicase DNA binding protein 5 (CHD5), transcript variant X5, mRNA
Sequence ID: XM_053576558.1

```
QUERY   1     GGGAGGGGGGGCGCCCACCTCCCCTCCTCCCCGCGCCGGGCATGCGGGGCCCAGTGGGC   59
              |||| ||||| |||||||||||||||||||||||||||||||||||||||||  |||||
SBJCT   256   GGGA-GGGGGCCGCCCACCTCCCCTCCTCCCCGCGCCGGGCATGCGGGGCCCGCTGGGC   313
```

Note: There are 5 identical sequences as the SBJCT sequence

2) NM_001008392, CTDSPL mRNA

238 BLASTN hits, wide range of species. 26 sequences are 100% identical.

Sequences that are not 100% identical are examined in the following manner:

Rhinopithecus roxellana CTD small phosphatase like (CTDSPL), transcript variant X2, mRNA
Sequence ID: XM_010357266.2

```
Query  1    GCTTGCGGGGGGCCGGGCCTGCGGGCGGCCGCCGCGCCGCGCACCCATGGACGGCCCGGCC  61
            |||||||||||||||||||||||||||||||||||||||| ||||||||||||||||||||
Sbjct  272  GCTTGCGGGGGGCCGGGCCTGCGGGCGGCCGCCGCGCCGCTCACCCATGGACGGCCCGGCC  332

Note: 5 identical sequences. Position with different nucleotide is in loop.
```

Marmota monax CTD small phosphatase like (Ctdspl), transcript variant X4, mRNA
Sequence ID: XM_058576765.1

```
Query  1   GCTTGCGGGGGGCCGGGCCTGCGGGCGGCCGCCGCGCCGCGCACCCATGGACGGCCCGGCC  61
           |||||||||||||||||||||||||||||||||| ||||||||||||||||||||||||||
Sbjct  28  GCTTGCGGGGGGCCGGGCCTGCGGGCGGCCGCCGTGCCGCGCACCCATGGACGGCCCGGCC  88


Note: 4 identical sequences. Position with different nucleotide is in loop.
```

Hylobates moloch CTD small phosphatase like (CTDSPL), transcript variant X2, mRNA
Sequence ID: XM_032162083.2

```
Query  1    GCTTGCGGGGGGCCGGGCCTGCGGGCGGCCGCCGCGCCGCGCACCCATGGACGGCCCGGCC  61
            |||||||||||||||||||||||||||||||||| ||||||||||||||||||||||||||
Sbjct  258  GCTTGCGGGGGGCCGGGCCTGCGGGCGGCCGCCGAGCCGCGCACCCATGGACGGCCCGGCC  318

Note: 7 identical sequences. Position with different nucleotide is in loop.
```

Nyctereutes procyonoides CTD small phosphatase like (CTDSPL), transcript variant X2, mRNA
Sequence ID: XM_055321164.1

```
Query  1   GCTTGCGGGGGGCCGGGCCTGCGGGCGGCCGCCGCGCCGCGCACCCATGGACGGCCCGGCC  61
           ||||||||||||||||||| |||||||||||||||||||||||||||||||||||||||||
Sbjct  16  GCTTGCGGGGGGCCGGGCCCGCGGGCGGCCGCCGCGCCGCGCACCCATGGACGGCCCGGCC  76
```

Note: 10 identical sequences. G-**U** to G-**C** in stem 1.

Pongo abelii CTD small phosphatase like (CTDSPL), transcript variant X7, mRNA
Sequence ID: XM_054552330.1

```
Query  1   GCTTGCGGGGGGCCGGGCCTGCGGGCGGCCGCCGCGCCGCGCACCCATGGACGGCCCGGCC  61
           |||||||||||||||||||||||||||||||||||||||||||| ||||||||||||||||
Sbjct  24  GCTTGCGGGGGGCCGGGCCTGCGGGCGGCCGCCGCGCCGCGCACTCATGGACGGCCCGGCC  84
```

Note: 6 identical sequences. Position with different nucleotide is in loop.

Mirounga angustirostris CTD small phosphatase like (CTDSPL), transcript variant X5, mRNA
Sequence ID: XM_045904047.2

```
Query  1   GCTTGCGGGGGGCCGGGCCTGCGGGCGGCCGCCGCGCCGCGCACCCATGGACGGCCCGGCC  61
           ||||||||||||||||||| |||||||||||||||||||||||||||||||||||||||||
Sbjct  28  GCTTGCGGGGGGCCGGGCCGGCGGGCGGCCGCCGCGCCGCGCACCCATGGACGGCCCGGCC  88
```

Note: 10 identical sequences. G-**U** to G-**G** in stem 1.

Sciurus carolinensis CTD small phosphatase like (Ctdspl), transcript variant X2, mRNA
Sequence ID: XM_047531008.1

```
Query  1    GCTTGCGGGGGGCCGGGCCTGCGGGCGGCCGCCGCGCCGCGCACCCATGGACGGCCCGGCC  61
            ||||||||||||||||||||||||||| |||||||||||||||||||||||||||||||||
Sbjct  342  GCTTGCGGGGGGCCGGGCCTGCGGGCGACCGCCGCGCCGCGCACCCATGGACGGCCCGGCC  402
```

Note: 2 identical sequences. Mismatch in stem3.

Lemur catta CTD small phosphatase like (CTDSPL), mRNA
Sequence ID: XM_045536727.1

```
Query  1    GCTTGCGGGGGGCCGGGCCTGCGGGCGGCCGCCGCGCCGCGCACCCATGGACGGCCCGGCC  61
            ||||||||||||||||||||||||||||||||||||||| |||||||||||||||||||||
Sbjct  180  GCTTGCGGGGGGCCGGGCCTGCGGGCGGCCGCCGCGCCGTGCACCCATGGACGGCCCGGCC  240
```

Note: 1 identical sequences. In loop. Position with different nucleotide is in loop.

Choloepus didactylus CTD small phosphatase like (CTDSPL), mRNA
Sequence ID: XM_037847344.1

```
Query  1    GCTTGCGGGGGGCCGGGCCTGCGGGCGGCCGCCGCGCCGCGCACCCATGGACGGCCCGGCC  61
```

```
               |||||||||||||||||||||||||||||||||||||| ||||||||||||||||||||||
Sbjct  295     GCTTGCGGGGGGCCGGGCCTGCGGGCGGCCGCCGCGCCACGCACCCATGGACGGCCCGGCC  355
```

Note: Position with different nucleotide is in loop.

Octodon degus CTD small phosphatase like (Ctdspl), transcript variant X2, mRNA
Sequence ID: [XM_004635242.2]

```
Query  1     GCTTGCGGGGGGCCGGGCCTGCGGGCGGCCGCCGCGCCGCGCACCCATGGACGGCCCGGCC  61
             |||||||||||||||||||||||||||||||| ||||||||||||||||||||||||||||
Sbjct  10    GCTTGCGGGGGCCGGGCCTGCGGGCGGCCGCTGCGCCGCGCACCCATGGACGGCCCGGCC  70
```

Note: Position with different nucleotide is in loop.

Chinchilla lanigera CTD (carboxy-terminal domain, RNA polymerase II, polypeptide A) small phosphatase-like (Ctdspl), mRNA
Sequence ID: [XM_005383711.2]

```
Query  1      GCTTGCGGGGGCCGGGCCTGCGGGCGGCCGCCGCGCCGCGCACCCATGGACGGCCCGGCC  60
              |||||||||||||||||||||||||||||||||||||||||||||||||||| ||||||| 
Sbjct  92     GCTTGCGGGGGCCGGGCCTGCGGGCGGCCGCCGCGCCGCGCACCCATGGACTGCCCGGCT  151
```

Note: Position with different nucleotide is in loop. G-C to G-U in stem2.

Pongo abelii CTD small phosphatase like (CTDSPL), transcript variant X5, mRNA
Sequence ID: [XM_024244516.2]

```
Query  3     TTGCGGGGGGCCGGGCCTGCGGGCGGCCGCCGCGCCGCGCACCCATGGACGGCCCGGCC  61
             |||||||||||||||||||||||||||||||||||||||||| ||||||||||||||||
Sbjct  1     TTGCGGGGGGCCGGGCCTGCGGGCGGCCGCCGCGCCGCGCACTCATGGACGGCCCGGCC  59
```

Note: 11 identical sequences. Position with different nucleotide is in loop

Rhinolophus ferrumequinum CTD small phosphatase like (CTDSPL), mRNA
Sequence ID: [XM_033131796.1]

```
Query  1      GCTTGCGGGGGGCCGGGCCTGCGGGCGGCCGCCGCGCCGCGCACCCATGGACGGCCCGGCC  61
              ||| ||||||||||||||| |||||||||||||||||||||||||||||||||||||||||
Sbjct  274    GCTGGCGGGGGGCCGGGCCGGCGGGCGGCCGCCGCGCCGCGCACCCATGGACGGCCCGGCC  334
```

Note: 2 mismatches in stem 1.

Mustela erminea CTD small phosphatase like (CTDSPL), mRNA
Sequence ID: [XM_032354052.1]

```
Query  1      GCTTGCGGGGGGCCGGGCCTGCGGGCGGCCGCCGCGCCGCGCACCCATGGACGGCCCGGCC  61
              ||||||||||||||||||| ||||| |||||||||||||||||||||||||||||||||||
Sbjct  655    GCTTGCGGGGGGCCGGGCCGGCGGGTGGCCGCCGCGCCGCGCACCCATGGACGGCCCGGCC  715
```

Note: 6 identical sequences. 1 mismatch AND G-**C** to G-**U** in stem 1.

Neofelis nebulosa CTD small phosphatase like (CTDSPL), transcript variant X5, mRNA
Sequence ID: [XM_058729402.1]

```
Query  1     GCTTGCGGGGGGCCGGGCCTGCGGGCGGCCGCCGCGCCGCGCACCCATGGACGGCCCGGCC  61
             ||||||||||||||||||| |||||||| ||||||||||||||||||||||||||||||||
Sbjct  82    GCTTGCGGGGGGCCGGGCCGGCGGGCGGACGCCGCGCCGCGCACCCATGGACGGCCCGGCC  142
```

Note: 31 identical sequences. 1 G-G mismatch in stem 1 AND bulge in stem 3.

Lutra lutra CTD small phosphatase like (CTDSPL), transcript variant X3, mRNA
Sequence ID: [XM_047740695.1]

```
Query  1     GCTTGCGGGGGGCCGGGCCTGCGGGCGGCCGCCGCGCCGCGCACCCATGGACGGCCCGGCC  61
             ||||||||||||||||||| ||||| |||||||||||||||||||||||||||||||||||
Sbjct  341   GCTTGCGGGGGGCCGGGCCAGCGGGTGGCCGCCGCGCCGCGCACCCATGGACGGCCCGGCC  401
```

Note: 3 identical sequences. 1 G-A mismatch AND G-C to G-U in in stem 1.

Chlorocebus sabaeus CTD small phosphatase like (CTDSPL), transcript variant X2, mRNA
Sequence ID: [XM_007971772.2]

```
Query  1     GCTTGC-GGGGGGCCGGGCCTGCGGGCGGCCGCCGCGCCGCGCACCCATGGACGGCCCGGCC  61
             |||||| ||||||||||||| |||||||||||||||||||||||||||||||||||||||||
Sbjct  320   GCTTGCGGGGGGGCCGGGCCAGCGGGCGGCCGCCGCGCCGCGCACCCATGGACGGCCCGGCC  381
```

Note: 2 identical sequences. Bulge in stem 1.

Bos indicus x Bos taurus CTD small phosphatase like (CTDSPL), transcript variant X3, mRNA
Sequence ID: [XM_027523695.1]

```
QUERY  1   GCTTGCGGGGGGCCGGGCCTGCGGGCGGCCGCCGCGCCGCGCACCCATGGACGGCCCGGCC  61
           |||   ||||||||||||| |||||||||||||||||||||||||||||||||||||||||
SBJCT  2   GCTGCCGGGGGGCCGGGCCGGCGGGCGGCCGCCGCGCCGCGCACCCATGGACGGCCCGGCC  62
```

Note: 14 identical sequences. Two bulges in stem 1

Equus caballus CTD small phosphatase like (CTDSPL), transcript variant X2, mRNA
Sequence ID: [XM_023620743.1]

```
Query  1   GCTTGCGGGGGGCCGGGCCTGCGGGCGGCCGCCGCGCCGCGCACCCATGGACGGCCCGGCC  61
           ||||||||||||||||||| | ||| |||||||||||||||||||||||||||||||||||
Sbjct  25  GCTTGCGGGGGGCCGGGCCCGTGGGTGGCCGCCGCGCCGCGCACCCATGGACGGCCCGGCC  85
```

Note: 2 identical sequences. Two pairs G-C to G-U AND one G-U to G-C in stem 1.

Sus scrofa CTD small phosphatase like (CTDSPL), transcript variant X4, mRNA
Sequence ID: [XM_005669358.3]

```
QUERY  1     GCTTGCGGGGGGCCGGGCCTGCGGGCGGCCGCCGCGCCGCGCACCCATGGACGGCCCGGCC  61
                   ||||||||||||| |||||||||||||||||||||||||||||||||||||||||
SBJCT  10  GGCTGCCGGGGGGGCCGGGCCGGCGGGCGGCCGCCGCGCCGCGCACCCATGGACGGCCCGGCC  72
```

Note: 12 identical sequences. Although the 5’-end sequences are totally different, an alternative stem 1 can still form.

Nycticebus coucang CTD small phosphatase like (CTDSPL), mRNA
Sequence ID: XM_053599715.1

```
Query  1    GCTTGCGGGGGGCCGGGCCTGCGGGCGGCCGCCGCGCCGCGCACCCATGGACGGCCCGGCC  61
            ||||||||||||||||||||||||||||||||||||||  |||||||||||| ||||||||
Sbjct  195  GCTTGCGGGGGGCCGGGCCTGCGGGCGGCCGCCGCGCCAAGCACCCATGGACAGCCCGGCC  255
```

Note: Position with different nucleotide is in loop

Manis javanica CTD small phosphatase like (CTDSPL), transcript variant X8, mRNA
Sequence ID: XM_017665240.2

```
QUERY  1         GCTTGCGGGGGGCCGGGCCTGCGGGCGGCCGCCGCGCCGCGCACCCATGGACGGCCCGGCC  61
                 || |||||||||||||||   ||||||||||||||||||||||||||||||||||||||||
SBJCT  19  CGCCTGGCCTGCGGGGGGCCGGGCGGGCGGGCGGCCGCCGCGCCGCGCACCCATGGACGGCCCGGCC  85
```

Note: 7 sequences. Alternative stem1 can still form.

Rousettus aegyptiacus CTD small phosphatase like (CTDSPL), transcript variant X5, mRNA
Sequence ID: XM_036219888.1

```
QUERY  1          GCTTGCGGGGGGCCGGGCCTGCGGGCGGCCGCCGCGCCGCGCACCCATGGACGGCCCGGCC  61
                  ||| |||||||||||||||   ||||||||||||||||||||||||||||||||||||||||
SBJCT  258  GCGTCTGGCTGGCGGGGGGCCGGGCGGGCGGGCGGCCGCCGCGCCGCGCACCCATGGACGGCCCGGCC  318
```

Note: 2 sequences. Alternative stem1 can still form.

Pteronotus parnellii mesoamericanus CTD small phosphatase like (CTDSPL), transcript variant X4, misc_RNA
Sequence ID: XR_008530969.1

```
Query  5  GCGGGGGGCCGGGCCTGCGGGCGGCCGCCGCGCCGCGCACCCATGGACGGCCCGGCC  61
          ||||||||||||||| | ||||||||||||||||||||||||||||||||||||||||
Sbjct  3  GCGGGGGGCCGGGCCGGTGGGCGGCCGCCGCGCCGCGCACCCATGGACGGCCCGGCC  59
```

Note: 4 sequences. Alternative stem1 and stem3 can still form.

Artibeus jamaicensis CTD small phosphatase like (CTDSPL), transcript variant X2, mRNA
Sequence ID: XM_037148278.2

```
QUERY  1        GCTTGGCGGGGGCCGGGCCTGCGGGCGGCCGCCGCGCCGCGCACCCATGGACGGCCCGGCC  61
                |   ||||||||||||||||| | ||||||||||||||||||||||||||||||||||||||
SBJCT  315  GCCTGGCCGGCGGGGGGCCGGGCCGGTGGGCGGCCGCCGCGCCGCGCACCCATGGACGGCCCGGCC  371
```

Note: 7 sequences. Alternative stem1 can still form.

Phyllostomus discolor CTD small phosphatase like (CTDSPL), transcript variant X2, mRNA
Sequence ID: XM_028518988.2

```
QUERY  1     GCTTGCGGGGGGCCGGGCCTGCGGGCGGCCGCCGCGCCGCGCACCCATGGACGGCCCGGCC   61
             ||  ||||||||||||||  | |||||||||||||||||||||||||||||||||||||||
SBJCT  21  TGGCCGGCGGGGGGCCGGGCGGGTGGGCGGCCGCCGCGCCGCGCACCCATGGACGGCCCGGCC   77
```

```
Note: 2 sequences. Mismatch in stem1.
```

Pteropus giganteus CTD small phosphatase like (CTDSPL), transcript variant X8, mRNA
Sequence ID: XM_039843319.1

```
QUERY  1     GCTTGCGGGGGGCCGGGCCTGCGGGCGGCCGCCGCGCCGCGCACCCATGGACGGCCCGGCC   61
             ||| ||  ||||||||||  |||||||||||||||||||||||||||||||||||||||||
SBJCT  37    GCTGGCCCGGGGCCGGGCGGGCGGGCGGCCGCCGCGCCGCGCACCCATGGACGGCCCGGCC   97
```

```
Note: 7 sequences. Covariation in stem 1, G-C/G-U to C-G/C-G.
```

Manis pentadactyla CTD small phosphatase like (CTDSPL), transcript variant X8, mRNA
Sequence ID: XM_036921617.2

```
Query  1      GCTTGCGGGGGGCCGGGCCTGCGGGC-GGCCGCCGCGCCGCGCACCCATGGACGGCCCGGCC   61   59
              || |||||||||||||||  || | | |||||||||||||||||||||||||||||||||||
Sbjct  89    GGCCTGCGGGGGGCCGGGC-GGCCGACTGGCCGCCGCGCCGCGCACCCATGGACGGCCCGGCC   150
```

```
Note: 8 sequences (variants). 3 mismatches in stem 1.
```

Callithrix jacchus CTD small phosphatase like (CTDSPL), transcript variant X2, mRNA
Sequence ID: XM_035278485.2

```
Query  1    GCTTGCGGGGGGCCGGGCCTGCGGGCGGCCGCCGCGCCGCGCACCCATGGACGGCCCGGCC   61
            ||||||||||||||||||||||||||||||| |||||||||||||||||||||||||||||
Sbjct  9    GCTTGCGGGGGGCCGGGCCTGCGGGCGGCCGGCGCGCCGCGCACCCATGGACGGCCCGGCC   69
```

```
Note: 2 sequences. Position with different nucleotide is in loop
```

Mastomys coucha CTD small phosphatase like (Ctdspl), transcript variant X1, mRNA
Sequence ID: XM_031345086.1

```
Query  1        GCTTGCGGGGGGCCGGGCCTGCGGGCGGCCGCCGCGCCGCGCACCCATGGACGGCCCGGCC   61
                ||||||||||||||||||||||||||||||| |||| ||||||||||||||||| ||||||
Sbjct  374    TAGCTTGCGGGGGGCCGGGCCTGCGGGCGGCCGGCGCGGCGCGCACCCATGGACGGTCCGGCC   436
```

```
Note: 6 sequences. Position with different nucleotide is in loop, and C-G to U-G, and
alternative stem1 and stem3.
```

Microtus ochrogaster CTD small phosphatase like (Ctdspl), transcript variant X2, mRNA
Sequence ID: XM_026779151.1

```
Query  1    GCTTGCGGGGGGCCGGGCCTGCGGGCGGCCGCCGCGCCGCGCACCCATGGACGGCCCGGCC   61
            ||||||||||||||||||||||||||||||| |||  ||||||||||||||||| ||||||
Sbjct  7    GCTTGCGGGGGGCCGGGCCTGCGGGCGGCCGGCGCTGCGCGCACCCATGGACGGTCCGGCC   67
```

Note: 15 sequences. In loop, and C-G to U-G, and alternative stem 3.

Meriones unguiculatus CTD small phosphatase like (Ctdspl), mRNA
Sequence ID: XM_021648030.1

```
Query  1       GCTTGCGGGGGGCCGGGCCTGCGGGCGGCCGCCGCGCCGCGCACCCATGGACGGCCCGGCC  61
               || |||||||||||||||||||||||||||| |||| |||||||||||||||| |||||
Sbjct  283   CAGCCTGCGGGGGGCCGGGCCTGCGGGCGGCCGGCGCGGCGCGCACCCATGGACGGTCCGGCC  343
```

Note: 4 sequences. Position with different nucleotide is in loop, and alternative stem 1 and stem3, and U-G to C-G stem1, and C-G to U-G stem2.

Apodemus sylvaticus CTD small phosphatase like (Ctdspl), transcript variant X2, mRNA
Sequence ID: XM_052187012.1

```
Query  1       GCTTGCGGGGGGCCGGGCCTGCGGGCGGCCGCCGCGCCGCGCACCCATGGACGGCCCGGCC  61
               |||||||||||||||||||||||||||| | |||| |||||||||||||||| ||||||
Sbjct  48    TAGCTTGCGGGGGGCCGGGCCTGCGGGCGGCTGGCGCGGCGCGCACCCATGGACGGTCCGGCC  108
```

Note: 1 sequences . Position with different nucleotide is in loop, and alternative stem1 and stem3.

Equus asinus CTD small phosphatase like (CTDSPL), transcript variant X4, mRNA
Sequence ID: XM_044755062.1

```
Query  1    GCTTGCGGGGGGCCGGGCCTGCGGGC-GGCCG-CCGCGCCGCGCACCCATGGACGGCCCGGCC  61
            ||||||||||||||||||| | |||  ||||| |||||||||||||||||||||||||||||
Sbjct  28   GCTTGCGGGGGGCCGGGCCCGTGGGGTGGCCGCCCGCGCCGCGCACCCATGGACGGCCCGGCC  90
```

Note: 2 sequences. Position with different nucleotide is in loop, and alternative stem1 and stem3.

Microtus oregoni CTD small phosphatase like (Ctdspl), transcript variant X2, mRNA
Sequence ID: XM_041677225.1

```
Query  1     GCTTGCGGGGGGCCGGGCCTGCGGGCGGCCGCCGCGCCGCGCACCCATGGACGGCCCGGCC  61
             |||||||||||||||||||||||||||||   |||  |||||||||||||||| ||||||
Sbjct  281   GCTTGCGGGGGGCCGGGCCTGCGGGCGGCCAGCGCTGCGCGCACCCATGGACGGTCCGGCC  341
```

Note: 2 sequences. Position with different nucleotide is in loop, and alternative stem3.

Sequence ID: XM_032459237.1

```
Query  1            GCTTGCGGGGGGCCGGGCCTGCGGGCGGCCGCCGCGCCGCGCACCCATGGACGGCCCGGCC  61
                    | || ||||||||||||||  | |||||| |||||||||||||||||||||||||||||
Sbjct  1411  CGCCTGGCTGCCGGGGGGCCGGGCCGCCCGGCGGCTGCCGCGCCGCGCACCCATGGACGGCCCGGCC  1477
```

Note: Position with different nucleotide is in loop, and alternative stem1 and stem3.

Camelus dromedarius CTD small phosphatase like (CTDSPL), transcript variant X4, mRNA

Sequence ID: XM_031469968.1

```
Query  1      GCTTGCGGGGGGCCGGGCCTGCGGGC-GGCCGCCGCGCCGCGCACCCATGGACGGCCCGGCC   61
              |  ||| |||||||  ||| ||  || ||| |||||||||||||||||||||||||||||||
Sbjct  241   TGGCTGCCGGGGGCC-CGCC-GCCCGCGGGCTGCCGCGCCGCGCACCCATGGACGGCCCGGCCATC 297
```

Note: 3 sequences. Position with different nucleotide is in loop, and alternative stems.

```
Query  1      GCTTGCGGGGGGCCGGGCCTGCGGGC-GGCCGCCGCGCCGCGCACCCATGGACGGCCCGGCC   61
              |  ||| |||||||  ||| ||  || ||  |||||||||||||||||||||||||||||||
Sbjct  241    GGCTGCCGGGGGCC-CGCC-GCCCGCGGC-TGCCGCGCCGCGCACCCATGGACGGCCCGGCC 297
```

Note: bulge and mismatch in stem1 and stem2.

3) XM_027630882, MRET_4255 mRNA

Three BLASTN hits, all identical.

4) NM_001376852, TMEM181 mRNA

25 BLASTN hits, all from primates. 13 sequences are 100% identical.

Sequences that are not 100% identical are examined in the following manner:

Gorilla gorilla gorilla transmembrane protein 181 (TMEM181), transcript variant X3, mRNA XM_055391004.1

```
Query  1    CTGCTGCGCGGCGCCTGGCGGGCTCGGGACGCGCGGGCCGGGGCCGAGGGCTCTGGGCGC   60
            ||||||||| ||||||||||||||||||||||||||||||||||||||||||||||||||
Sbjct  113  CTGCTGCGTGGCGCCTGGCGGGCTCGGGACGCGCGGGCCGGGGCCGAGGGCTCTGGGCGC   172

Query  61   CGAGATG   67
            |||||||
Sbjct  173  CGAGATG   179
```

note: C-G to U-G change in stem2.

Symphalangus syndactylus transmembrane protein 181 (TMEM181), transcript variant X6, mRNA XM_055268351.1

```
Query  1    CTGCTGCGCGGCGCCTGGCGGGCTCGGGACGCGCGGGCCGGGGCCGAGGGCTCTGGGCGC   60
            |||||||||||||||||||||||||||||||||||| |||||||||||||||||||||||
Sbjct  89   CTGCTGCGCGGCGCCTGGCGGGCTCGGGACGCGCGGGACGGGGCCGAGGGCTCTGGGCGC   148

Query  61   CGAGATG   67
            |||||||
Sbjct  149  CGAGATG   155
```

note: three identical sequences. C-G to A-G mismatch in extra stem.

Macaca thibetana thibetana transmembrane protein 181 (TMEM181), transcript variant X4, mRNA XM_050786733.1

```
Query  1    CTGCTGCGCGGCGCCTGGCGGGCTCGGGACGCGCGGGCCGGGGCCGAGGGCTCTGGGCGC   60
```

```
                |||||||||||||||  |||  ||||||||||||||||||||||||||||  ||||  |||||
Sbjct   75      CTGCTGCGCGGCGCCCCGGCAGGCTCGGGACGCGCGGGCCGGGGCCGAGAGCTCGGGGCGT   134

Query   61      CGAGATG   67
                |||||||
Sbjct   135     CGAGATG   141
```

Note: G-**U** to G-**C** in stem1, and G-**C** to G-**U** in stem2.

Macaca fascicularis transmembrane protein 181 (TMEM181), transcript variant X9, mRNA
XM_045391319.1

```
Query   1       CTGCTGCGCGGCGCCTGGCGGGCTCGGGACGCGCGGGCCGGGGCCGAGGGCTCTGGGCGC   60
                |||||||||||||||  |||  ||||||||||||||||||||||||||||  ||||  |||||
Sbjct   73      CTGCTGCGCGGCGCCCGGCAGGCTCGGGACGCGCGGGCCGGGGCCGAGAGCTCAGGGCGT   132

Query   61      CGAGATG   67
                |||||||
Sbjct   133     CGAGATG   139
```

Note: four identical sequences. In stem1, **U**-G TO **C**-G and **G**-U TO **A**-U; In stem2, **C**-G TO **U**-G.

Trachypithecus francoisi transmembrane protein 181 (TMEM181), transcript variant X3, mRNA
XM_033234930.1

```
Query   1       CTGCTGCGCGGCGCCTGGCGGGCTCGGGACGCGCGGGCCGGGGCCGAGGGCTCTGGGCGC   60
                |||||||||||||||  |||  ||||||||||||||||||||||||||||  |  ||  |||||
Sbjct   87      CTGCTGCGCGGCGCCCGGCAGGCTCGGGACGCGCGGGCCGGGGCCGAGAGTTCGGGGCGT   146

Query   61      CGAGATG   67
                |||||||
Sbjct   147     CGAGATG   153
```

Note: two identical sequences. in stem1, **U**-G TO **C**-G and **G**-U TO **A**-U; In stem2, **C**-G TO **U**-G.

5) NM_001009552, PPP2CB mRNA

BLASTN to 11 homologous sequences, 4 human sequences are 100% identical. The other 7 sequences all have the potentials. The last two sequences are not from primates.

Sequences that are not 100% identical are examined in the following manner:

Pongo abelii protein phosphatase 2 catalytic subunit beta (PPP2CB), transcript variant X1, mRNA
Sequence ID: XM_002818971.5

```
Query   1     GGCGGGGCGCGGGGCCCGCGGCCGCCCTACCCGGCTCAGTCCTCCCCCTGTG   52
              ||||||||||||||||||||||||||||| |||||||||||||||||||||||
Sbjct   223   GGCGGGGCGCGGGGCCCGCGGCCGCCCTCCCCGGCTCAGTCCTCCCCCTGTG   274
```

note: there are two identical sequences.

Pan troglodytes protein phosphatase 2 catalytic subunit beta (PPP2CB), mRNA
Sequence ID: XM_519697.8

```
Query  1    GGCGGGGCGCGGGGCCCGCGGCCGCCCTACCCGGCTCAGTCCTCCCCCTGTG  52
            |||||||||||| |||||||||||||||||||||||||||||| |||  |||
Sbjct  244  GGCGGGGCGCGGAGCCCGCGGCCGCCCTACCCGGCTCAGTCCTTCCCTCGTG  295
```

Note: three different nucleotides in stem2, **G**-C TO **A**-C mismatch,**C**-G to **U**-G, **U**-G to **C**-G, the stem still has 6 basepairs (4 G-C and 2 G-U) and 1 mismatch

Aotus nancymaae protein phosphatase 2 catalytic subunit beta (PPP2CB), mRNA
Sequence ID: XM_021667141.1

```
Query  1    GGCGGGGCGCGGGGCCCGCGGCCGCCCTACCCGGCTCAGTCCTCCCCCTGTG  52
            |||||||||||||||||||||||||||| |||||||| ||| |||||    |
Sbjct  39   GGCGGGGCGCGGGGCCCGCGGCCGCCCTCCCCGGCTCCGTCTTCCCCAGTGG  90
```

Note: there are 4 different nucleotides at the 3'-end , however, a strong stem2 can still form with 6 G-C basepires, 1 G-U basepair, and an A-bulge.

Saimiri boliviensis boliviensis protein phosphatase 2 catalytic subunit beta (PPP2CB), mRNA
Sequence ID: XM_039463366.1

```
Query  1    GGCGGGGCGCGGGGCCCGCGGCCGCCCTACCCGGCTCAGTCCTCCCCCTGTG  52
            |||||||||||||||||||||||||||| |||||||| ||| || ||| |||
Sbjct  210  GGCGGGGCGCGGGGCCCGCGGCCGCCCTCCCCGGCTCCGTCTTCGCCCAGTG  261
```

Note: G-**U** to G-**A** mismatch in stem2.

Equus caballus protein phosphatase 2 catalytic subunit beta (PPP2CB), mRNA
Sequence ID: XM_001493873.6

```
Query  1    GGCGGGGCGCGGGGCCCGCGGCCGCCCTACCCGGCTCAGTCCTCCCCCTGTG  52
            |||||||||||||||||||||||||||| ||||||||  | ||| |||  ||
Sbjct  263  GGCGGGGCGCGGGGCCCGCGGCCGCCCTGCCCGGCTCCTTGCTCTCCCGCTG  314
```

Note: two identical sequences. A strong stem2 can still form with one U bulge.

6) NM_007246, KLHL2 mRNA

237 hits against reference RNA seq database. 21 sequences are 100% identical.

Sequences that are not 100% identical are examined in the following manner:

Nomascus leucogenys kelch like family member 2 (KLHL2), transcript variant X2, mRNA
Sequence ID: XM_030816295.1

```
Query  1    GCGGGCAGTGCCGGCGTCCGCGGCTGGAATGGTGCTGGCTGTGTTGGTCGGTGCC  55
            ||||||||||||||||||||||||||||||||||||||| |||||||||||||||
Sbjct  96   GCGGGCAGTGCCGGCGTCCGCGGCTGGAATGGTGCTGGCGGTGTTGGTCGGTGCC  150
```

Note: 10 identical sequences.

Aotus nancymaae kelch like family member 2 (KLHL2), transcript variant X4, mRNA
Sequence ID: XM_012474566.2

```
Query  1    GCGGGCAGTGCCGGCGTCCGCGGCTGGAATGGTGCTGGCTGTGTTGGTCGGTGCC  55
            ||||||||||||||||||||||||||||||||||||||| | | |||||||||||
Sbjct  196  GCGGGCAGTGCCGGCGTCCGCGGCTGGAATGGTGCTGGCGGCGGTGGTCGGTGCC  250
```

Note: 3 identical sequences.

Phyllostomus hastatus kelch like family member 2 (KLHL2), transcript variant X2, mRNA
Sequence ID: XM_045831725.1

```
Query  1    GCGGGCAGTGCCGGCGTCCGCGGCTGGAATGGTGCTGGCTGTGTTGGTCGGTGCC  55
            | ||||||||||||||||||||||||||||||||||||| | | |||||||||||
Sbjct  177  GTGGGCAGTGCCGGCGTCCGCGGCTGGAATGGTGCTGGCAGCGGTGGTCGGTGCC  231
```

Note: 2 identical sequences, TWO other sequences **A** to **G**

Mirounga leonina kelch like family member 2 (KLHL2), transcript variant X3, mRNA
Sequence ID: XM_035022767.1

```
Query  1    GCGGGCAGTGCCGGCGTCCGCGGCTGGAATGGTGCTGGCTGTGTTGGTCGGTGCC  55
            ||||||||||||||||||||||||||||||||||||||| | |  ||||||||||
Sbjct  115  GCGGGCAGTGCCGGCGTCCGCGGCTGGAATGGTGCTGGCGGCGGCGGTCGGTGCC  169
```

Note: 114 identical sequences.

Mustela erminea kelch like family member 2 (KLHL2), transcript variant X1, mRNA
Sequence ID: XM_032332603.1

```
Query  1    GCGGGCAGTGCCGGCGTCCGCGGCTGGAATGGTGCTGGCTGTGTTGGTCGGTGCC  55
            ||| ||||||||||||||||||||||||||||||||||| | |  ||||||||||
Sbjct  45   GCGAGCAGTGCCGGCGTCCGCGGCTGGAATGGTGCTGGCGGCGGCGGTCGGTGCC  99
```

Note: 4 identical sequences. 1 **A**-C mismatch in stem 1

Ochotona princeps kelch like family member 2 (KLHL2), transcript variant X2, mRNA
Sequence ID: XM_058657428.1

```
Query  1    GCGGGCAGTGCCGGCGTCCGCGGCTGGAATGGTGCTGGCTGTGTTGGTCGGTGCC  55
            |||||| |||||||||||||||||||||||||||||||| | |  ||||||||||
Sbjct  139  GCGGGCTGTGCCGGCGTCCGCGGCTGGAATGGTGCTGGCGGCGGCGGTCGGTGCC  193
```

Note: 4 identical sequences

Marmota monax kelch like family member 2 (Klhl2), transcript variant X2, mRNA
Sequence ID: XM_046447781.2

```
Query  1    GCGGGCAGTGCCGGCGTCCGCGGCTGGAATGGTGCTGGCTGTGTTGGTCGGTGCC  55
            ||||||||||||||||||||||||||||||||||||||| | |   |||||||||
```

```
Sbjct  78    GCGGGCAGTGCCGGCGTCCGCGGCTGGAATGGTGCTGGCGGCGGCTGTCGGTGCC  132
```

Note: 2 identical sequences.

Callithrix jacchus kelch like family member 2 (KLHL2), transcript variant X3, misc_RNA
Sequence ID: XR_004740540.2

```
Query  1     GCGGGCAGTGCCGGCGTCCGCGGCTGGAATGGTGCTGGCTGTGTTGGTCGGTGCC  55
             |||||||||||||||||||||||||||||||||||||   |  |   ||||||||||
Sbjct  191   GCGGGCAGTGCCGGCGTCCGCGGCTGGAATGGTGCTGGTGGCGGCGGTCGGTGCC  245
```

Note: 3 identical sequences

Propithecus coquereli kelch-like family member 2 (KLHL2), mRNA
Sequence ID: XM_012656998.1

```
Query  1     GCGGGCAGTGCCGGCGTCCGCGGCTGGAATGGTGCTGGCTGTGTTGGTCGGTGCC  55
             |||||| ||||||||||||||||||||||||||||||||| |  |   ||||||||||
Sbjct  159   GCGGGCGGTGCCGGCGTCCGCGGCTGGAATGGTGCTGGCGGCGGCGGTCGGTGCC  213
```

Note: 3 identical sequences

Oryctolagus cuniculus kelch like family member 2 (KLHL2), transcript variant X1, mRNA
Sequence ID: XM_008273508.3

```
Query  1     GCGGGCAGTGCCGGCGTCCGCGGCTGGAATGGTGCTGGCTGTGTTGGTCGGTGCC  55
             |||||| ||||||||||||||||||||||||||||||||| |  |   ||||||||||
Sbjct  83    GCGGGCCGTGCCGGCGTCCGCGGCTGGAATGGTGCTGGCGGCGGCGGTCGGTGCC  137
```

Phodopus roborovskii kelch like family member 2 (Klhl2), mRNA
Sequence ID: XM_051181951.1

```
Query  1     GCGGGCAGTGCCGGCGTCCGCGGCTGGAATGGTGCTGGCTGTGTTGGTCGGTGCC  55
             ||||  |||||||||||||||||||||||||||||||||| |  |   ||||||||||
Sbjct  42    GCGGACAGTGCCGGCGTCCGCGGCTGGAATGGTGCTGGCGGCGGCGGTCGGTGCC  96
```

Note: 2 identical sequences. **G**-U to **A**-U in stem1.

Lutra lutra kelch like family member 2 (KLHL2), transcript variant X1, mRNA
Sequence ID: XM_047717737.1

```
Query  1     GCGGGCAGTGCCGGCGTCCGCGGCTGGAATGGTGCTGGCTGTGTTGGTCGGTGCC  55
             |||  ||||||||||||||||||||||||||||||||||| |  |   ||||||||||
Sbjct  55    GCGTGCAGTGCCGGCGTCCGCGGCTGGAATGGTGCTGGCGGCGGCGGTCGGTGCC  109
```

Note: 1 **U**-C mismatch in stem 1

Pipistrellus kuhlii kelch like family member 2 (KLHL2), transcript variant X3, mRNA
Sequence ID: XM_036423227.2

```
Query  1     GCGGGCAGTGCCGGCGTCCGCGGCTGGAATGGTGCTGGCTGTGTTGGTCGGTGCC  55
             ||||||||||||||||||||||||||||||||||||||||| |  |   || |||||||
Sbjct  266   GCGGGCAGTGCCGGCGTCCGCGGCTGGAATGGTGCTGGCGGCGGCGGGCGGTGCC  320
```

Note: 2 identical sequences. 1 G-G mismatch in stem 2

Rhinolophus ferrumequinum kelch like family member 2 (KLHL2), transcript variant X1, mRNA
Sequence ID: XM_033134531.1

```
Query  1    GCGGGCAGTGCCGGCGTCCGCGGCTGGAATGGTGCTGGCTGTGTTGGTCGGTGCC  55
            |||||||||||||||||||||||||||||||||||||||  | |   || |||  ||
Sbjct  158  GCGGGCAGTGCCGGCGTCCGCGGCTGGAATGGTGCTGGCGGCGGCGGGCGGCGCT  212
```

Note: 3 identical sequences. 1 G-G mismatch in stem 2.

PREDICTED: Sturnira hondurensis kelch like family member 2 (KLHL2), transcript variant X1, mRNA
Sequence ID: XM_037058334.1

```
Query  1    GCGGGCAGTGCCGGCGTCCGCGGCTGGAATGGTGCTGGCTGTGTTGGTCGGTGCC  55
            | |||||||||||||||||||||||||||||||||||||  | |   |||||||| |
Sbjct  157  GTGGGCAGTGCCGGCGTCCGCGGCTGGAATGGTGCTGGCGGCGGCGGTCGGTGAC  211
```

Note: 2 identical sequences. C-G to U-G in stem1.

Phyllostomus discolor kelch like family member 2 (KLHL2), transcript variant X1, mRNA
Sequence ID: XM_028519829.2

```
Query  1    GCGGGCAGTGCCGGCGTCCGCGGCTGGAATGGTGCTGGCTGTGTTGGTCGGTGCC  55
            | ||||| |||||||||||||||||||||||||||||||  | | ||||| |||||
Sbjct  152  GTGGGCATTGCCGGCGTCCGCGGCTGGAATGGTGCTGGCAGCGGTGGTCCGTGCC  206
```

Note: C-G to U-G in stem1. 1 C-C mismatch in stem 2

Microcebus murinus kelch like family member 2 (KLHL2), transcript variant X2, mRNA
Sequence ID: XM_012770581.2

```
Query  1    GCGGGCAGTGCCGGCGTCCGCGGCTGGAATGGTGCTGGCTGTGTTGGTCGGTGCC  55
            |||||| ||||||||||||||||||||||||||||||||  | |   | ||||||||
Sbjct  151  GCGGGCGGTGCCGGCGTCCGCGGCTGGAATGGTGCTGGCGGCGCCGCTCGGTGCC  205
```

Nyctereutes procyonoides kelch like family member 2 (KLHL2), transcript variant X2, mRNA
Sequence ID: XM_055336395.1

```
Query  1    GCGGGCAGTGCCGGCGTCCGCGGCTGGAATGGTGCTGGCTGTGTTGGTCGGTGCC  55
            |||||| | |||||||||||||||||||||||||||||  | |   ||||||||||
Sbjct  120  GCGGGCGGAGCCGGCGTCCGCGGCTGGAATGGTGCTGGCGGCGGCGGTCGGTGCC  174
```

Note: 6 identical sequences.

Nycticebus coucang kelch like family member 2 (KLHL2), mRNA
Sequence ID: XM_053593194.1

```
Query  1    GCGGGCAGTGCCGGCGTCCGCGGCTGGAATGGTGCTGGCTGTGTTGGTCGGTGCC  55
            |||||| |||||||||||||||||||||||||||||||  | |   ||||| ||||
Sbjct  227  GCGGGCGGTGCCGGCGTCCGCGGCTGGAATGGTGCTGGCGGCGGCGGTCGCTGCC  281
```

Note: C-C mismatch in stem 2.

Lemur catta kelch like family member 2 (KLHL2), transcript variant X1, mRNA

Sequence ID: XM_045552545.1

```
Query  1    GCGGGCAGTGCCGGCGTCCGCGGCTGGAATGGTGCTGGCTGTGTTGGTCGGTGCC  55
            |||||| | |||||||||||||||||||||||||||||| | |  ||||||||||
Sbjct  208  GCGGGCGGGGCCGGCGTCCGCGGCTGGAATGGTGCTGGCGGCGGCGGTCGGTGCC  262
```

Choloepus didactylus kelch like family member 2 (KLHL2), transcript variant X1, mRNA
Sequence ID: XM_037830960.1

```
Query  1    GCGGGCAGTGCCGGCGTCCGCGGCTGGAATGGTGCTGGCTGTGTTGGTCGGTGCC  55
            |||||||| |||||||||||||||||||||||||||||   | |  ||||||||||
Sbjct  167  GCGGGCAGGGCCGGCGTCCGCGGCTGGAATGGTGCTGGTGGCGGCGGTCGGTGCC  221
```

Talpa occidentalis kelch like family member 2 (KLHL2), transcript variant X1, mRNA
Sequence ID: XM_037526406.1

```
Query  1    GCGGGCAGTGCCGGCGTCCGCGGCTGGAATGGTGCTGGCTGTGTTGGTCGGTGCC  55
            |||||| ||||||||||||||||||||||||||||||||| | |  || |||||||
Sbjct  219  GCGGGCGGTGCCGGCGTCCGCGGCTGGAATGGTGCTGGCGGCGGCGGGCGGTGCC  273
```

Cricetulus griseus kelch like family member 2 (Klhl2), transcript variant X6, mRNA
Sequence ID: XM_027394060.2

```
Query  1    GCGGGCAGTGCCGGCGTCCGCGGCTGGAATGGTGCTGGCTGTGTTGGTCGGTGCC  55
            |||| |||||||||||||||||||||||||||||||||| | |  || |||||||
Sbjct  219  GCGGACAGTGCCGGCGTCCGCGGCTGGAATGGTGCTGGCGGCGGCGGACGGTGCC  273
```

Note: 3 identical sequences. **G**-U to **A**-U in stem1. 1 A-G mismatch in stem 2.

Microtus ochrogaster kelch like family member 2 (Klhl2), mRNA
Sequence ID: XM_005367748.3

```
Query  1   GCGGGCAGTGCCGGCGTCCGCGGCTGGAATGGTGCTGGCTGTGTTGGTCGGTGCC  55
           |||| | |||||||||||||||||||||||||||||||| | |  ||||| ||||
Sbjct  34  GCGGACCGTGCCGGCGTCCGCGGCTGGAATGGTGCTGGCGGCGGCGGTCGCTGCC  88
```

Note: 5 identical sequences. **G**-U to **A**-U in stem1. 1 **C**-C mismatch in stem 2.

Mesocricetus auratus kelch like family member 2 (Klhl2), transcript variant X2, mRNA
Sequence ID: XM_005075185.4

```
Query  1   GCGGGCAGTGCCGGCGTCCGCGGCTGGAATGGTGCTGGCTGTGTTGGTCGGTGCC  55
           |||| | | |||||||||||||||||||||||||||||| | |  ||||||||||
Sbjct  42  GCGGACGGGGCCGGCGTCCGCGGCTGGAATGGTGCTGGCGGCGGCGGTCGGTGCC  96
```

Note: 2 identical sequences. **G**-U to **A**-U in stem1. 1 **G**-G mismatch in stem 2.

Camelus ferus kelch like family member 2 (KLHL2), transcript variant X1, mRNA
Sequence ID: XM_032465504.1

```
Query  1    GCGGGCAGTGCCGGCGTCCGCGGCTGGAATGGTGCTGGCTGTGTTGGTCGGTGCC  55
            |||||| | |||||||||||||||||||||||||||||| | | ||| ||    |
SBJCT  70   GCGGGCGGGGCCGCGTCCGCGGCTGGAATGGTGCTGGCGGCGGTGGGCGCCCGC  124
```

Note: 1 **C**G bulge in stem 2.

Meriones unguiculatus kelch like family member 2 (Klhl2), transcript variant X2, mRNA
Sequence ID: XM_021637848.1

```
Query  1     GCGGGCAGTGCCGGCGTCCGCGGCTGGAATGGTGCTGGCTGTGTTGGTCGGTGCC  55
             |||| | ||||||||||||||||||||||||||||||||  |   || ||  ||||
SBJCT  183   GCGGACGGTGCCGGCGTCCGCGGCTGGAATGGTGCTGGCGGCGGCGGCCGCTGCC  137
```

Note: **G**-U to **A**-U in stem1. 1 **C**-**C** mismatch in stem 2.

Suncus etruscus kelch like family member 2 (KLHL2), transcript variant X1, mRNA
Sequence ID: XM_049772349.1

```
Query  1    GCGGGCAGTGCCGGCGTCCGCGGCTGGAATGGTGCTGGCTGTGTTGGTCGGTGCC  55
            |||||| | ||||||||||||||||||||||||||||||  |   ||     ||
SBJCT  42   GCGGGCCGAGCCGGCGTCCGCGGCTGGAATGGTGCTGGCGGCGGCGGGGCTTGGT  97
```

Note: 1 bulge in stem 2.

Peromyscus californicus insignis kelch like family member 2 (Klhl2), transcript variant X2, mRNA
Sequence ID: XM_052752362.1

```
Query  1    GCGGGCAGTGCCGGC---GTCCGCGGCTGGAATGGTGCTGGCTGTGTTGGTCGGTGCC  55
            |||| ||||||||||   ||||||||||||||||||||||||  |   ||||||||||
Sbjct  34   GCGGACAGTGCCGGCGGCGTCCGCGGCTGGAATGGTGCTGGCGGCGGCGGTCGGTGCC  91
```

Note: 6 identical sequences. **G**-U to **A**-U in stem1. Longer stem2 with 1 mismatch.

Rhinolophus sinicus kelch like family member 2 (KLHL2), transcript variant X3, mRNA
Sequence ID: XM_019744636.1

```
QUERY  12   GCGGGCAGTGCCGGCGTCCGCGGCTGGAATGGTGCTGGCTGTGTTGGTCGGTGC  54
            ||      | ||||||||||||||||||||||||||||||  |   |||||||||
SBJCT  12   GCCCCGCGGGNCGGCGTCCGCGGCTGGAATGGTGCTGGCGGCGGCGGTCGGTGC  54
```

Note: 3 identical sequences. Many different positions, but still can form three stemmed pseudoknot.

7) NM_001348255, SMIM10L2B mRNA.

```
Output file for the search:

NM_001348255 has (2817) nucleotides

Energy 1: -32.090000: Start=433 S1=5 S2=6 L1=1 L2=21 L3=0
End=476

                        TGGACTCTAGACCTACGCCGC        G

                        CCCCG     GGCCCG

TCGCGGCGGGGGGCGGAGGTGGGGC     CCGGGCAC
```

```
Energy 2: -29.530000: Start=58 S1=6 S2=7 L1=2 L2=15 L3=0 End=100

                    CATGGCGGCGTCGGC       CG

                    CCGGGC     CCGGGGC

GCGGGTCGATCGGTCGGCGGGGCCTG     GGCTCTGTC


Energy 3: -28.545000: Start=5 S1=6 S2=7 L1=1 L2=27 L3=0 End=58

                    GGCTCGAGCTCTTGCGGGTCGATCGGT       T

                    AGTCCT     GCCGCCC

                AGTGTTGGGG     CGGCGGGGC

Found #1 stem loop within loop2: Start=4, S3=6 AL=8 L2_tail=7
L2-range(0-19)
(GGCTCG)AGCTCTTG(CGGGTC)GATCGGT
```

#2 & #3 ranked pseudoknots are tandem pseudoknots. #3 ranked pseudoknot is the detected three-stemmed pseudoknot. #2 ranked pseudoknot can also harbor an extra stem within its loop2, it is therefore also a three-stemmed pseudoknot, it is not detected as that because the search setting only detects three-stemmed pseudoknots in which there is no gap between the stems.

10 BLASTN hits, all primates, 6 sequences are 100% identical, the following 4 sequences have up to 4 nt difference.
Cyan: PK1 stem1
Green: PK1 stem2
Grey: PK1 extra stem
Magenta: PK2 stem1
Yellow: PK2 stem2
Grey: PK2 extra stem
Different positions are indicated by letters in red and bold.

Pan troglodytes small integral membrane protein 10 like 2B (SMIM10L2B), mRNA
XM_024353068.2

```
QUERY  1     TTGGGGTCCCGCCGTCCTGAGGCTCGAGCTCTTGCGGGTCGATCGGTCGGCGGGGCCTGC  60
             ||||||||||||||||||||||||||||||||||||||||||||||| ||||||||||||
SBJCT  33    TTGGGGTCCCGCCGTCCTGAGGCTCGAGCTCTTGCGGGTCGATCGGCCGGCGGGGCCTGC  92

QUERY  61    GCGGGGCCCGGGCCCATGGCGGCGTCGGCGGCTCTG  96
             ||||||||||||||||||||||||||||||||||||
SBJCT  93    GCGGGGCCCGGGCCCATGGCGGCGTCGGCGGCTCTG  128
```

Symphalangus syndactylus small integral membrane protein 10-like protein 2A (LOC129475086), mRNA XM_055266964.1

```
QUERY  1    TTGGGGTCCCGCCGTCCTGAGGCTCGAGCTCTTGCGGGTCGATCGGTCGGCGGGGCCTGC  60
            ||||||||||||||||||||||||||||||| ||||||||| ||||||| ||||||||||
SBJCT  37   TTGGGGTCCCGCCGTCCTGAGGCTCGAGCTCGTGCGGGTCGGTCGGTCGTCGGGGCCTGC  96

QUERY  61   GCGGGGCCCGGGCCCATGGCGGCGTCGGCGGCTCTG  96
            ||||||||||||||||||||||||||||||||||||
SBJCT  97   GCGGGGCCCGGGCCCATGGCGGCGTCGGCGGCTCTG  132
```

Note: one C-T mismatch in stem2 of PK1 (upstream pseudoknot).

Nomascus leucogenys small integral membrane protein 10-like protein 2A (LOC100606152), mRNA XM_030807428.1

```
QUERY  1    TTGGGGTCCCGCCGTCCTGAGGCTCGAGCTCTTGCGGGTCGATCGGTCGGCGGGGCCTGC  60
            ||||||||||||||||||||||||||||||| ||||||||| ||||||| ||||||||||
SBJCT  38   TTGGGGTCCCGCCGTCCTGAGGCTCGAGCTCGTGCGGGTCGGTCGGTCGTCGGGGCCTGC  97

QUERY  61   GCGGGGCCCGGGCCCATGGCGGCGTCGGCGGCTCTG  96
            |||||||||||||||||||| |||||||||||||||
SBJCT  98   GCGGGGCCCGGGCCCATGGCTGCGTCGGCGGCTCTG  133
```

Note: one C-T mismatch in stem2 of PK1

Hylobates moloch small integral membrane protein 10 like 2B (SMIM10L2B), mRNA XM_032757670.2

```
QUERY  1    TTGGGGTCCCGCCGTCCTGAGGCTCGAGCTCTTGCGGGTCGATCGGTCGGCGGGGCCTGC  60
            ||||||||||||||||||||||||||||||| ||||||| | ||||||| ||||||||||
SBJCT  39   TTGGGGTCCCGCCGTCCTGAGGCTCGAGCTCGTGCGGGTTGGTCGGTCGTCGGGGCCTGC  98

QUERY  61   GCGGGGCCCGGGCCCATGGCGGCGTCGGCGGCTCTG  96
            ||||||||||||||||||||||||||||||||||||
SBJCT  99   GCGGGGCCCGGGCCCATGGCGGCGTCGGCGGCTCTG  134
```

Note: one C-T mismatch in stem2 of PK1, G-C TO G-U in stem3 of PK1.

8) NM_001282921, MAB21L4 mRNA

12 homologous sequences, all in primates, 7 sequences are 100% identical,

Sequences that are not 100% identical are examined in the following manner:

Macaca thibetana thibetana mab-21 like 4 (MAB21L4), transcript variant X2, mRNA

Sequence ID: XM_050752028.1

```
Query  1    TGACAGTTGTGCCGAGGACTGTCACCATTGGGCAGGCATGGTGGCCAGCTCCTCACTTGG  60
            |||||||||||||||||||| ||||||| |||||||||||||||||||||||||||||||
Sbjct  230  TGACAGTTGTGCCGAGGACGGTCACCAGTGGGCAGGCATGGTGGCCAGCTCCTCACTTGG  289

Query  61   CTGAGGTCCAGGGATG  76
            ||||||||||||||||
Sbjct  290  CTGAGGTCCAGGGATG  305
```

Note: **U**-A to **G**-A mismatch in stem 1; **U**-G to **G**-G mistmatch in stem3.

Macaca mulatta mab-21 like 4 (MAB21L4), transcript variant X3, mRNA
Sequence ID: XM_015111526.2

```
Query  1    TGACAGTTGTGCCGAGGACTGTCACCATTGGGCAGGCATGGTGGCCAGCTCCTCACTTGG  60
            |||||||||||||||||||| ||||||| ||||||||||||||||||||||||||||| ||
Sbjct  310  TGACAGTTGTGCCGAGGACGGTCACCAGTGGGCAGGCATGGTGGCCAGCTCCTCACTCGG  369

Query  61   CTGAGGTCCAGGGATG  76
            ||||||||||||||||
Sbjct  370  CTGAGGTCCAGGGATG  385
```

Note: three identical sequences. **U**-A to **G**-A mismatch in stem 1; **U**-G to **G**-G mistmatch in Stem3; **U**-G to **C**-G in stem2.

Pongo pygmaeus mab-21 like 4 (MAB21L4), transcript variant X2, mRNA
Sequence ID: XM_054478833.1

```
Query  1    TGACAGTTGTGCCGAGGACTGTCACCATTGGGCAGGCATGGTGGCCAGCTCCTCACTTGG  60
            ||||||||||||| | |||||||||||||| |||||||||||||||||||||||||||| ||
Sbjct  316  TGACAGTTGTGCTGTGGACTGTCACCATGGGGCAGGCATGGTGGCCAGCTCCTCACTCGG  375

Query  61   CTGAGGTCCAGGGATG  76
            ||||||||||||||||
Sbjct  376  CTGAGGTCCAGGGATG  391
```

Note: U-**A** to **U**-U mistmatch, **U**-G to **C**-G, **C**-G to **U**-G in stem2; **U**-G to **G**-G mistmatch in stem3

9) NM_022733, SMAP2 mRNA

121 homologous sequences. 19 sequences are 100% identical.

Sequences that are not 100% identical are examined in the following manner:

Pteropus vampyrus small ArfGAP2 (SMAP2), mRNA
Sequence ID: XM_011377972.2

```
Query  1    GGAGGAGAGGGCTCT-CCCCGCTCAGGAGGTGCCCCTGGGCGGGGGACCGGGAGTCCTC  58
            ||||||||||||||| |||||||||||||||||||||||||||||||||||||||||||
Sbjct  293  GGAGGAGAGGGCTCTCCCCCGCTCAGGAGGTGCCCCTGGGCGGGGGACCGGGAGTCCTC  351
```

Note: there are 15 identical sequences. Stem3 has one more basepair.

Gorilla gorilla gorilla small ArfGAP2 (SMAP2), transcript variant X3, mRNA
Sequence ID: XM_004025536.3

```
Query  1    GGAGGAGAGGGCTCTCCCCGCTCAGGAGGTGCCCCTGGGCGGGGGACCGGGAGTCCTC  58
            ||||||||||||||| ||||||||||||||||||||||||||||||||||||||||||
Sbjct  413  GGAGGAGAGGGCTCTTCCCGCTCAGGAGGTGCCCCTGGGCGGGGGACCGGGAGTCCTC  470
```

Note: There are three identical sequences. **C**-G to **U**-G in stem1.

Trachypithecus francoisi small ArfGAP2 (SMAP2), transcript variant X1, mRNA
Sequence ID: XM_033226955.1

```
Query  1    GGAGGAGAGGGCTCTCCCCGCTCAGGAGGTGCCCCTGGGCGGGGGACCGGGAGTCCTC  58
             ||||||||||||||||||||||||||  |||||||||||||||||||||||||||||
Sbjct  300  AGAGGAGAGGGCTCTCCCCGCTCAGGATTTGCCCCTGGGCGGGGGACCGGGAGTCCTC  357
```

Note: there are 14 sequences.

Propithecus coquereli small ArfGAP2 (SMAP2), mRNA
Sequence ID: XM_012646758.1

```
Query  1    GGAGGAGAGGGCTCTCCCC-GCTCAGGAGGTGCCCCTGGGCGGGGGACCGGGAGTCCTC  58
            ||||||||||||||||||| ||||||||||||||||||||||||||||| || ||||||
Sbjct  312  GGAGGAGAGGGCTCTCCCCAGCTCAGGAGGTGCCCCTGGGCGGGGGACCCGGGGTCCTC  370
```

Note: one bulge in stem3.

Carlito syrichta small ArfGAP2 (SMAP2), transcript variant X1, mRNA
Sequence ID: XM_008065502.2

```
Query  1    GGAGGAGAGGGCTCT-CCCCGCTCAGGAGGTGCCCCTGGGCGGGGGACCGGGAGTC  55
            ||||||||||||||| ||||||||||| ||||||||||||||||||||||||||||
Sbjct  315  GGAGGAGAGGGCTCTCCCCCGCTCAGGTGGTGCCCCTGGGCGGGGGACCGGGAGTC  370
```

Note: stem3 has one more basepair.

Sus scrofa small ArfGAP2 (SMAP2), transcript variant X2, mRNA
Sequence ID: XM_013977380.2

```
Query  1    GGAGGAGAGGGCTCT-CCCCGCTCAGGAGGTGCCCCTGGGCGGGGGACCGGGAGTCCTC  58
               |||||||||||| ||||||||||||||||||||||||| |||||||||||||||||
Sbjct  310  AGGGGAGAGGGCTCTCCCCCGCTCAGGAGGTGCCCCTGGGC-GGGGACCGGGAGTCCTC  367
```

Note: there are 6 sequences. Stem1 has one less basepair. Stem3 has two more basepairs.

Balaenoptera acutorostrata small ArfGAP2 (SMAP2), mRNA
Sequence ID: XM_007164698.2

```
Query  1     GGAGGAGAGGGCTCT-CCCCGCTCAGGAGGTGCCCCTGGGCGGGGGACCGGGAGTCCTC  58
                |||||||||||| |||||||||||||||||||||||||||||||| ||||||||||
Sbjct  293  GGTGGGAGAGGGCTCTCCCCCGCTCAGGAGGTGCCCCTGGGCGGGGGACCAGGAGTCCTC  352
```

Note: there are 2 sequences. Stem1 has one bulge. Stem3 has one more basepair.

Orcinus orca small ArfGAP2 (SMAP2), transcript variant X2, mRNA
Sequence ID: XM_049698010.1

```
Query  1     GGAGGAGAGGGCTC-TCCCCGCTCAGGAGGTGCCCCTGGGCGGGGGACCGGGAGTCCTC  58
                |||||||||||| |||||||||||||||||||||||||||||||| ||||||||||
Sbjct  289 GGTGGGAGAGGGCTCTTCCCCGCTCAGGAGGTGCCCCTGGGCGGGGGACCAGGAGTCCTC  348
```

Note: there are 2 sequences. Stem3 has one more basepair.

Lemur catta small ArfGAP2 (SMAP2), transcript variant X1, mRNA
Sequence ID: XM_045548138.1

```
Query  1    GGAGGAGAGGGCTCT-CCCCGCTCAGGAGGTGCCCCTGGGCGGGGGACCGGGAGTCCTC  58
            ||||||||||||||| ||||||||||||||||||||||||||||||| || ||||||
Sbjct  314  GGAGGAGAGGGCTCTCCCCCGCTCAGGAGGTGCCCCTGGGCGGGGGACCCGGGGTCCTC  372
```

Note: Stem3 has one more basepair.

Oryctolagus cuniculus small ArfGAP2 (SMAP2), mRNA
Sequence ID: XM_002715186.4

```
Query  1    GGAGGAGAGGGCTCT-CCCCGCTCAGGAGGTGCCCCTGGGCGGGGACCGGGAGTCCTC  58
             |||||||||||||| ||||||||||||||||||||||||||||| |||||||| |||
Sbjct  301  AGAGGAGAGGGCTCTCCCCCGCTCAGGAGGTGCCCCTGGGCGGGGGCCGGGAGTTCTC  359
```

Note: Stem1 has one less basepair, Stem3 has two more basepair. G-**C** to G-**U** in stem2.

Eumetopias jubatus small ArfGAP2 (SMAP2), mRNA
Sequence ID: XM_028123232.1

```
Query  1    GGAGGAGAGGGCTCT-CCCCGCTCAGGAGGTGCCCCTGGGCGGGGGACCGGGAGTCCTC  58
            || || ||||||| | |||||||||||||||||||||||||||||||||||||||||||
Sbjct  267  GGTGGGGAGGGCTTTCCCCCGCTCAGGAGGTGCCCCTGGGCGGGGGACCGGGAGTCCTC  325
```

Note: there 6 identical sequences. U-U mismatch in stem1.

Microcebus murinus small ArfGAP2 (SMAP2), transcript variant X1, mRNA
Sequence ID: XM_012735775.2

```
Query  1    GGAGGAGAGGGCTCT-CCCCGCTCAGGAGGTGCCCCTGGGCGGGGGACCGGGAGTCCTC  58
            |||||||| |||||| ||||||||||||||||||||||||||||||||| || ||||||
Sbjct  314  GGAGGAGAAGGCTCTCCCCCGCTCAGGAGGTGCCCCTGGGCGGGGGACCCGGGGTCCTC  372
```

Note: A-c mismatch in stem2. One more basepair in stem3.

Ailuropoda melanoleuca small ArfGAP2 (SMAP2), transcript variant X2, mRNA
Sequence ID: XM_034650029.1

```
Query  1    GGAGGAGAGGGCTCT-CCCCGCTCAGGAGGTGCCCCTGGGCGGGGGACCGGGAGTCCTC  58
             |||| ||||| | | |||||||||||||||||||||||||||||||||||||||||||
Sbjct  272  CGAGGGGAGGGATTTCCCCCGCTCAGGAGGTGCCCCTGGGCGGGGGACCGGGAGTCCTC  330
```

Note: there are 6 sequences. Stem1 has one less basepair, Stem2 has one less basepair, Stem3 has two more basepairs. G-C to G-U in stem1.

Camelus ferus small ArfGAP2 (SMAP2), transcript variant X1, mRNA
Sequence ID: XM_006174504.3

```
Query  1    GGAGGAGAGGGCTCT-CCCCGCTCAGGAGGTGCCCCTGGGCGGGGGACCGGGAGTCCTC  58
            || |||||||||||| |||||||||||||||||||||||||||||  | ||||||||||
Sbjct  306  GGGGGAGAGGGCTCTCCCCCGCTCAGGAGGTGCCCCTGGGCGGGGAGCTGGGAGTCCTC  364
```

Note: there are 4 sequences. Stem3 has one more basepair. A-U to G-U in stem1.

Otolemur garnettii small ArfGAP2 (SMAP2), transcript variant X1, mRNA
Sequence ID: XM_003798975.3

```
Query  1    GGAGGAGAGGGCTCT-CCCCGCTCAGGAGGTGCCCCTGGGCGGGGGACCGGGAGTCCTC  58
            ||||||| | ||||| ||||||||||||||||||||||||||||||||| || ||||||
Sbjct  285  GGAGGAGCGCGCTCTCCCCCGCTCAGGAGGTGCCCCTGGGCGGGGGACCCGGTGTCCTC  343
```

Note: Stem3 has one more basepair. Two mismatches in stem2.

Elephantulus edwardii small ArfGAP2 (SMAP2), mRNA
Sequence ID: XM_006886507.1

```
Query  1    GGAGGAGAGGGCTCT-CCCCGCTCAGGAGGTGCCCCTGGGCGGGGGACCGGGAGTCCTC  58
             ||| | ||| |||| |||||||||||||||||||||||||||||||||||||||| ||
Sbjct  231  TGAGAAAAGGACTCTACCCCGCTCAGGAGGTGCCCCTGGGCGGGGGACCGGGAGTCTTC  288
```

Note: Stem 1 has two less basepairs, stem2 has one less basepair, Stem3 has two more basepairs.

Hippopotamus amphibius kiboko small ArfGAP2 (SMAP2), mRNA
Sequence ID: XM_057706737.1

```
Query  1    GGAGGAGAGGGCTCT-CCCCGCTCAGGAGGTGCCCCTGGGCGGGGGACCGGGAGTCCTC  58
            || || | ||||||| |||||||||||||||||||||||||||||| ||||| ||||||
Sbjct  268  GGGGGCGCGGGCTCTCCCCCGCTCAGGAGGTGCCCCTGGGCGGGGGGCCGGGGGTCCTC  326
```

Note: **A**-U to **G**-U in stem 1, **C**-U mismatch in stem2, Stem3 has one more basepair.

Elephas maximus indicus small ArfGAP2 (SMAP2), transcript variant X1, mRNA
Sequence ID: XM_049878912.1

```
Query  1    GGAGGAGAGGGCTCT-CCCCGCTCAGGAGGTGCCCCTGGGCGGGGGACCGGGAGTCCTC  58
             |||||||| ||||| |||||||||||||||||||||||||||||||| | |||| ||
Sbjct  285  TGAGGAGAGAGCTCTCCCCCGCTCAGGAGGTGCCCCTGGGCGGGGGACAGAGAGTTCTT  342
```

Note: one less basepair in stem 1, **G-C** to **A-U** covariation, and G-**C** to G-**U** in stem2, Stem3 has two more basepairs.

Meles meles small ArfGAP2 (SMAP2), transcript variant X1, mRNA
Sequence ID: XM_045998505.1

```
Query  1    GGAGGAGAGGGCT-C-TCCCCGCTCAGGAGGTGCCCCTGGGCGGGGGACCGGGAGTCCTC  58
             |||||| ||| | | |||||||||||||||||||||||||||||||||  |||||||||
Sbjct  276  AGAGGAG-GGGTTCCATCCCCGCTCAGGAGGTGCCCCTGGGCGGGGGACTAGGAGTCCTC  334
```

Echinops telfairi small ArfGAP2 (SMAP2), mRNA
Sequence ID: XM_004704934.2

```
Query  1    GGAGGAGAGGGCTCT-CCCCGCTCAGGAGGTGCCCCTGGGCGGGGGACCGGGAGTCCTC  58
             |  ||| | ||||| |||||||||||||||||||||||||||||| || ||||||||
Sbjct  251  TGCCGAGGGTGCTCTCCCCCGCTCAGGAGGTGCCCCTGGGCGGGGGGCCCGGAGTCCTT  309
```

Note: stem1 having two mismatches.

Lontra canadensis small ArfGAP2 (SMAP2), mRNA
Sequence ID: XM_032877410.1

```
Query  1     GGAGGAGAGGGCTCTC-CCC-GCTCAGGAGGTGCCCCTGGGCGGGGGACCGGGAGTCCTC  58
              |||||| ||   || |  |||  ||||  ||||||||||||||||||||||||||||||||||
Sbjct  257  AGAGGAGGGGTTTCCCGCCCGGCTCCGGAGGTGCCCCTGGGCGGGGGACCGGGAGTCCTC  316
```

Enhydra lutris kenyoni small ArfGAP2 (LOC111143109), mRNA
Sequence ID: XM_022496591.1

```
Query  1     GGAGGAGAGGGCTCTCCCCGCTCAGGAGGTGCCCCTGGGCGGGGGACCGGGAGTCCTC  58
                             |||||||||||||||||||||||||||||||||||||||||||
Sbjct 231GAGAGGAGGGGTTTCCCGCCCCGCTCAGGAGGTGCCCCTGGGCGGGGGACCGGGAGTCCTC  291
```

note: blastn only shows homology for nt 16-58. Although very different 5'-sequences, still has the potential to form three-stemmed pseudoknot.

Mustela lutreola small ArfGAP2 (SMAP2), mRNA
Sequence ID: XM_059134875.1

```
Query  1     GGAGGAGAGGGCTCTCCCCGCTCAGGAGGTGCCCCTGGGCGGGGGACCGGGAGTCCTC  58
                             |||||||||||||||||||||||||||||||||||||||||||
Sbjct  304                   CCCCGCTCAGGAGGTGCCCCTGGGCGGGGGACCGGGAGTCCTC  364

291 GGGCCGGAGAGGAGGGGTTACCCCCCTCCCG-303
```

Note: BLASTN only shows homology for nt 16-58. There seems to be a C-rich insertion sequence upstream from the BLASTN matched sequence.

Nyctereutes procyonoides small ArfGAP2 (SMAP2), mRNA
Sequence ID: XM_055341802.1

```
Query  1     GGAGGAGAGGGCTCTCCCCGCTCAGGAGGTGCCCCTGGGCGGGGGACCGGGAGTCCTC  58
                             |||||||||||||||||||||||||||||||||||||||||||
Sbjct  335                   CCCCGCTCAGGAGGTGCCCCTGGGCGGGGGACCGGGAGTCCTC  377

291   CCCGGCCCGAGGGAGGGCTCCCCACCCCCACCCCCACCCCTCAC-334
```

Note: BLASTN only shows homology for nt 16-58. There seems to be a C-rich insertion sequence upstream from the BLASTN matched sequence.

Lutra lutra small ArfGAP2 (SMAP2), mRNA
Sequence ID: XM_047724595.1

```
Query   1     GGAGGAGAGGGCTCTCCCCGCTCAGGAGGTGCCCCTGGGCGGGGGACCGGGAGTCCTC  58
                              |||||||||||||||||||||||||||||||||||||||||||
Sbjct 235 CGAGTGGAGGGGTTTCCCGCCCCGCTCAGGAGGTGCCCCTGGGCGGGGGACCGGGAGTCCTC  296
```

Note: BLASTN only shows homology for nt 16-58. Although the 5’- sequences ae very different, still has the potential for a three-stemmed pseudoknot.

Neovison vison small ArfGAP2 (SMAP2), mRNA
Sequence ID: XM_044237925.1

```
Query  1                 GGAGGAGAGGGCTCTCCCCGCTCAGGAGGTGCCCCTGGGCGGGGGACCGGGAGTCCTC  58
                                   |||||||||||||||||||||||||||||||||||||||||||
Sbjct  215   GGAGGGGTTCCCCCCCCCCCGCCGCCCCGCTCAGGAGGTGCCCCTGGGCGGGGGACCGGGAGTCCTC 282
```

Note: BLASTN only shows homology for nt 16-58. Although the 5’- sequences ae very different, still has the potential for a three-stemmed pseudoknot.

Vulpes lagopus small ArfGAP2 (SMAP2), mRNA
Sequence ID: XM_041738623.1

```
Query  1     GGAGGAGAGGGCTCTCCCCGCTCAGGAGGTGCCCCTGGGCGGGGGACCGGGAGTCCTC  58
                           |||||||||||||||||||||||||||||||||||||||||||
Sbjct  376                 CCCCGCTCAGGAGGTGCCCCTGGGCGGGGGACCGGGAGTCCTC  418

321 GCCCGAGGGAGGGCTCCCCACCCCCACCCCTCACCCCTCACCCCTCACTCCTCAC-375
```

Note: BLASTN only shows homology for nt 16-58. There seems to be a C-rich insertion sequence upstream from the BLASTN matched sequence.

Canis lupus familiaris small ArfGAP2 (SMAP2), transcript variant X1, mRNA
Sequence ID: XM_539575.7

```
Query  1     GGAGGAGAGGGCTCTCCCCGCTCAGGAGGTGCCCCTGGGCGGGGGACCGGGAGTCCTC  58
                           |||||||||||||||||||||||||||||||||||||||||||
Sbjct  320                 CCCCGCTCAGGAGGTGCCCCTGGGCGGGGGACCGGGAGTCCTC  362

271 CGAGGGAGGGCTCCCCACCCCCACCCCCACCCCCACCCTCACCCCTCAC-319
```

Note: There are 13 identical sequences. BLASTN only shows homology for nt 16-58. There seems to be a C-rich insertion sequence upstream from the BLASTN matched sequence.

Kogia breviceps small ArfGAP2 (SMAP2), mRNA
Sequence ID: XM_059044125.1

```
Query  1     GGAGGAGAGGGCTCT-CCCCGCTCAGGAGGTGCCCCTGGGCGGGGGACCCGGGAGTCCTC  58
             |   ||||||||||||| |||||||||||||||||||||||||||||||||
Sbjct  252 GGTGGGAGAGGGCTCTCCCCCGCTCAGGAGGTGCCCCTGGGCGGGGGACCAGCTCCGGGCT   312
```

Note: BLASTN only shows homology for nt 4-48. Although the 5’- & 3’- sequences ae very different, still has the potential for a three-stemmed pseudoknot.

Mustela putorius furo small ArfGAP2 (SMAP2), transcript variant X1, mRNA
Sequence ID: XM_045064284.1

```
Query   1         GGAGGAGAGGGCTCTCCCCGCTCAGGAGGTGCCCCTGGGCGGGGGACCGGGAGTCCTC  58
                                 ||||||||||||||||||||||||||||||||||||||||| |
Sbjct 291   GGAGGGGTTCCCCCCCTCCCGCCCCGCTCAGGAGGTGCCCCTGGGCGGGGGACCGGGAGTCCAC  354
```

Note: There are 2 identical sequences. BLASTN only shows homology for nt 16-58. Although the 5'- sequences ae very different, still has the potential for a three-stemmed pseudoknot.

Panthera leo small ArfGAP2 (SMAP2), transcript variant X1, mRNA
Sequence ID: XM_042949467.1

```
Query   1    GGAGGAGAGGGCTCTCCCCGCTCAGGAGGTGCCCCTGGGCGGGGGACCGGGAGTCCTC   58
                         || ||||||||||||||||||||||||||||||||| |||||||||
Sbjct 287   GGGGGAGGGCTTTTCCCCCCGCTCAGGAGGTGCCCCTGGGCGGGGGACCCGGAGTCCTC   335
```

Note: three 12 identical sequences. BLASTN only shows homology for nt 13-58. Although the 5'- sequences ae very different, still has the potential for a comparable three-stemmed pseudoknot, stem2 has one more basepairs.

10) NM_001037, SCN1B mRNA

36 BLASTN hits, all from primates. 32 sequences are 100% identical.
Sequences that are not 100% identical are examined in the following manner:

Papio anubis sodium voltage-gated channel beta subunit 1 (SCN1B), transcript variant X1, mRNA
Sequence ID: XM_003915317.3

```
Query  1     GCAGCCATGGGGAGGCTGCTGGCCTTAGTGGTCGGCGCGGCACTGGTGTCCTCA   54
             ||||| ||||||||||||||||||||||||||||||||||||||||||||||||
Sbjct  341   GCAGCTATGGGGAGGCTGCTGGCCTTAGTGGTCGGCGCGGCACTGGTGTCCTCA   394
```

Note: C-G to U-G in stem1.

Piliocolobus tephrosceles sodium voltage-gated channel beta subunit 1 (SCN1B), mRNA
Sequence ID: XM_023197000.2

```
Query  1      GCAGCCATGGGGAGGCTGCTGGCCTTAGTGGTCGGCGCGGCACTGGTGTCCTCA   54
              ||||||||||||||||||||||| ||||||||||||||||||||||||||||||
Sbjct  1209   GCAGCCATGGGGAGGCTGCTGGCTTTAGTGGTCGGCGCGGCACTGGTGTCCTCA   1262
```

Note: C-G to U-G in stem3.

Pongo abelii sodium voltage-gated channel beta subunit 1 (SCN1B), mRNA
Sequence ID: XM_002829050.4

```
Query  1     GCAGCCATGGGGAGGCTGCTGGCCTTAGTGGTCGGCGCGGCACTGGTGTCCTCA   54
             ||||||||||| |||||||||||||||||||||||||||||| |||||||||
Sbjct  347   GCAGCCATGGGAAGGCTGCTGGCCTTAGTGGTCGGCGCGGCACTCGTGTCCTCA   400
```

Note: G-C to A-C mismatch in stem2.

Pongo pygmaeus sodium voltage-gated channel beta subunit 1 (SCN1B), transcript variant X2, mRNA

Sequence ID: XM_054464719.1

```
Query  1    GCAGCCATGGGGAGGCTGCTGGCCTTAGTGGTCGGCGCGGCACTGGTGTCCTCA  54
            ||||||||||| ||||||||||||||||||||||||||||||| ||||||||||
Sbjct  349  GCAGCCATGGGAAGGCTGCTGGCCTTAGTGGTCGGCGCGGCATTGGTGTCCTCA  402
```

Note: **G**-C to **A**-C mismatch in stem2. There are two sequences.

11) NM_004332, BPHL mRNA

39 BLASTN hits, all from primates. 15 sequences are 100% identical.
Sequences that are not 100% identical are examined in the following manner:

Gorilla gorilla gorilla biphenyl hydrolase like (BPHL), transcript variant X9, mRNA
Sequence ID: XM_055390931.1

```
Query  1    TGGCTGTGCTGGGCGGCCGGGGCGTGTTGCGCCTGCGGCTGCTTCTCTCAGCGC  54
            |||||||||||||||||||||||||||||||||||||||||||| ||||||||||
Sbjct  87   TGGCTGTGCTGGGCGGCCGGGGCGTGTTGCGCCTGCGGCTGCTGCTCTCAGCGC  140
```

Note: There are 24 sequences.

12) NM_005376, MYCL mRNA

30 BLASTN hits, all from primates. 8 sequences are 100% identical.
Sequences that are not 100% identical are examined in the following manner:

Trachypithecus francoisi MYCL proto-oncogene, bHLH transcription factor (MYCL), transcript variant X2, mRNA
Sequence ID: XM_033226985.1

```
Query  1    CGCGCTCTCGGCGGCGCGCATGTGCGTGTGTGCTGGCTGCCGGG  44
            |||||||||||||||||||||||||||||||||| ||||||||||
Sbjct  130  CGCGCTCTCGGCGGCGCGCATGTGCGTGTGTGCGGGCTGCCGGG  173
```

Note: There are 17 sequences.

Nomascus leucogenys MYCL proto-oncogene, bHLH transcription factor (MYCL), transcript variant X2, mRNA
Sequence ID: XM_012511042.2

```
Query  1    CGCGCTCTCGGCGGCGCGCATGTGCGTGTGTGCTGGCTGCCGGG  44
            ||||||||||||||||||||||||||||||||| || |||||||
Sbjct  5    CGCGCTCTCGGCGGCGCGCATGTGCGTGTGTGCGGGTTGCCGGG  48
```

Note: There are 3 sequences.

Pongo abelii MYCL proto-oncogene, bHLH transcription factor (MYCL), mRNA
Sequence ID: XM_002810996.4

```
Query  1    CGCGCTCTCGGCGGCGCGCATGTGCGTGTGTGCTGGCTGCCGGG  44
            ||||||||||||||||||||||||||||||||   ||||||||||
Sbjct  12   CGCGCTCTCGGCGGCGCGCATGTGCGTGTGTGAGGGCTGCCGGG  55
```

Note: There are 2 sequences.

13) NM_012144, DNAI1 mRNA

58 BLASTN hits, all from primates. 16 sequences are 100% identical.

Sequences that are not 100% identical are examined in the following manner:

Nomascus leucogenys dynein axonemal intermediate chain 1 (DNAI1), mRNA
Sequence ID: XM_030818385.1

```
Query  1    GGGAGCGTTTTGTAGGCTCTCCAGGGGTTGAGATGATTCCTGCTTCTGCGAA  52
            ||||||||||||||||||||||||||||||||||| ||||||||||||||||
Sbjct  221  GGGAGCGTTTTGTAGGCTCTCCAGGGGTTGAGATGCTTCCTGCTTCTGCGAA  272
```

Note: There are 41 sequences.

Propithecus coquereli dynein, axonemal, intermediate chain 1 (DNAI1), transcript variant X1, mRNA
Sequence ID: XM_012656020.1

```
Query  1    GGGAGCGTTTTGTAGGCTCTCCAGGGGTTGAGATGATTCCTGCTTCTGCGAA  52
            ||| ||||||||||||||||||||||||||||||| ||||||||||||||||
Sbjct  142  GGG-GCGTTTTGTAGGCTCTCCAGGGGTTGAGATGCTTCCTGCTTCTGCGAA  192
```

14) NM_004787, SLIT2 mRNA

257 BLASTN hits, all from primates. 83 sequences are 100% identical.
Sequences that are not 100% identical are examined in the following manner:

Theropithecus gelada slit guidance ligand 2 (SLIT2), transcript variant X4, mRNA
Sequence ID: XM_025385836.1

```
Query  1     GCGGCGTTGGCTGGCAGATGCTGTCCCTGTCGCTGGGGTTAGTGCTGGC  49
             | |||||||||||||||||||||||||||||||||||||||||||||||
Sbjct  1912  GTGGCGTTGGCTGGCAGATGCTGTCCCTGTCGCTGGGGTTAGTGCTGGC  1960
```

Note: **C**-G to **U**-G change in stem1. There are 4 sequences.

Nomascus leucogenys slit guidance ligand 2 (SLIT2), mRNA
Sequence ID: XM_030801111.1

```
Query  1     GCGGCGTTGGCTGGCAGATGCTGTCCCTGTCGCTGGGGTTAGTGCTGGC  49
             ||||||||||||||||||||||||||||||||||||||||||| |||||
Sbjct  1935  GCGGCGTTGGCTGGCAGATGCTGTCCCTGTCGCTGGGGTTAGTACTGGC  1983
```

Note: C-**G** to C-**A** mismatch in stem2. There are 7 sequences.

Galeopterus variegatus slit homolog 2 protein-like (LOC103599671), transcript variant X2, mRNA
Sequence ID: XM_008583815.1

```
Query  1    GCGGCGTTGGCTGGCAGATGCTGTCCCTGTCGCTGGGGTTAGTGCTGGC  49
            |||||| ||||||||||||||||||||||||||||||||||||||||||
Sbjct  534  GCGGCGCTGGCTGGCAGATGCTGTCCCTGTCGCTGGGGTTAGTGCTGGC  582
```

Note: **U**-A to **C**-A mismatch in stem1. There are 2 sequences.

Orycteropus afer afer slit guidance ligand 2 (SLIT2), mRNA
Sequence ID: XM_042782686.1

```
Query  1   GCGGCGTTGGCTGGCAGATGCTGTCCCTGTCGCTGGGGTTAGTGCTGGC  49
           |||||||||||||||||| ||||||||||||||||||||||||||||||
Sbjct  5   GCGGCGTTGGCTGGCAGACGCTGTCCCTGTCGCTGGGGTTAGTGCTGGC  53
```

Note: G-**U** to G-**C** change in stem1.

Tursiops truncatus slit guidance ligand 2 (SLIT2), transcript variant X6, mRNA
Sequence ID: XM_033856894.1

```
Query  1     GCGGCGTTGGCTGGCAGATGCTGTCCCTGTCGCTGGGGTTAGTGCTGGC  49
             ||||||| |||||||||| ||||||||||||||||||||||||||||||
Sbjct  1538  GCGGCGTGGGCTGGCAGACGCTGTCCCTGTCGCTGGGGTTAGTGCTGGC  1586
```

Note: G-**U** to G-**C** change in stem1. Stem1 has 1 less basepair. Loop1 has one more nt. There are 45 sequences.

Camelus ferus slit guidance ligand 2 (SLIT2), transcript variant X4, mRNA
Sequence ID: XM_006184696.3

```
Query  1     GCGGCGTTGGCTGGCAGATGCTGTCCCTGTCGCTGGGGTTAGTGCTGGC  49
             ||||||| |||||||||| ||||||||||||||||||||||||||||||
Sbjct  1943  GCGGCGTCGGCTGGCAGACGCTGTCCCTGTCGCTGGGGTTAGTGCTGGC  1991
```

Note: **U**-G to **C**-G and G-**U** to G-**C** changes in stem1. Stem1 is stronger. There are 14 sequences.

Loxodonta africana slit guidance ligand 2 (SLIT2), mRNA
Sequence ID: XM_003411373.3

```
Query  1     GCGGCGTTGGCTGGCAGATGCTGTCCCTGTCGCTGGGGTTAGTGCTGGC  49
             |||||||||||| ||||| ||||||||||||||||||||||||||||||
Sbjct  1904  GCGGCGTTGGCTTGCAGACGCTGTCCCTGTCGCTGGGGTTAGTGCTGGC  1952
```

Note: G-**U** to G-**C** change in stem1. **G**-U to **U**-U mismatch in stem2. There are 3 sequences.

Callithrix jacchus slit guidance ligand 2 (SLIT2), transcript variant X2, mRNA
Sequence ID: XM_035293962.2

```
Query  1    GCGGCGTTGGCTGGCAGATGCTGTCCCTGTCGCTGGGGTTAGTGCTGGC  49
            ||||||| | |||||||||||||||||||||||||||||||||||||||
Sbjct  97   GCGGCGTCGACTGGCAGATGCTGTCCCTGTCGCTGGGGTTAGTGCTGGC  145
```

Note: **U**-G to **C**-G change in stem1. Stem2 has 1 less basepair. Loop1 has one more nt. There are 8 sequences.

Lontra canadensis slit guidance ligand 2 (SLIT2), transcript variant X4, mRNA
Sequence ID: XM_032854264.1

```
Query  1    GCGGCGTTGGCTGGCAGATGCTGTCCCTGTCGCTGGGGTTAGTGCTGGC  49
            ||||||| |||||||||| ||||||||||||||||||||||||||||| 
Sbjct  562  GCGGCGTCGGCTGGCAGACGCTGTCCCTGTCGCTGGGGTTAGTGCTGGT  610
```

Note: **U**-G to **C**-G and G-**U** to G-**C** changes in stem1. Stem1 is stronger.
G-**C** to G-**U** change in Stem2. There are 18 sequences.

Trichechus manatus latirostris slit guidance ligand 2 (LOC101343863), transcript variant X4, mRNA
Sequence ID: XM_004375152.3

```
Query  3     GCGGCGTTGGCTGGCAGATGCTGTCCCTGTCGCTGGGGTTAGTGCTGGC  49
             | ||||||||||||||||  |||||||||||||||||||||||||||||
Sbjct  1894  GTGGCGTTGGCTGGCAGACACTGTCCCTGTCGCTGGGGTTAGTGCTGGC  1940
```

Note: **C**-G to **U**-G and G-**U** to G-**C** changes, as well as C-**G** to C-**A** mismatch in stem1.

```
There are 4 sequences.
```

Otolemur garnettii slit guidance ligand 2 (SLIT2), mRNA
Sequence ID: XM_023515340.1

```
Query  1     GCGGCGTTGGCTGGCAGATGCTGTCCCTGTCGCTGGGGTTAGTGCTGGC  49
             |||||| ||||||||||| ||||||||||||||||||||||||||||| |
Sbjct  171   GCGGCGCTGGCTGGCAGACGCTGTCCCTGTCGCTGGGGTTAGTGCTGAC  217
```

Note: G-**U** to G-**C** and **U**-A to **C**-A mismatch in stem1. C-**G** to C-**A** mismatch in stem2. There are 4 sequences.

Gulo gulo luscus SLIT2 (SLIT2) gene, complete cds
Sequence ID: OM350682.1

```
Query  1    GCGGCGTTGGCTGGCAGATGCTGTCCCTGTCGCTGGGGTTAGTGCTGGC  49
             |||||| |||||||||| |||||||||||||||||||||||||||||
Sbjct  5    CCGGCGTCGGCTGGCAGACGCTGTCCCTGTCGCTGGGGTTAGTGCTGGT  53
```

Note: **U**-G to **C**-G and G-**U** to G-**C** changes in stem1. Stem1 has one less basepair.
G-**C** to G-**U** change in Stem2. There are 18 sequences.

Mirounga leonina slit guidance ligand 2 (SLIT2), transcript variant X6, misc_RNA
Sequence ID: XR_004679286.1

```
Query  1     GCGGCGTTGGCTGGCAGATGCTGTCCCTGTCGCTGGGGTTAGTGCTGGC  49
             | ||||| |||||||||| |||||||||||||||||||||||||||||
Sbjct  559   GTGGCGTCGGCTGGCAGACGCTGTCCCTGTCGCTGGGGTTAGTGCTGGT 607
```

Note: **U**-G to **C**-G, G-**U** to G-**C**, and **C**-G to **U**-G changes in stem1. G-**C** to G-**U** change in Stem2. There are 25 sequences.

Vicugna pacos slit guidance ligand 2 (SLIT2), transcript variant X4, mRNA
Sequence ID: XM_006208114.3

```
Query  1     GCGGCGTTGGCTGGCAGATGCTGTCCCTGTCGCTGGGGTTAGTGCTGGC  49
             ||||||| |||||||||| | ||||||||||||||||||||||||||||
Sbjct  597   GCGGCGTCGGCTGGCAGACGTTGTCCCTGTCGCTGGGGTTAGTGCTGGC  645
```

Note: **U**-G to **C**-G, G-**U** to G-**C**, and G-**C** to G-**U** changes in stem1. There are 5 sequences.

Pteropus vampyrus slit guidance ligand 2 (SLIT2), mRNA
Sequence ID: XM_023533952.1

```
Query  1     GCGGCGTTGGCTGGCAGATGCTGTCCCTGTCGCTGGGGTTAGTGCTGGC  49
             ||||||  |||||||||| ||||||||||||||||||||||||||||||
Sbjct  415   GCGGCGCCGGCTGGCAGACGCTGTCCCTGTCGCTGGGGTTAGTGCTGGC  463
```

Note: **U**-G to **C**-G and G-**U** to G-**C** changes, as well as **T**-A to **C**-A mismatch in stem1. There are 12 sequences.

Ailuropoda melanoleuca slit guidance ligand 2 (SLIT2), transcript variant X4, mRNA
Sequence ID: XM_011221190.3

```
Query  1    GCGGCGTTGGCTGGCAGATGCTGTCCCTGTCGCTGGGGTTAGTGCTGGC  48
            ||||||| || ||||||| |||||||||||||||||||||||||||||
Sbjct  42   GCGGCGTCGGTTGGCAGACGCTGTCCCTGTCGCTGGGGTTAGTGCTGGT  89
```

Note: **U**-G to **C**-G and G-**U** to G-**C** changes in stem1. **C**-G to **U**-G and G-**C** to G-**U** changes in stem2. There are 9 sequences.

Eumetopias jubatus slit guidance ligand 2 (SLIT2), transcript variant X4, mRNA
Sequence ID: XM_028088545.1

```
Query  1    GCGGCGTTGGCTGGCAGATGCTGTCCCTGTCGCTGGGGTTAGTGCTGGC  49
            | ||||| || ||||||| ||||||||||||||||||||||||||||
Sbjct  5    GTGGCGTGGGTTGGCAGACGCTGTCCCTGTCGCTGGGGTTAGTGCTGGT  53
```

Note: **C**-G to **U**-G, **U**-G to **G**-G mismatch, and G-**U** to G-**C** changes in stem1. G-**C** to G-**U** change in stem2. There are 16 sequences.
