# Supplementary material for: Possible involvement of three-stemmed pseudoknots in regulating translational initiation in human mRNAs
Source: PLoS One. 2024 Jul 22;19(7):e0307541. doi: 10.1371/journal.pone.0307541 (PMC11262651; doi:10.1371/journal.pone.0307541)
Supplement: S3 File — (PDF) [file pone.0307541.s003.pdf]

```
Sequence #: SIVRV1CG
Start=5858 End=7621
loaded 8173 nucleotides, cds_start 5858 to cds_end 7621

Energy 1: -34.710000: Start=2337 S1=6 S2=6 L1=1 L2=12 L3=0 End=2373

                    GAAACAAGCTTA       A

                    CCCCGG    ACCCCG

CCATCAGGGAAACGGACTGAGGGGCC    TGGGGCGG


Energy 2: -23.325000: Start=1502 S1=7 S2=5 L1=1 L2=11 L3=0 End=1537

                    CACATTAGCCA       C

                    ATACCCC    GTCAT

TAAAAACGGCTGCCTCTCAATATGGGG    TAGTAGA


Energy 3: -23.280000: Start=7255 S1=6 S2=6 L1=1 L2=29 L3=1 End=7309

                    GAGGCATCTGCTTAGCTTTACAGGAAAAA       G

                    GGACGA  G  ACGACA

CAAAACAGAAGAGGATTAGATCTGCT     TGCTGTTT


Energy 4: -22.750000: Start=7264 S1=6 S2=7 L1=4 L2=20 L3=0 End=7313

                    TAGCTTTACAGGAAAAATGC       GCAG

                    TCGTCT    ACGGAGG

AGAGGATTAGATCTGCTGACAGCAGA    TGTTTCTAC


Energy 5: -22.320000: Start=7255 S1=5 S2=6 L1=4 L2=6 L3=0 End=7286

                    GAGGCA       TGAC

                    GGACG    AGACGA

CAAAACAGAAGAGGATTAGATCTGC    TCTGCTTA


Energy 6: -22.140000: Start=3520 S1=5 S2=6 L1=1 L2=13 L3=1 End=3556

                    TGGCTATCCCACA       T

                    GGCCT  C  TCCCGT
```

ATTAATGGGAGCTTTACAACCTGGA AGGGTATC

Energy 7: -22.025000: Start=6081 S1=5 S2=8 L1=2 L2=18 L3=0 End=6126

CCCACTACAGCCAGCCCC CA

TCATC TGTGTGTG

TTCAGTAACCAACTCCCTAAAGTGG ACACATATAG

Energy 8: -21.995000: Start=6588 S1=6 S2=6 L1=2 L2=5 L3=0 End=6618

TGCTT AA

GTCGGT CGTTAG

TGTTTCCTCCCAACGCCAATTAGCCG GCGGTCAG

Energy 9: -21.875000: Start=7268 S1=6 S2=5 L1=3 L2=15 L3=0 End=7307

AGCTTTACAGGAAAA GGA

TTCGTC TACGG

GATTAGATCTGCTGACAGCAGAGCAG ATGCTGT

Energy 10: -21.485000: Start=2683 S1=5 S2=8 L1=2 L2=8 L3=0 End=2718

CCTACCTC AG

TCCTA ATTAACTC

TTACTGTTCCTCAAGGCAACAGGAT TAATTGAGAC

Energy 11: -21.215000: Start=1744 S1=5 S2=6 L1=4 L2=30 L3=0 End=1799

TTGCTCAAATTCAAGCGGCTGCTACAAAAG ATGA

TGTTA GGGCCT

TCTAGCACTGATGCACAAATGCAAT CCTGGAGA

Energy 12: -21.005000: Start=206 S1=5 S2=8 L1=2 L2=33 L3=0 End=266

TTGCCACCATTAAACGAGACTTGATCAGAACAC AT

ACGCA ACAGAATA

```
CTCCCCTCCCACTTACTGCCTGTGT    TGTCTTGTCT

Energy 13: -21.005000: Start=8029 S1=5 S2=8 L1=2 L2=33 L3=0 End=8089
                    TTGCCACCATTAAACGAGACTTGATCAGAACAC       AT
                    ACGCA    ACAGAATA
CTCCCCTCCCACTTACTGCCTGTGT    TGTCTTGTCT

Energy 14: -20.600000: Start=6081 S1=5 S2=7 L1=2 L2=17 L3=1 End=6124
                    CCCACTACAGCCAGCCC       CA
                    TCATC  T  GTGTGTG
TTCAGTAACCAACTCCCTAAAGTGG     CACACATAT

Energy 15: -20.520000: Start=6369 S1=8 S2=5 L1=4 L2=9 L3=0 End=6407
                    AGGGCCTCA       CACA
                    GGAGGTAG    TCTGT
GGTTTGCTGGAATAGCCAACCCTCTGTT    AGATAAG

Energy 16: -20.320000: Start=3314 S1=7 S2=6 L1=1 L2=12 L3=1 End=3353
                    AGTGAAAAACTT       C
                    CCAATTA  C  CGGTGA
AAATCAGACGAACCTGTCTGGGTTGAT     GCTGCTGC

Time taken to run: 9 seconds 42 milliseconds
-------------------------------------------------
```
